# Performance–Complexity Trade‐Offs in Battery Lifetime Prediction with Task‐Aware Transformers

**DOI:** 10.1002/advs.202524179

**Published:** 2026-07-16

**Authors:** Jingyuan Zhao, Misheng Cai, Zhenghong Wang, Yan Wang, Yuqi Li, Bo Dong, Hewu Wang, Andrew F. Burke, Simona Onori, Stephen J. Harris

**Affiliations:** ^1^ Institute of Transportation Studies University of California Davis Davis California USA; ^2^ School of Automotive and Traffic Engineering Hubei University of Arts and Science Xiangyang Hubei China; ^3^ Qingdao University of Technology Qingdao Shandong China; ^4^ Department of Materials Science and Engineering Stanford University Stanford California USA; ^5^ Department of Control Science and Engineering Changchun University of Technology Changchun Jilin China; ^6^ School of Vehicle and Mobility Tsinghua University Beijing China; ^7^ Department of Energy Science and Engineering Stanford University Stanford California USA; ^8^ Energy Storage and Distributed Resources Division Lawrence Berkeley National Laboratory Berkeley California USA

**Keywords:** battery, health, flash‐attention, sparse‐attention, transformer

## Abstract

Accurate battery lifetime prediction is essential for improving reliability and safety in energy storage systems. However, balancing predictive accuracy, inference latency, and energy consumption remains challenging. We introduce FAST‐BatPro, a Flash‐Attention Sparse Transformer for Battery Prognosis. This task‐aware architecture combines convolutional feature extraction with dual attention mechanisms to enable robust, efficient, and scalable prediction. The 1.869‐million‐parameter model is evaluated from both perspectives. Across four datasets comprising more than 240,000 cycles across chemistries, FAST‐BatPro demonstrates consistent performance across fast‐charging and discharging protocols, temperature variations, and chemistry‐dependent degradation behaviors. With limited early‐cycle data, it achieves high accuracy, with coefficient of determination values approaching or exceeding 0.90 in most test settings. It maintains an inference time of 0.103 s and requires 1.65 billion FLOPs per battery over the full lifecycle. Hidden‐dimension scaling identifies a compact configuration that preserves comparable accuracy while reducing inference latency, FLOPs, and energy consumption by 12.6%, 68.1%, and 12.6%, respectively. Module‐level pruning further shows that a feed‐forward‐network‐pruned lightweight variant improves accuracy while reducing inference latency, FLOPs, and energy consumption by 11.65%, 54.64%, and 11.65%, respectively, indicating that FAST‐BatPro can serve as a reference architecture for identifying task‐specific redundancy and guiding efficient AI model design for battery diagnostics and predictive maintenance.

## Graphical abstract

FAST‐BatPro integrates convolutional feature extraction, flash Attention, and sparse attention for efficient battery lifetime prediction. Using limited early‐cycle data across multiple chemistries and operating conditions, it achieves robust accuracy while reducing inference latency, computational cost, and energy consumption. The framework provides a compact and scalable route toward practical battery diagnostics and predictive maintenance.

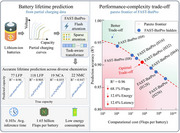

## 1 Introduction

Rechargeable batteries play a vital role in modern technologies owing to their high energy density and long service life, enabling diverse applications across multiple sectors [1, 2]. However, over time, they undergo irreversible degradation that impairs their performance [3]. Accurate health diagnostics and performance optimization are essential for ensuring operational safety and prolonging battery life, with remaining useful life (RUL) prediction and prediction of the failure distribution serving as core objectives [4, 5, 6, 7]. Prediction of RUL and failure distributions also facilitates a deeper understanding of complex battery systems, supports optimization in manufacturing processes, and contributes to reducing material waste and improving sustainability [8, 9, 10].

Currently, predictive diagnostic models for lithium‐ion batteries can be broadly categorized into three types: physical models, empirical models, and data‐driven models [11]. Physical models aim to simulate aging mechanisms based on first‐principles representations of internal electrochemical and physical processes. These models typically incorporate mathematical formulations describing key degradation phenomena, such as solid electrolyte interphase growth [12, 13], lithium plating [14, 15], and cathode phase transition [16, 17], and are often coupled with kinetic and diffusion equations [18, 19] to capture the spatiotemporal evolution of battery performance. Despite their strong interpretability, the complexity and computational cost associated with physical models hinder their scalability and widespread application [20]. Empirical models employ heuristic formulations to approximate battery aging behavior, with representative examples including the weighted ampere‐hour model. While these approaches allow rapid estimation of basic performance characteristics, they remain limited in capturing the nonlinear dynamics and diverse degradation pathways encountered under real‐world conditions [21, 22].

Compared to physics‐based and empirical models, data‐driven approaches, particularly those leveraging machine learning, have gained increasing attention for their ability to learn degradation patterns directly from data without requiring prior knowledge of underlying mechanisms [23, 24, 25, 26, 27]. Classical machine learning models, such as support vector machines [28], Gaussian process regression [29], relevance vector machines [30], extreme learning machines [31], and random forest regression [32] have been extensively applied to estimate the aging state of batteries. A landmark study demonstrated early prediction of cycle life by integrating structured data generation with regularized machine learning models, such as Lasso and Elastic Net, to mitigate overfitting and emphasize key features [33]. Follow‐up work further introduced data‐efficient optimization frameworks combining early‐cycle prediction models with Bayesian optimization to identify optimal fast‐charging protocols, reducing experimental time from over 500 days to just 16 [34]. These developments underscore the transformative potential of machine learning in accelerating battery design and performance evaluation.

In recent years, RUL prediction of battery systems under complex operating conditions has moved from single point estimation toward integrated modelling that jointly considers predictive accuracy, uncertainty quantification and computational efficiency. Existing studies have mainly focused on probabilistic lifetime prediction to address the stochastic nature of degradation processes, the limited observability of health indicators and the scarcity of lifetime labels. For example, methods combining Bayesian deep learning with nonlinear Wiener processes can quantify the uncertainty of health indicator prediction and enable probabilistic RUL prediction through online updating of degradation models [35]. Frameworks based on Bayesian updating and probabilistic model integration further fuse the posterior distributions of multiple degradation models and identify the RUL peak and reliability profile through grid sampling, thereby supporting probabilistic lifetime prediction and reliability assessment under limited prior knowledge [36]. To describe the uncertainty of lithium‐ion battery capacity degradation, methods such as ARIMAX, LQR, B‐MLR and BB‐MLR have been used to estimate RUL distributions, with point value extraction introduced to meet deterministic prediction requirements [37]. In addition, probabilistic ensemble frameworks using capacity and equal discharge voltage difference time interval as health indicators employ MONESN to track nonlinear battery degradation, enabling both direct and indirect RUL prediction with explicit uncertainty characterization [38]. As battery systems are increasingly deployed in electric vehicles, energy storage systems, and online monitoring scenarios, RUL prediction models are expected to deliver high accuracy while satisfying engineering requirements for online updating, low computational complexity, and fast inference. Recent studies have therefore incorporated both predictive performance and computational efficiency into model design. For instance, an online RVM method based on incremental optimization improves multistep RUL prediction through dynamic training and online learning while reducing computational complexity [39]. For energy storage systems, XGBoost models using temperature variables and hyperparameter optimization improve both prediction accuracy and computational efficiency [40]. Multistage feature adaptive meta models coupled with BiLSTM‐VAE generative models further enhance data representation and sample availability, thereby improving prediction accuracy, generalization and computational efficiency [41]. These studies indicate that battery lifetime prediction is shifting from the sole pursuit of low prediction error toward an integrated modelling paradigm that jointly considers accuracy, model complexity and computational efficiency. For BMS applications, model evaluation should extend beyond error metrics. Parameter count, FLOPs, inference time, memory footprint and energy consumption also quantify the resource cost of models in practical deployment. However, these metrics should be interpreted in relation to the target hardware platform. In this study, the reported inference time and energy consumption were measured on an NVIDIA RTX 4090 workstation and are used mainly as comparative computational‐efficiency indicators across model configurations, rather than as direct embedded‐BMS deployment benchmarks. Recent lightweight studies further support this view. For example, RUL prediction models combining enhanced ShuffleNet with physical constraints can achieve high accuracy within 2 to 3 cycles while maintaining low model complexity [42]. Methods integrating the Lotus optimization algorithm with RBF neural networks have achieved high accuracy on the NASA and Oxford datasets with only approximately 0.36 million FLOPs and 730 inferences per second [43]. Resource efficient battery health estimation models that combine FlashAttention‐2 with CNN based local feature extraction can estimate capacity accurately from partial charging data while maintaining short inference time, low power consumption, and a small memory footprint [44]. The BOTC framework further integrates Transformer, CNN and Bayesian optimization to improve prediction accuracy and robustness while reducing parameter count, FLOPs and inference time [45]. These results show that the practical value of a model depends not only on prediction error, but also on computational burden, runtime efficiency and resource consumption. Similar efficiency constraints also exist in broader prognostics and health management tasks. AttnPINN combines self‐attention, low dimensional feature mapping, and physics informed regularization to achieve accurate and interpretable aircraft engine RUL prediction with a small number of parameters [46]. DLformer dynamically selects feature representations of different lengths, reuses previously learned features and controls the computational path through a confidence‐based strategy, substantially improving inference speed with limited accuracy loss [47]. Therefore, in battery lifetime prediction and health management tasks, model complexity should be matched to task requirements, input signal characteristics, and available computational resources, instead of being driven by model scale or prediction error alone.

Despite these advancements, classical machine learning models still face limitations when dealing with incomplete, noisy, or irregularly sampled data and unknown boundary conditions [48]. Additionally, they often rely on handcrafted feature engineering, which requires significant domain expertise. In contrast, deep learning methods offer an end‐to‐end alternative capable of modeling complex, nonlinear degradation patterns and processing high‐dimensional, multimodal input data [49, 50, 51]. Recent studies have introduced deep ensemble architectures that integrate domain adaptation and inter‐cell learning mechanisms, significantly enhancing generalization across varying battery types and usage conditions [52, 53]. However, beyond generalization, effective use of limited early‐cycle data remains a central challenge in battery lifetime prediction. To address this issue, several deep‐learning‐based feature engineering frameworks have been developed. For example, convolutional sparse autoencoders can process high‐dimensional tensors integrating voltage, current, and temperature and extract latent spatiotemporal features from very early cycles for rapid lifetime classification [54]. Another study proposed an attention‐assisted temporal convolutional memory‐augmented network, which reconstructs degradation patterns from only 10 incomplete cycles through dual‐channel attention and memory augmentation, enabling robust RUL prediction across multiple battery chemistries [55]. Despite the rapid development of specialized architectures, CNNs and LSTMs remain widely used in battery health diagnostics because of their respective abilities to extract spatial and temporal features [56, 57]. However, CNNs are effective for local pattern recognition but have limited capacity to capture long‐range dependencies, whereas LSTMs are suitable for sequence modelling but face vanishing gradients and high computational cost under long sequences and fluctuating operating conditions.

To overcome these limitations, Transformers have become an important alternative for time‐series modelling. Originally developed for natural language processing, the self‐attention mechanism of the Transformer efficiently captures both local and global dependencies, making it suitable for representing complex battery degradation patterns in long time‐series data [58]. Based on this capability, a Transformer‐based multivariate prognosis method has been developed for battery SOH prediction by jointly modelling health indicators such as capacity, energy, coulombic efficiency, open‐circuit voltage and equivalent circuit model parameters. In this method, a spacetime Transformer captures both cross‐variable correlations among health indicators and their temporal evolution over cycling, with validation conducted on fast‐charging lithium iron phosphate datasets and the Cell Analysis, Modeling, and Prototyping battery dataset [59]. Beyond this representative work, recent studies have further demonstrated the effectiveness of customized Transformer networks across a broader range of battery applications, including health estimation [60], lifetime prediction [61], fault diagnosis [62], and early thermal runaway detection [63], by enhancing attention mechanisms and incorporating temporal encoding.

**FIGURE 1:**
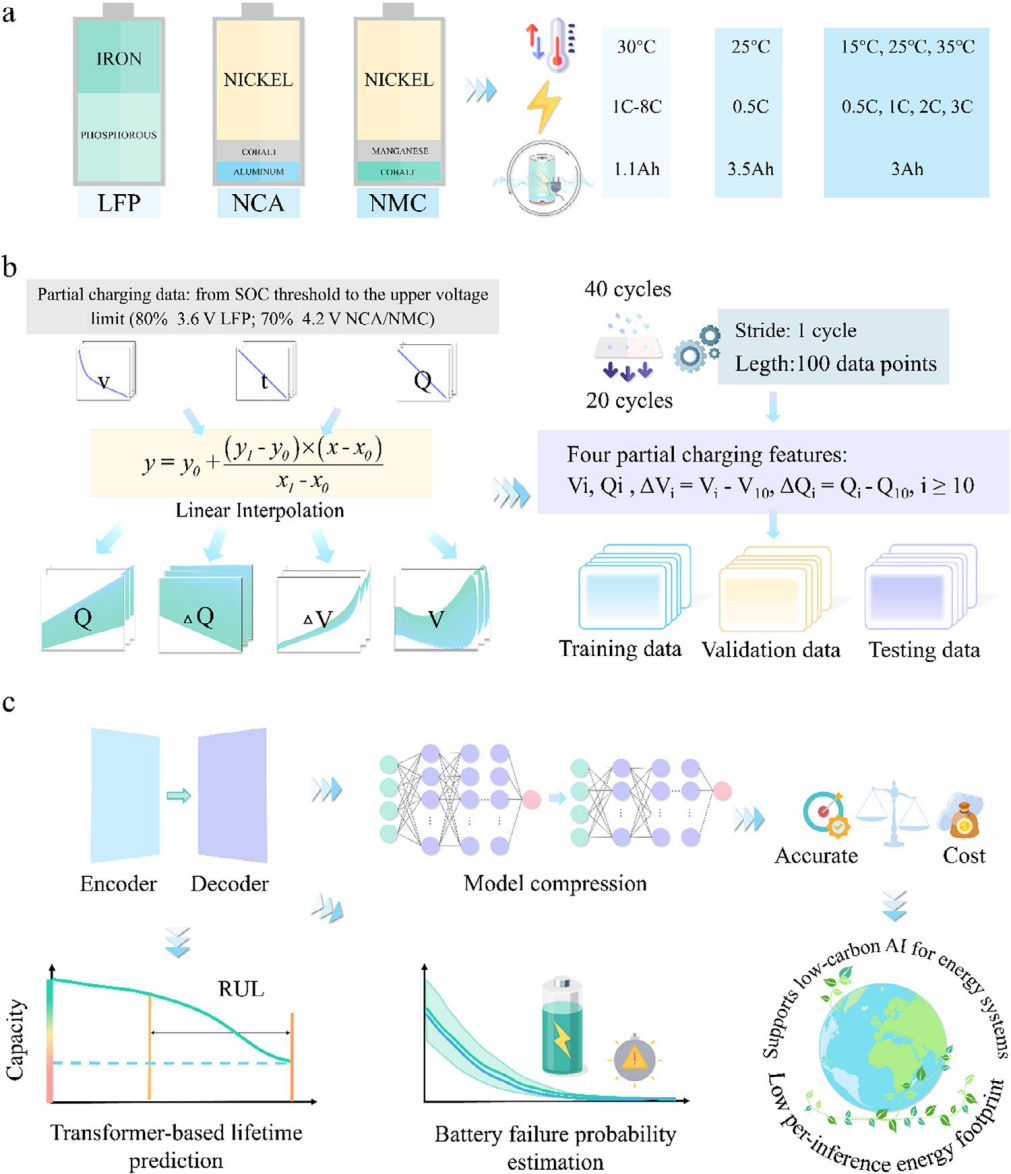
Flowchart of the FAST‐BatPro framework. (a) Input datasets include LFP, NCA, and NMC cells tested under different temperatures, current rates, and capacity ranges. (b) Partial charging data are extracted from chemistry‐specific SOC windows to the upper voltage limit, followed by curve fitting, linear interpolation to 100 points, differential feature construction, normalization and sliding‐window segmentation. The resulting voltage, capacity, differential voltage, and differential capacity features are divided into training, validation, and testing sets. (c) The processed sequences are used by the FAST‐BatPro encoder‐decoder model for RUL prediction and battery failure probability estimation. Model compression is further used to evaluate the trade‐off between prediction accuracy and computational cost.

Overall, Transformer‐based deep learning provides clear advantages in modelling complex spatiotemporal dependencies and has shown the ability to infer battery lifetime rapidly from limited early‐cycle data. However, existing studies mainly focus on prediction accuracy under data‐scarce conditions, while the trade‐off between predictive performance and computational cost in practical deployment remains insufficiently explored. As transportation systems move toward low‐carbon and intelligent operation, battery lifetime prediction models require high accuracy, computational efficiency, and practical resource awareness. The intrinsic complexity of real‐world battery degradation data often demands sophisticated model architectures, which can increase inference latency, energy consumption, and resource requirements. To address these issues, this work proposes FAST‐BatPro, a customized end‐to‐end Flash‐Attention Sparse Transformer for battery lifetime prognosis. Through task‐aware Transformer design, FAST‐BatPro systematically balances predictive accuracy and computational efficiency, supporting computationally efficient battery lifetime prediction with potential relevance to resource‐constrained applications (Figure 1).

This study aims to improve battery lifetime prediction accuracy and model generalization while systematically examining the trade‐off between predictive performance and computational complexity in large‐scale Transformer models. To this end, we develop a CNN‐Transformer hybrid framework for battery lifetime prediction, establishing an end‐to‐end prognostic system. The model uses CNN‐based local feature extraction, flash self‐attention in the encoder, and masked probabilistic sparse attention in the decoder to capture multiscale degradation patterns and long‐range temporal dependencies from battery cycling data. To accommodate input data with different levels of fidelity, a standardized data preprocessing pipeline is designed to improve scalability and robustness across diverse operating scenarios. In feature representation, the CNN component captures local degradation patterns, whereas the Transformer encoder further models global temporal evolution. Meanwhile, the integration of flash attention and sparse attention improves memory efficiency and computational scalability, making the model suitable for large‐scale battery data processing. Although Transformer models offer strong predictive capability, excessive model scaling often brings limited accuracy gains at high computational cost. To address this issue, this study systematically analyses model scaling behaviour and reveals the trade‐off among predictive performance, inference latency, and energy consumption. The results define practical boundaries for model complexity and provide guidance for designing scalable and resource‐efficient lifetime prediction models in battery management systems.

## 2 Results

### 2.1 Battery Aging Dataset and Partitioning

**FIGURE 2:**
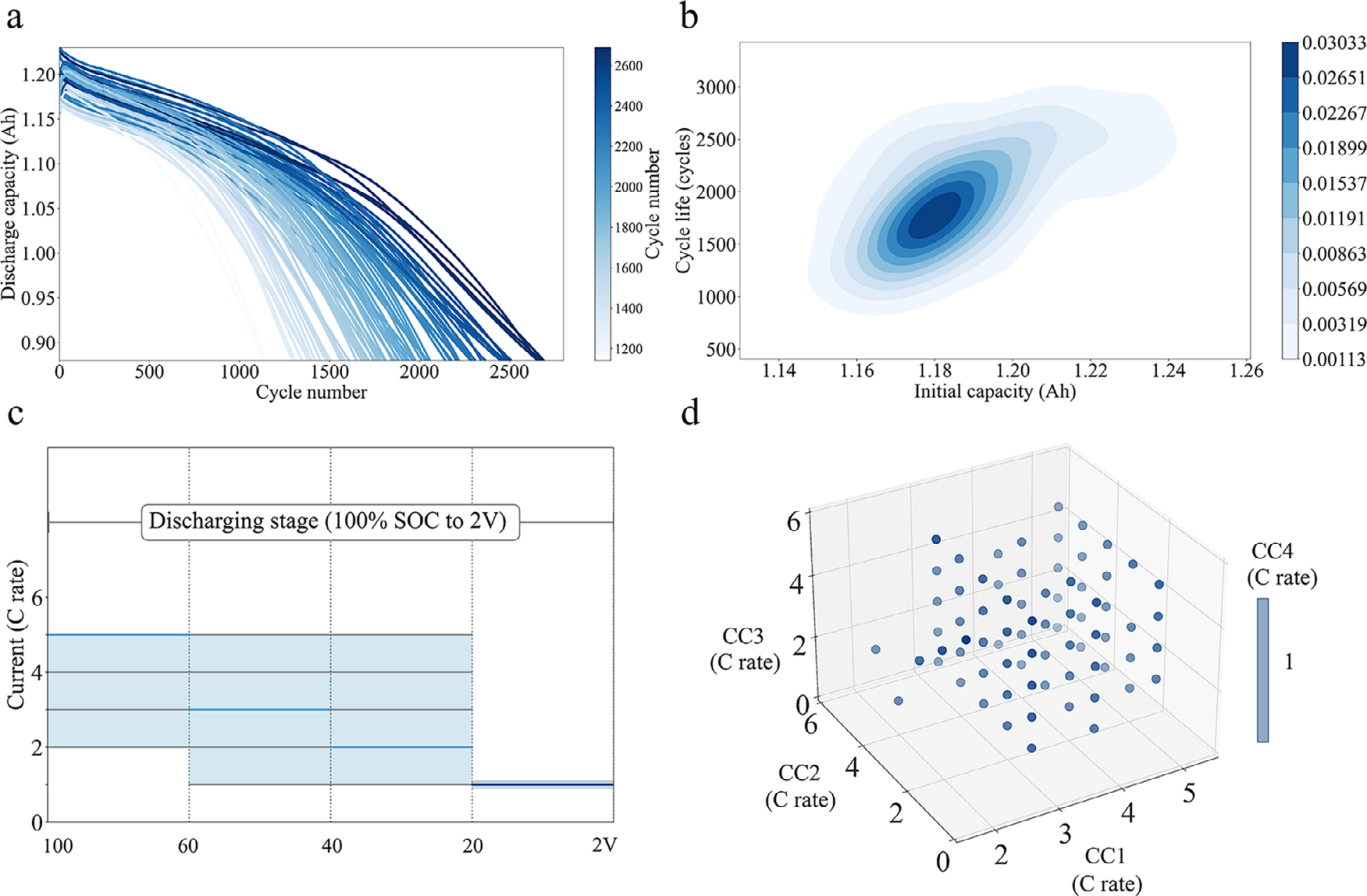
Data characteristics for battery cycle aging of 77 LFP batteries. (a) Change in discharge capacity versus cycle number for 77 batteries. (b) Joint distribution of cycle life and initial capacity for 77 batteries, with color gradation representing data density. (c) Current rate ranging from 80% SOC to 2 V during the discharge stage. (d) Current rate distribution during the four constant‐current steps.

Two large battery cycling datasets using multi‐step constant current–constant voltage (CC‐CV) protocols are employed in this study. The first dataset (Figure 2) includes 77 LFP/graphite batteries, each with a nominal capacity of 1.1 Ah and a nominal voltage of 3.3 V [64]. The dataset follows a four‐step discharge and a three‐step charging. Figure 2a illustrates the capacity degradation in cycle number, with the color of the curves scaled according to cycle life, ranging from 1100 to 2700 cycles. The average cycle life is 1898 cycles, with a standard deviation of 387 cycles. Figure 2b shows the joint distribution of cycle life and initial capacity for all the batteries. The 77 distinct multi‐step current rate ranges and the current rate distribution during the fast‐discharging stage are shown in Figure 2c,d, respectively. Notably, in this dataset, each original file corresponds to the complete charge and discharge cycling data of one individual cell, and the file name serves as the identifier of that cell sample in the original dataset. Therefore, to ensure consistency and traceability between the presented results and the original data files, the original file names have been retained as battery labels to distinguish different cell samples. For example, “8‐6” denotes the individual cell sample corresponding to the original data file named “8‐6”. Furthermore, the second dataset includes 118 LFP/graphite batteries, each having a nominal capacity of 1.1 Ah and a rated voltage of 3.3 V [33]. These batteries completed between 150 and 2300 cycles, with an average cycle life of 806 and a standard deviation of 377. Figure S1a (Supporting Information) shows the capacity degradation curves, where the color represents the cycle life of the batteries. Figure S1b (Supporting Information) illustrates the distribution of initial discharge capacity and cycle life. Each battery followed either a single‐step or two‐step fast charging protocol, along with a typical constant current (CC) discharge strategy, covering multiple charging methods and one discharge method. The first CC step was conducted at C‐rates ranging from 1C to 8C, whereas the second CC step used C‐rates ranging from 3C to 6C. (Figure S1c, Supporting Information). Additionally, Figure S1d (Supporting Information) presents the changes in voltage and current over time for a single battery during a complete charge‐discharge cycle. In addition, to further evaluate the capability of the model to predict the RUL across different battery chemistries, two additional datasets were employed. Dataset 3 consists of 19 lithium nickel cobalt aluminum oxide (NCA) cells, each with a nominal capacity of 3.5 Ah and a nominal voltage of 3.6 V. These cells were cycled under identical charge and discharge current rates of 0.5 C and 1 C, respectively, until the capacity declined to approximately 92% or lower relative to the nominal capacity. Charging was conducted using a CC protocol up to 4.2 V, followed by CV charging until the current dropped to 0.05 C. Discharging was subsequently performed using the CC mode down to a cutoff voltage of 2.65 V. All cycling procedures were carried out at an ambient temperature of 25 °C, and the number of cycles ranged from 107 to 207, with an average cycle life of 173 cycles and a standard deviation of 28 cycles [65]. Dataset 4 includes 22 lithium nickel manganese cobalt oxide (NMC) cells, each with a nominal capacity of 3 Ah and a nominal voltage of 3.6 V [66]. The cells were charged at 0.5 C under a CC–CV protocol and discharged at 0.5 C, 1 C, 2 C, or 3 C until the capacity declined to approximately 81% or lower. The same CC–CV charging procedure was applied: charging to 4.2 V using CC mode, followed by CV charging until the current dropped to 0.05 C, and subsequent CC discharging to a cutoff voltage of 2.0 V. These tests were performed at ambient temperatures of 15, 25, and 35 °C, with cycle counts ranging from 388 to 1321. The battery dataset had an average cycle life of 674 cycles and a standard deviation of 210 cycles. The detailed experimental conditions are summarized in Table S1 (Supporting Information). Dataset‐specific partitions were generated using a fixed random seed and are summarized, together with unified dataset notation, in Table 1. The datasets were partitioned independently, with dataset 2 using a batch‐aware splitting strategy to account for its three‐batch structure.

**TABLE 1:** Dataset partitioning strategies and naming conventions used in this study.

Datasets	Battery types	Training set	Validation set	Test set	Partitioning strategy	Cell‐label notation^a^
Dataset 1	LFP	57	0	20	Random cell‐level split	Original file label, e.g., 8‐6
Dataset 2	LFP	75	7	36	Batch‐aware split	Batch and cell index, e.g., a5
Dataset 3	NCA	11	3	5	Random cell‐level split	Cell index, e.g., #1
Dataset 4	NMC	12	4	6	Random cell‐level split	Protocol‐based label, e.g., 0.5‐1C_a

^a^ ***Note***: Cell labels were retained from the original dataset files to preserve traceability between reported results and source data.

### 2.2 FAST‐BatPro Architecture and Cross‐Dataset Prediction Performance

**FIGURE 3:**
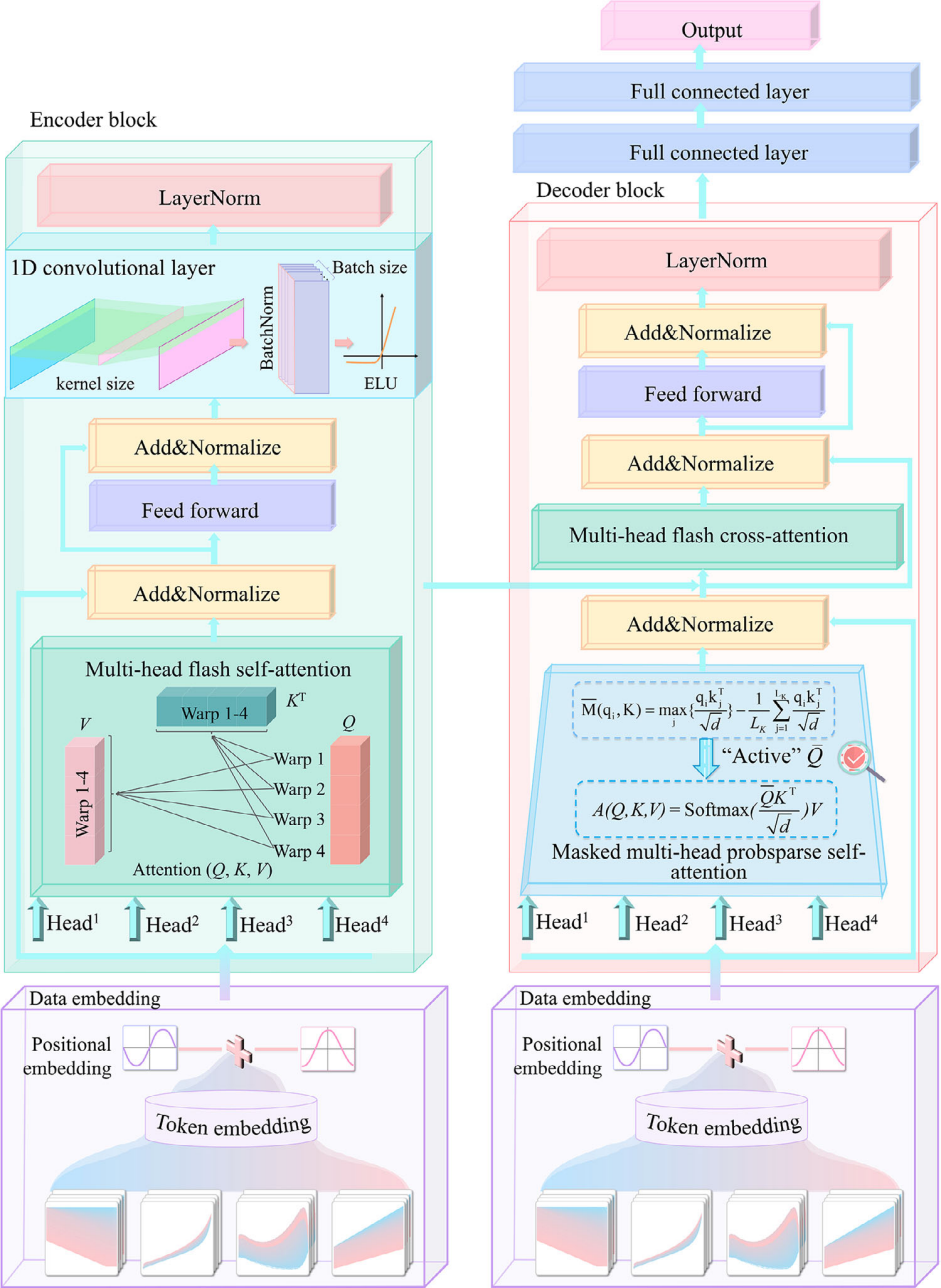
Structure of the FAST‐BatPro model. The model comprises an embedding module, an encoder module, a decoder module, and two fully connected layers. Specifically, the embedding module includes token embedding and positional embedding. The encoder module consists of multi‐head flash self‐attention layers and feed‐forward network layers, with residual connections and normalization layers added after each layer. The decoder module consists of masked multi‐head sparse self‐attention layers, feed‐forward network layers, and multi‐head flash cross‐attention layers, each followed by residual connections and normalization layers. Subsequently, both modules undergo final layer normalization. Finally, the two fully connected layers output the prediction targets.

The Transformer‐based multi‐fusion neural network architecture (Figure 3) comprises 1.869 million parameters. In the encoder, flash self‐attention is used to reduce memory usage through block‐wise attention computation. Multi‐head flash self‐attention, feed‐forward networks, residual connections, and layer normalization are used to encode low‐dimensional feature vectors with positional information. Residual connections help alleviate gradient vanishing in deep networks, whereas layer normalization improves training stability and convergence efficiency. Because the conventional Transformer mainly captures global correlations in long sequences and may insufficiently represent local degradation features, a one‐dimensional CNN is incorporated into the encoder to strengthen local feature extraction. This design enables the model to capture both local dependencies and global aging patterns in battery time‐series data. The decoder adopts masked self‐attention and flash cross‐attention. Masked self‐attention prevents information leakage from future positions, whereas flash cross‐attention integrates global information from the encoder output. Finally, layer normalization further stabilizes the encoded feature distribution and provides consistent inputs to the decoder, thereby improving predictive performance and robustness for accurate battery RUL prediction.

**FIGURE 4:**
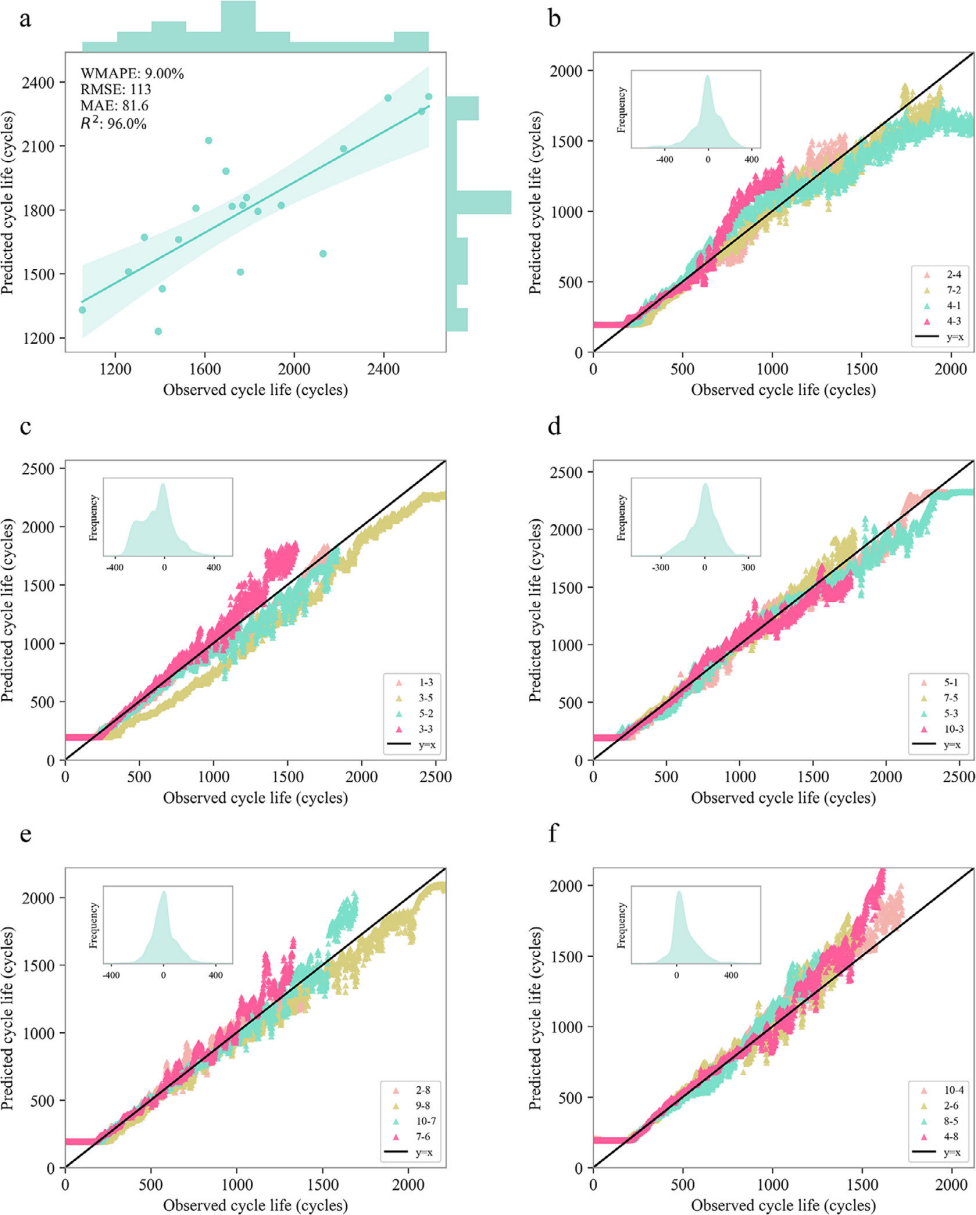
Prediction results and error distribution for 20 test cells under distinct discharging protocols in dataset 1. (a) displays the overall distribution of predicted RUL versus actual RUL for the 20 batteries. (b)‐(f) show the cell‐level prediction results for different battery samples. The black line denotes the ideal prediction line, where the predicted cycle life equals the observed cycle life. Colored markers represent different battery samples, and the battery labels follow the original file names in dataset 1, such as “8‐1”. The inset in each panel shows the distribution of prediction residuals, reflecting the bias and dispersion of model errors.

To systematically evaluate the predictive accuracy and generalization capacity of the proposed model under diverse charging and discharging protocols, experiments were conducted on two independent battery datasets. The proposed model exhibits robust predictive accuracy on dataset 1, yielding a root mean squared error (RMSE) of 113 cycles, a coefficient of determination (R^2^) of 0.960, a weighted mean absolute percentage error (WMAPE) of 9%, and a mean absolute error (MAE) of 81.6 cycles on the test set (Figure 4). The definitions of the four metrics are provided in Supplementary Note 1. Notably, the model maintains comparable performance under a second random cell‐level split, achieving an RMSE of 107 cycles (Figure S2, Supporting Information).

Under the initial dataset 2 split, the model likewise demonstrated stable performance, yielding an RMSE of 71 cycles, an R^2^ of 0.905, a WMAPE of 12.3%, and an MAE of 54 cycles (Figure S3, Supporting Information), indicating robust generalization across different data distributions. To mitigate potential evaluation bias introduced by the partial overlap of charging protocols between the training and test sets in dataset 2, a more stringent validation protocol was implemented. In this configuration, all charging protocols appearing in the test set were entirely excluded from the training set, thereby ensuring protocol‐level independence between the two subsets. By contrast, dataset 1 is composed of samples operating under distinct discharging protocols and thus naturally satisfies protocol independence, requiring no additional preprocessing. Under this protocol‐exclusion setting, the model retained stable predictive performance on dataset 2, achieving an RMSE of 55 cycles, an R^2^ of 0.879, a WMAPE of 13.9%, and an MAE of 42.5 cycles (Figure S4, Supporting Information), further confirming its capacity for cross‐protocol generalization in unseen conditions.

To further investigate the impact of data splitting strategies on model performance, the distributional similarity between training and test sets was assessed. Figure S5a,c,e (Supporting Information) visualizes the normalized Euclidean distance matrices computed from three normalized charging‐behavior features, namely mean charging voltage, mean charging capacity, and mean cycle count, in the constructed three‐dimensional feature space (see Supplementary Note S3.2). Most sample pairs exhibited distances within the range of 0.2 to 0.6, suggesting a moderate level of feature‐space similarity; however, several distances exceeded 0.8, indicating that some test samples diverge considerably from the training set in the feature space. A closer inspection of the discharge capacity degradation trajectories (Figure S5b,d,f, Supporting Information) reveals more pronounced heterogeneity in aging behaviors across cells, particularly in terms of degradation onset, capacity fade rate, and nonlinear progression patterns. It is noteworthy that in the cycle life prediction task on dataset 2, although RMSE and MAE remained stable, the R^2^ and WMAPE metrics showed a marked decline. This discrepancy may primarily result from a high proportion of short‐lifespan batteries, which significantly affects the overall variance structure and limits the ability of the model to explain total variability. Moreover, WMAPE is highly sensitive to small target values, and short‐lifespan samples tend to amplify relative errors during calculation, thereby reducing the overall evaluation score.

Building upon the cross‐protocol validation framework, the model was further evaluated on two additional datasets encompassing ternary lithium battery chemistries under different temperature conditions, in order to assess the generalizability of the approach across material domains. For dataset 3, which includes cycling data from NCA‐based cells, the model achieved a RMSE of 16.7 cycles, a R^2^ of 0.855, a WMAPE of 17.1%, and a MAE of 13.2 cycles. In dataset 4, comprising NMC‐based batteries characterized by distinct electrochemical behavior, the model yielded a RMSE of 32.5 cycles, a R^2^ of 0.947, a WMAPE of 9.18%, and a MAE of 26.1 cycles (Figure S6, Supporting Information). These results collectively highlight the robustness of the model across diverse chemistries and cycling conditions, thereby reinforcing its potential for broader battery health estimation across heterogeneous experimental datasets.

### 2.3 Battery Failure Probability Estimation

**FIGURE 5:**
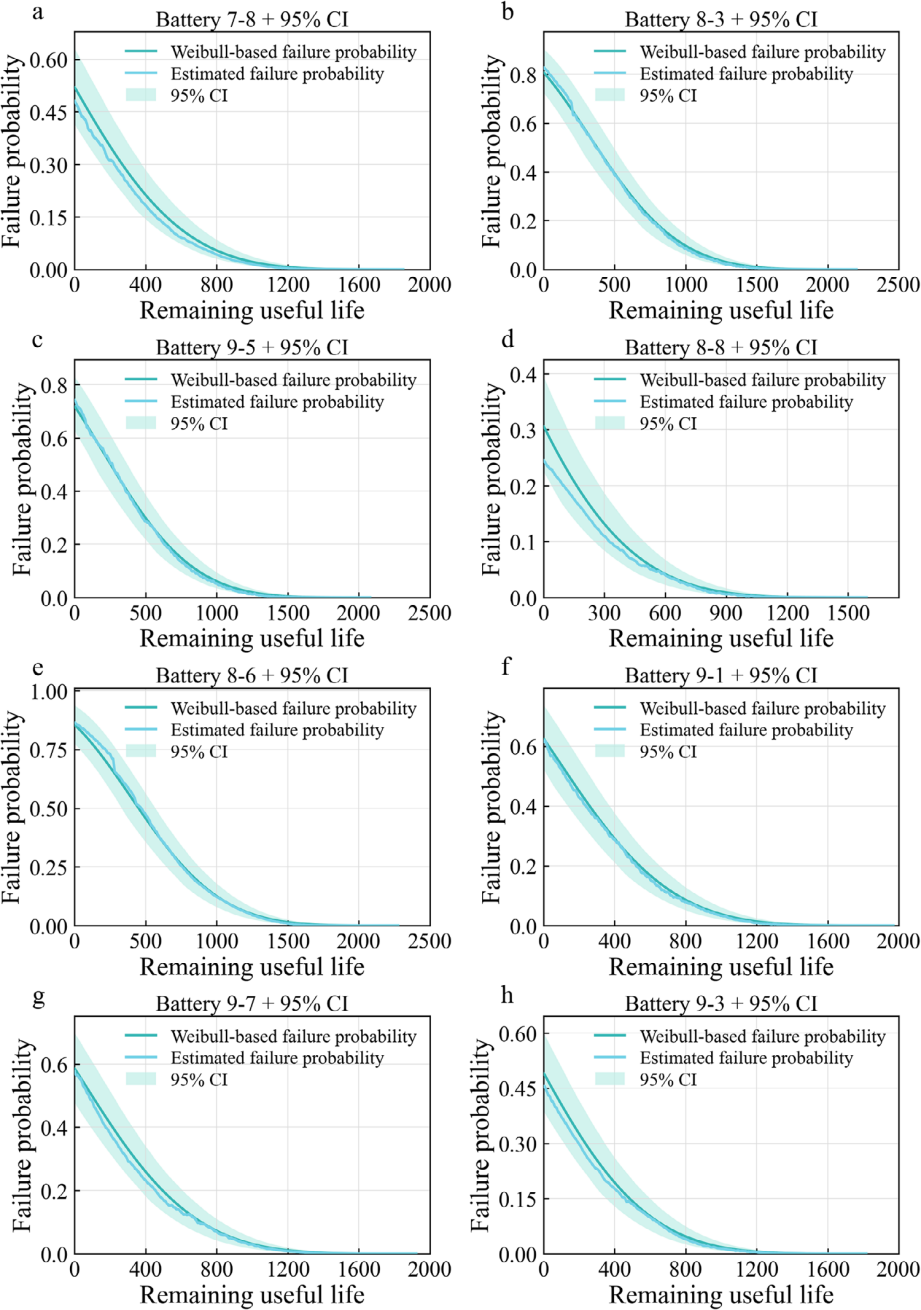
Estimated battery failure probability curves compared with Weibull‐based reference curves across 8 test samples (a–h). The cyan line represents the failure probability predicted by the deep learning model at each cycle. The dark turquoise line indicates the reference failure probability fitted using the Weibull distribution, while the shaded region shows the 95% confidence interval. Each subplot shows the agreement between model‐estimated failure probability and Weibull‐based reference curves across different test samples, indicating that the proposed model captures Weibull‐consistent population‐level failure trends. Battery labels, such as “7‐8” and “8‐3”, correspond to the original file names in dataset 1, with each file representing one individual battery sample.

In practical applications, the degradation behavior of individual battery samples exhibits considerable stochasticity and heterogeneity, influenced by microstructural properties at the material level, manufacturing inconsistencies, and operational factors such as charge/discharge rates and temperature fluctuations. As a result, conventional estimation methods based on expected lifetime fall short in capturing the distributional variability and risk associated with battery populations over extended service periods. To address this issue, the measured lifetime data were first fitted with a two‐parameter Weibull distribution to characterize population‐level failure patterns and their evolution. On this basis, a FAST‐BatPro‐based deep learning approach is developed to leverage partial charging features from individual samples and predict the failure probability distribution of the broader battery population (Figure 5 and Figure S7, Supporting Information). Specifically, a pretraining model was constructed using full‐lifetime data from 57 samples, and the decoder module was fine‐tuned using features and probabilistic labels from 19 cycles, with a learning rate of 2 × 10^−^^4^ over 200 epochs. The predicted failure probability curves for all 20 test samples fall within the 95% confidence interval of the Weibull fit, indicating consistency with the Weibull‐based reference curves. These results suggest that the proposed method can approximate Weibull‐consistent population‐level failure probability trends without complete lifecycle observations for the test cells. This approach provides a data‐driven framework for inferring population‐level degradation tendencies from limited individual sample information.

(1) $$f\left(t\right)=\frac{\beta }{\eta }{\left(\frac{t-\gamma }{\eta }\right)}^{\beta -1}exp\left[-{\left(\frac{t-\gamma }{\eta }\right)}^{\beta }\right]$$

where *β*>0 is the shape parameter that determines the form of the distribution, and *η*>0 is the scale parameter that controls its horizontal scaling. The distribution is defined over 𝑡≥0, under the assumption that failures may occur from the onset of the observation period.

### 2.4 Effects of Dataset Size and Distribution Shift on Prediction Performance

**FIGURE 6:**
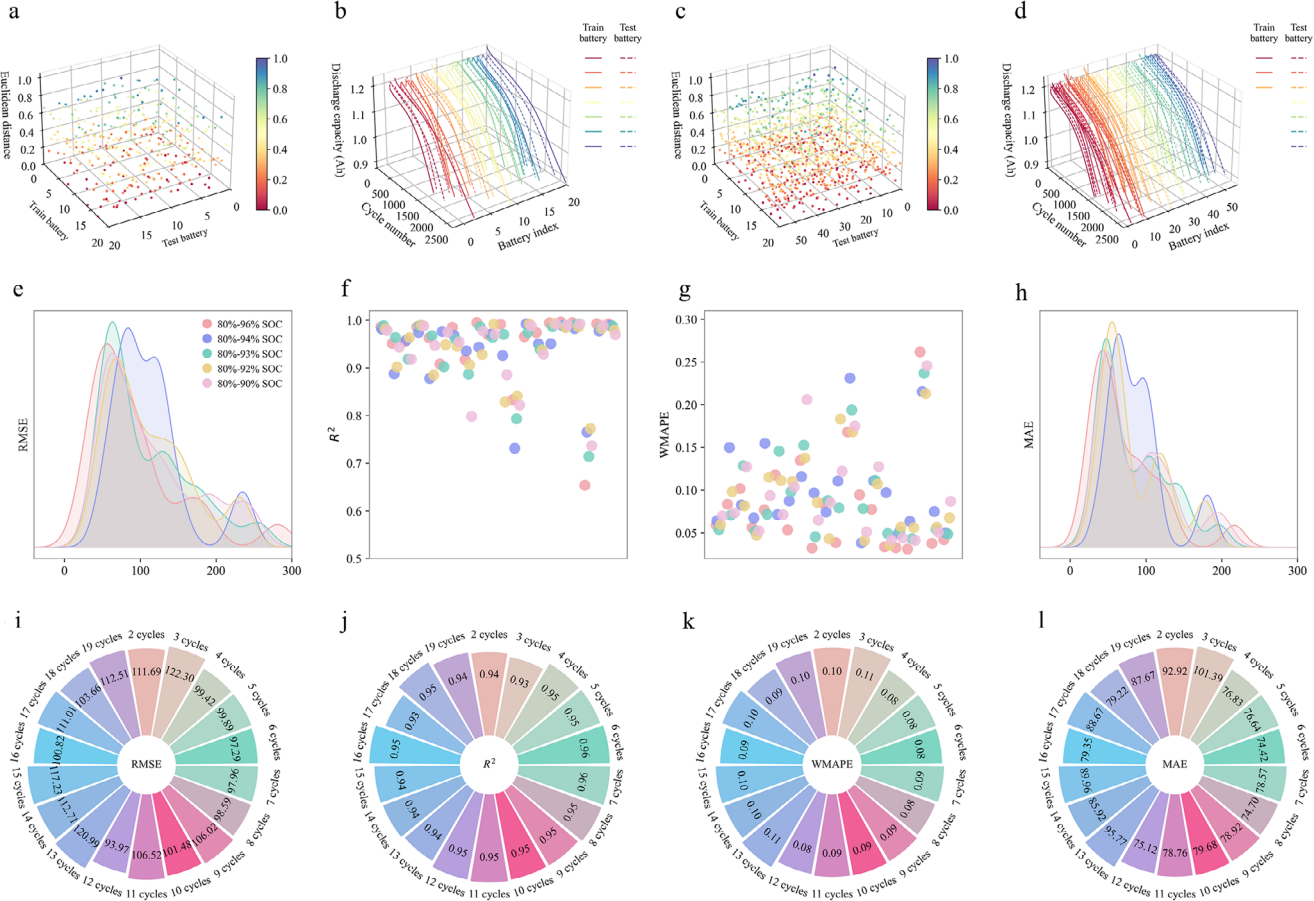
Dataset size configuration, effects of SOC input range and input sequence length on model prediction performance and data distribution. (a–d), data distribution and capacity degradation trajectories of dataset 1 under different training and testing size configurations (a) and (b) correspond to 20 training batteries and 20 testing batteries, whereas panels (c) and (d) correspond to 20 training batteries and 57 testing batteries. (a) and (c) show the Euclidean distance distributions between training and testing batteries, with color indicating normalized discharge capacity. (b) and (d) show the corresponding capacity degradation trajectories, where solid lines represent training batteries and dashed lines represent testing batteries. (e)‐(h), error distributions under different SOC input ranges. Charging data within approximately 80% to 96%, 80% to 94%, 80% to 93%, 80% to 92%, and 80% to 90% SOC were used as model inputs. Panels correspond to RMSE, R^2^, WMAPE and MAE, respectively. (i–l), model performance under different input sequence lengths. Radial bar plots show RMSE, R^2^, WMAPE, and MAE for input lengths from 2 to 19 cycles. Each sector represents one input length, and the value inside the sector denotes the corresponding metric.

To investigate the influence of training dataset size on the performance of RUL prediction models, two experimental schemes were designed. In the first scheme, the test set was fixed to 20 batteries, while the training set size was progressively reduced (57, 50, 40, 30, 20). This setup aimed to examine the variation in predictive accuracy under different training data volumes, thereby assessing the model learning capacity and the potential risks of underfitting or overfitting. The distributions of discharge capacity and cycle life in the training and test sets for each configuration are shown in Figure 6 and Figure S8 (Supporting Information). In addition, under different training–testing dataset configurations, the normalized Euclidean distance was computed using the mean charging voltage and capacity to quantify feature‐space similarity between the training and test sets (Supplementary Note 3.4 and Table S2, Supporting Information). In the second scheme (Figures S5a,b and  S9, Supporting Information; Figure 6), the proportions of the training set (40, 30, 20) and test set (37, 47, 57) were adjusted simultaneously to evaluate the impact of overall data volume variation on the model's generalization capability, particularly under limited data conditions. Model performance was quantified using RMSE and MAE, with the aim of capturing performance trends as data volume varied and identifying a potential saturation point. These results are intended to inform the rational selection of training dataset size in practice. The experimental results indicate that increasing the size of the training dataset does not lead to a strictly monotonic decrease in RMSE, but rather results in performance fluctuations within a specific range (Figure S10 and Table S3, Supporting Information).

Under limited training data conditions, particularly with 20–30 training cells, the model showed larger prediction errors, suggesting insufficient coverage of degradation variability. As the training size increased to 40–50 samples, the prediction error declined but remained variable, suggesting that data quality and representativeness influence learning effectiveness. When the training set included 50 samples, the model achieved the lowest RMSE of 101 cycles, WMAPE of 8.67%, and MAE of 76.8 cycles, whereas the full 57‐sample training set yielded the highest R^2^ of 0.960. This nonmonotonic trend suggests that adding more training samples does not necessarily improve all error metrics when the additional cells introduce heterogeneous aging trajectories, protocol‐dependent variability, or weaker feature‐space alignment with the test set. Therefore, model generalization is governed not only by training dataset size, but also by the quality, diversity, and representativeness of the training data. Moreover, results from the second experimental configuration demonstrate that a reduction in training set size accompanied by an expansion of the test set led to a gradual decrease in prediction accuracy. This trend highlights the adverse effects of dynamic data distribution shifts and reduced training coverage on model performance (Figure S11 and Table S3, Supporting Information).

### 2.5 Effects of SOC Window and Sampling Resolution

The generalization capability of the model was further investigated by training and testing it under a narrower range of operating conditions, achieved by reducing the voltage operating window and SOC window. Training with data from a restricted SOC range not only enhances data processing efficiency and response time but also improves practicality. Prediction tasks were conducted using capacity and voltage data from progressively narrowed SOC ranges, beginning with 80% to 96% SOC, corresponding to 80% SOC to 3.6 V, and decreasing to 80% to 90% SOC. The results indicate that the proposed model maintains stable performance across these reduced SOC ranges (Figure 6 and Table S4, Supporting Information), with RMSE, WMAPE, and MAE at 96 cycles, 8.27%, and 73.7 cycles, respectively, and an R^2^ value exceeding 0.93. This stability highlights the robustness of the multi‐fusion model even when applied to narrower, low‐fidelity charging data, reinforcing its critical role in assessing model performance and its applicability to real‐world diagnostic tasks. In addition, data sparsity experiments were conducted to further evaluate model robustness under reduced sampling resolution (Figure S12 and Table S5) in Supplementary Note S3.6. At a sampling interval of 70 s, the model achieved an RMSE of 122 cycles, an R^2^ of 0.934, a WMAPE of 10.7%, and an MAE of 96.4 cycles. These results indicate that the proposed model can maintain stable predictive performance under sparse data conditions.

### 2.6 Performance Sensitivity to Input Sequence Length

To systematically evaluate the impact of input sequence length on model performance, a sensitivity analysis was conducted by adjusting the sampling interval within a fixed window of 40 cycles. This configuration resulted in input sequences ranging from 2 to 19 cycles (Figure 6). The results show a nonmonotonic relationship between input sequence length and prediction accuracy, indicating that temporal granularity affects the ability of the model to represent battery degradation dynamics. RMSE varied across different input lengths and reached its lowest value of 93.97 at 12 cycles. Increased errors were observed for overly short sequences and some longer inputs, suggesting that both insufficient temporal information and redundant segments can weaken prediction stability. MAE followed a similar trend, with the best result of 74.42 obtained at 6 cycles. WMAPE remained relatively stable, mainly ranging from 8% to 11%, indicating consistent control of relative errors across different temporal configurations. R^2^ also remained high, approximately between 0.93 and 0.96, with better goodness of fit observed at 6 to 7 cycles. Overall, overly short sequences may fail to cover key aging stages, whereas excessively long sequences may introduce redundant or weakly informative segments. The model maintained stable predictive performance over input lengths from 2 to 19 cycles. Considering RMSE, MAE, WMAPE and R^2^ together, input lengths of 6 to 12 cycles provide a favorable balance between prediction accuracy and temporal information content.

### 2.7 Benchmarking against Deep Learning Baselines

To comprehensively assess the applicability of various deep learning approaches, this study compares multiple deep learning models (CNN, CNN‐Attention, CNN‐LSTM, LSTM, LSTM‐Attention, and Transformer) with the proposed FAST‐BatPro model in terms of both prediction accuracy (Supplementary Note S3.7). To systematically evaluate model performance, two experimental schemes were designed. In the balanced setting, the training set included 20 LFP batteries and the test set included 20 LFP batteries (Figure S13a,b, Supporting Information), allowing model performance to be assessed under similar data distributions and relatively ideal conditions. In the generalization challenge, the training set was fixed at 20 LFP batteries, whereas the test set was expanded to 57 LFP batteries (Figure S13c,d, Supporting Information), enabling evaluation of model generalization under substantially different data distributions and unseen samples. Experimental results demonstrate that FAST‐BatPro outperforms the baseline models in prediction accuracy (Figure S13 and Table S6, Supporting Information). However, the design objective of FAST‐BatPro extends beyond accuracy enhancement, with an emphasis on computational scalability and reduced resource consumption. Specifically, FAST‐BatPro integrates probabilistic sparse attention and flash attention mechanisms to streamline attention computation, thereby significantly reducing computational complexity while enhancing efficiency and resource utilization. Nevertheless, despite its more intricate architecture, the increase in computational complexity does not yield a substantial improvement in prediction accuracy. This finding suggests that in battery health diagnostics, more complex deep learning models do not necessarily lead to significantly better predictive performance. Therefore, in practical applications, it is crucial to balance model efficiency and prediction accuracy, prioritizing architectures that ensure reliable predictions with lower computational overhead and improved energy efficiency, thereby enhancing the feasibility and practical value of the overall system.

### 2.8 Trade‐off Analysis between Model Complexity and Predictive Accuracy

**FIGURE 7:**
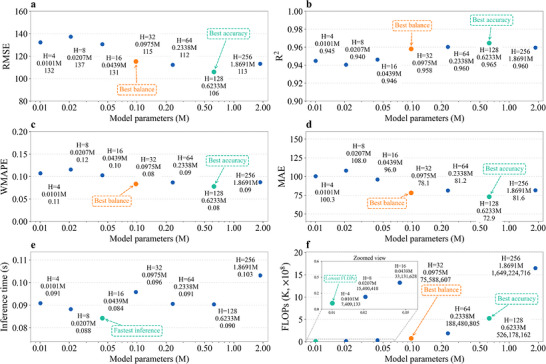
Comprehensive comparison of prediction performance and computational cost under different model sizes. Panels (a)–(d) show the variation in RMSE, R^2^, WMAPE, and MAE, respectively, with respect to the number of model parameters under different hidden dimensions. Panel (e) presents the inference time of each model size, and panel (f) shows the computational complexity measured by FLOPs. The model parameters are shown on a logarithmic x axis, and each point corresponds to one model configuration with a specific hidden dimension. The annotations identify representative configurations with the best balance, best accuracy, fastest inference, lowest FLOPs, or lowest computational cost. All inference experiments were conducted on a desktop workstation equipped with an NVIDIA GeForce RTX 4090 GPU with a thermal design power of 450 W.

In complex high‐dimensional battery diagnostic tasks, large‐scale models are often used to enhance representation capacity and improve prediction accuracy. However, in end‐to‐end battery lifetime prediction, model performance is governed not only by parameter scale, but also by temporal representation capability, inference efficiency, and computational complexity. A systematic assessment of the trade‐off between predictive accuracy and computational cost is therefore essential for practical deployment. The model‐parameter calculation and computational‐cost evaluation settings are provided in Supplementary Notes S3.8 and S3.9. To analyze the performance‐complexity relationship of FAST‐BatPro, a series of model configurations with different hidden dimensions were evaluated. The hidden dimension was progressively reduced from 256 to 4, decreasing the model size from the original 1.869 million parameters to lightweight configurations with fewer parameters (Tables S7–S13), and summarized together with performance and computational metrics in Table S14 (Supporting Information). The largest model maintained strong predictive performance, with an RMSE of 113 cycles, an R^2^ of 0.960 and an MAE of 81.6 cycles. However, it also had the highest computational cost, requiring approximately 1.65 billion FLOPs, 0.103 s inference time, and 0.0129 Wh energy consumption during testing. These results indicate that simply increasing model size does not yield the optimal balance between performance and complexity. Moderate model compression produced better overall predictive performance (shown in Figure 7 and Table S14, Supporting Information). When the hidden dimension was set to H = 128, the parameter count decreased to 0.623 M, and the model achieved the best predictive accuracy among the tested hidden‐dimension configurations, with an RMSE of 106 cycles, an MAE of 72.9 cycles, and an R^2^ of 0.965. Compared with the largest model, this configuration substantially reduced FLOPs and modestly reduced inference time and estimated energy consumption, suggesting that moderate compression can remove redundant model capacity while preserving prediction accuracy. Further compression revealed a more explicit trade‐off between performance and cost. The H = 32 model contained only 0.098 M parameters and reduced FLOPs to approximately 75.6 million, while still maintaining competitive predictive performance, with an RMSE of 115 cycles, an R^2^ of 0.958, and an MAE of 78.1 cycles. This configuration therefore provides a favorable balance between predictive performance and parameter/FLOP efficiency. By contrast, excessive compression weakened the model's representational capacity. Although H = 16, 8, and 4 further reduced parameter count and FLOPs, their prediction errors increased relative to the moderately compressed configurations, indicating that overly small model capacity is insufficient for representing complex degradation dynamics.

In addition, pruning experiments [67] were conducted to further evaluate whether model complexity could be reduced by removing module‐level structural redundancy, as described in Supplementary Note S4.0. The corresponding results are shown in Figures S15, S16 and Table S15 (Supporting Information). The pruning results show that the effect of pruning depends strongly on the module being pruned. In particular, FFN‐P100, a lightweight variant derived from the reference FAST‐BatPro architecture, reduced model parameters, FLOPs, and inference time while maintaining slightly improved predictive accuracy, with an RMSE of 106 cycles, an R^2^ of 0.965 and an MAE of 72.3 cycles. This result indicates compressible redundancy in the feed‐forward network and suggests that large nonlinear FFN mappings are not always required for this battery lifetime prediction task. In contrast, attention head pruning mainly improved inference efficiency but degraded prediction accuracy, whereas joint pruning further reduced runtime overhead but was more likely to compromise predictive performance. These results suggest that attention‐based temporal representation plays a more critical role in capturing degradation‐relevant information, while the feed‐forward network mainly contributes additional nonlinear capacity that can become redundant under this task setting.

Overall, these results reveal a clear nonlinear trade‐off among model size, prediction accuracy, and computational cost. Larger models do not necessarily provide higher accuracy, whereas excessive compression may reduce predictive performance. In the hidden dimension scaling experiments, H = 128 is more suitable when predictive accuracy is prioritized, whereas H = 32 provides a better balance between predictive performance and parameter/FLOP efficiency. The pruning results further indicate that efficient battery lifetime prediction should jointly optimize hidden dimension, feed‐forward network capacity, and attention head configuration to balance predictive accuracy, computational efficiency, energy consumption, and deployment feasibility.

## 3 Conclusion

This study proposes an efficient end‐to‐end learning framework for battery lifetime prediction. The framework is built on a customized Transformer architecture and integrates flash attention and probabilistic sparse attention to enhance the representation of local features, long‐range dependencies and temporally sparse information in battery degradation sequences. Although large‐scale models have strong capability for modelling nonlinear degradation trajectories, increasing model size usually leads to higher computational cost, energy consumption, and structural complexity. Therefore, analyzing the trade‐off between prediction accuracy and computational complexity from the perspective of model design is important for developing efficient battery lifetime prediction models. In battery lifetime prediction, model selection should not be guided only by the pursuit of the highest prediction accuracy. Task characteristics, data structure, computational cost, and the marginal benefit of accuracy improvement should be considered jointly. For battery data with clear degradation patterns and strong domain relevance, models with moderate complexity can often achieve a more reasonable balance between predictive performance and resource consumption. Based on this consideration, this study introduces a performance‐complexity analysis strategy that evaluates inference time, FLOPs, energy consumption, and accuracy improvement, providing a systematic basis for model structure selection and complexity control. The analysis shows that the performance gain from model complexity is strongly task‐dependent. Although large‐scale models generally improve representation capacity, compact deep learning models can also achieve high prediction accuracy when input features are strongly correlated with lifetime labels. For battery lifetime prediction, voltage, capacity, and incremental features during charging reflect key changes in degradation states. When input data contain sufficient degradation information, the model can learn the mapping between features and lifetime without relying on an excessively large parameter scale. This result is consistent with the bias‐variance trade‐off and indicates that physically meaningful input construction and domain‐relevant feature selection remain important even in end‐to‐end learning frameworks. The high prediction performance achieved using only partial charging data further suggests that battery degradation information has high information density within specific operating windows, providing a basis for efficient learning with compact models.

The model scaling experiments reveal a nonlinear relationship between model complexity and predictive performance. When the model size was reduced to approximately 33.4% of the original architecture, inference latency decreased by 12.6%, FLOPs by 68.1% and energy consumption by 12.6%, with only a minor effect on prediction performance. This suggests that moderate compression can remove redundant representation capacity and reduce computational overhead while preserving prediction accuracy. However, when the model was further compressed to a lower parameter scale, such as below 0.1M parameters, prediction accuracy began to decline, and computational efficiency did not consistently improve. In some cases, abnormal increases in computational load were observed. This may be related to insufficient capacity of extremely shallow structures to model sequential dependencies, changes in hardware execution efficiency, and characteristics of computational scheduling. Therefore, model compression cannot be treated as simple parameter reduction, and fewer parameters do not necessarily lead to better overall efficiency. The pruning experiments further support this conclusion from the perspective of module‐level structural redundancy. FFN pruning reduced parameter count, FLOPs, inference time, and energy consumption while maintaining favorable predictive performance, indicating compressible redundancy in the feed‐forward network of the Transformer. Moderate removal of redundant mappings did not markedly weaken the capacity of the model to represent battery degradation features. By contrast, attention head pruning improved inference efficiency but was more likely to affect multivariate degradation feature interactions and temporal dependency modelling. Joint pruning further reduced runtime overhead, but also increased the risk of accuracy degradation. These results indicate that model lightweighting should not rely solely on parameter reduction. Instead, the actual contribution of each module to predictive performance and computational cost should be identified to guide targeted structural optimization.

From an application perspective, these results provide guidance for selecting battery lifetime prediction models under different computational constraints. In resource‐limited scenarios, moderately complex models combined with input features containing clear degradation information can offer better overall performance than indiscriminate model expansion. Under offline analysis or high‐performance computing conditions, the use of more complex models should also be justified by substantial gains in prediction accuracy. Although this study does not rely on conventional handcrafted feature engineering, it uses domain‐informed representations, including SOC‐sub window‐normalized ΔQ and ΔV curves, enabling high prediction performance without full‐cycle data. This suggests that structural priors embedded in input construction can act as implicit regularization and improve generalization across experimental protocols and degradation modes. FAST‐BatPro also reflects an efficient strategy for sequence modelling. The hybrid attention design combines the computational efficiency of flash attention with the sparse modelling advantage of probabilistic sparse attention, reducing the cost of long‐sequence modelling while preserving prediction performance. This strategy may also provide methodological reference for other engineering time‐series tasks characterized by long‐range dependencies and temporal sparsity. The results further support a task‐aware model selection paradigm. Model complexity should be determined by task‐specific signal complexity, input feature informativeness, and the correlation between features and target variables. In structured physical systems, input features are often strongly correlated with prediction targets, allowing compact architectures to satisfy modelling requirements. The experiments show that further increasing hidden dimensions and parameter count did not improve prediction accuracy and, in some cases, degraded performance. This may be associated with overparameterization relative to task complexity, training instability, and reduced hardware parallelization efficiency. Therefore, in multiscale and multiphysics time‐series modelling tasks such as battery lifetime prediction, the principle of “larger is better” should be reconsidered. A more appropriate strategy is to adaptively determine model configurations according to input data structure, degradation feature complexity and computational cost constraints, thereby balancing representation capacity, computational efficiency and prediction stability. Future work could explore meta‐learning strategies and task‐aware scaling laws by incorporating signal‐to‐noise ratio, feature dimensionality, and degradation pattern complexity into model capacity estimation. Cross‐task knowledge transfer may further reduce training costs on new datasets and improve adaptability to new operating conditions and degradation modes.

## 4 Methods

### 4.1 End‐to‐end Deep Learning Pipeline

A deep learning method built on the FAST‐BatPro hybrid fusion model was developed for rapid battery RUL prediction. The model is trained by updating layer parameters using multi‐cycle data from the training cells within an end‐to‐end learning framework. The trained model is then validated using a test set containing various charge and discharge protocols. Unlike traditional models, the proposed framework does not require handcrafted features derived from complete charge‐discharge trajectories. Instead, the model predicts RUL using partial charging data from the early cycles under different test protocols.

### 4.2 Domain‐Informed Input Feature Construction

In previous research, one study demonstrated that the high‐performance feature, the logarithmic variance of △*Q*_100‐10_ (*V*) (representing the difference in discharge capacity curves between the 100th and 10th cycles), shows a strong correlation with battery cycle life [33]. Another study indicates that △*V_b‐a_* (*Q*) (the difference in charging voltage curves between the *b*‐th and *a*‐th cycles) also contains high‐performance features related to battery cycle life [68]. Therefore, we selected charging voltage curves, charging capacity curves, and their cycle‐wise differences relative to the reference cycle as input features for the model (Figure S17, Supporting Information), where the reference cycle was defined as the 10th cycle for LFP cells, the 6th cycle for NCA cells, and the 3rd cycle for NMC cells.

### 4.3 Fixed‐Length Curve Interpolation

To avoid discrepancies in input feature dimensions and improve pattern learning, the voltage and capacity curves measured from 70% SOC to 4.2 V for NCA and NMC cells and from 80% SOC to 3.6 V for LFP cells were fitted to a time‐dependent function. The number of available data points gradually decreased over aging cycles. These curves were then linearly interpolated to a fixed length of 100. All four feature curves were processed in the same manner, fitted as time functions and linearly interpolated to a uniform length of 100, ensuring consistency in the model input and simplifying model operation. Consequently, each cycle generated 1×4×100 feature data points, facilitating more efficient model processing and management.

(2) $$y=y_{0}+\frac{\left(y_{1}-y_{0}\right)\times \left(x-x_{0}\right)}{x_{1}-x_{0}}$$

where *x*_0_ and *x*_1_ represent the time data within the range from 80% SOC to the point where the voltage reaches 3.6 V for LFP, and from 70% SOC to the point where the voltage reaches 4.2 V for NCA/NMC, while *y*_0_ and *y*_1_ correspond to the voltage or capacity data for this charging range.

### 4.4 Sliding‐Window Sample Construction

The input sequences were segmented using a fixed‐length sliding window. The input length and step size were set to 40 cycles and 1 cycle for dataset 1, 100 cycles and 5 cycles for dataset 2, and 10 cycles and 1 cycle for datasets 3 and 4, respectively, to generate annotated input‐output pairs. This strategy provides the model with a comprehensive view of the dataset, allowing it to identify short‐term dependencies and patterns. Furthermore, a sampling interval of two cycles was adopted to balance data richness preservation with reduced computational burden. This approach effectively enriches the dataset, with each input sample generating 1×4×100×20 data points for LFP and 1×4×100×10 for NCA/NMC, corresponding to 1 sample, 4 feature curves, 100 sampling points, and 20 or 10 cycles, respectively.

### 4.5 Min–Max Normalization

Feature vector normalization effectively addresses prediction bias caused by inconsistencies in the scales of different features, ensuring that each feature contributes more equally to the model output, thereby improving prediction accuracy. Additionally, normalization enhances model training efficiency by accelerating the convergence process and facilitating more effective model development. Min–max normalization scales data to a range of [0, 1] or [−1, 1], ensuring that all feature data are on the same scale. This process eliminates dimensional inconsistencies and improves numerical stability in computations.

(3) $$x^{\prime }=\frac{x-\min(x)}{\max(x)-\min(x)}$$

where *x*′ represents the normalized value, *x* represents the feature value, max (*x*) represents the maximum feature value, min (*x*) represents the minimum feature value.

### 4.6 Attention‐Aware Modeling and Electrochemical Interpretation

The proposed FAST‐BatPro model adopts a Transformer encoder–decoder architecture to enhance global feature extraction [69]. Specifically, the input data first undergoes embedding and positional encoding, effectively integrating the information from the four feature sequences during charging at each time step. The encoder computes attention weights between each time step and all other time steps in the input sequence, enabling the extraction of global features. These features are then transformed non‐linearly through a feed‐forward neural network, allowing for the extraction of higher‐level abstract features. The encoder ultimately generates a feature map containing the characteristics of all time steps. This feature map is used by the decoder to generate predictions of the RUL.

To clarify how the retained high‐SOC charging information affects attention‐aware lifetime prediction and its electrochemical interpretation, the high‐SOC charging segment from approximately 80% SOC to the 3.6 V cut‐off voltage was used as the baseline input window. Within this window, 100%, 90%, 80%, 70%, 60%, and 50% of the charging information were retained as model inputs. These retention ratios represent the relative amount of information preserved within the high‐SOC charging window, rather than absolute SOC intervals from 0% SOC to the corresponding SOC levels. Therefore, this experiment analyzes how the retained information within late‐stage high‐SOC charging segments contributes to lifetime prediction. From an electrochemical perspective, the measured voltage response of LFP‐graphite batteries during high‐SOC charging can reflect coupled aging‐related processes, including cathode delithiation, lithium intercalation into the graphite anode, SEI evolution, internal resistance growth, and concentration polarization [70, 71, 72, 73]. During charging, lithium ions are extracted from the LiFePO_4_ cathode and inserted into the graphite anode layers. The externally measured voltage capacity curve reflects the combined effects of electrode reactions, mass transport, and polarization. Previous studies have shown that the LiFePO_4_ cathode exhibits pronounced two phase transition behavior, usually appearing as a flat voltage plateau, whereas lithium intercalation in the graphite anode is accompanied by staged structural evolution [74]. In addition, interfacial layer regulation can suppress active lithium loss, reduce polarization and stabilize the electrode interface, thereby improving capacity retention under high‐rate conditions [75]. These mechanisms indicate that the terminal voltage response in the high SOC region is closely related to capacity fade, polarization growth, and interfacial degradation. In addition, the IC curve can serve as an intermediate representation linking data‐driven models with electrochemical degradation mechanisms. Its peak height, peak voltage, and peak area respectively reflect the local capacity response intensity, reaction potential shift, and available capacity within a specific voltage range [76, 77]. Compared with direct observation of voltage capacity curves, IC curves amplify subtle variations in plateau and phase transition regions, and are therefore widely used to identify degradation features associated with capacity fade, polarization growth and changes in reaction kinetics. Related studies have also shown that DQV or IC type differential features can improve observability in the flat OCV SOC region of LFP batteries and enhance SOC or health state estimation [78]. Therefore, IC curves provide an electrochemically meaningful basis for interpreting the influence of different input charging information on lifetime prediction performance.

The experimental results further support the above interpretation (Figures S18 and S19, Supporting Information). As the retained proportion of the charging window from approximately 80% SOC to the 3.6 V cut‐off voltage decreases from 100% to 50%, the high voltage end of the IC curve is progressively truncated. The curve morphology changes from a complete late stage charging response to a profile that mainly retains information from the early part of the high SOC window. Correspondingly, the average IC peak area decreases from 0.140 to 0.097 Ah, indicating a gradual reduction in capacity contribution and polarization evolution near the charge termination stage. The decrease in peak area suggests a weaker effective capacity response observable by the model under shorter retained input proportions, whereas changes in peak voltage and peak shape further reflect differences in reaction potential and polarization information across retained windows. Thus, reducing the retained input proportion decreases the amount of input data and weakens the electrochemical features available for aging state identification. In addition, the prediction results are consistent with the IC curve analysis (Figure S18 and Table S16, Supporting Information). As the retained input proportion decreases, RMSE, WMAPE, and MAE generally increase, whereas R^2^ decreases, indicating reduced prediction accuracy and stability. This result suggests that plateau tail information, polarization response, and remaining capacity contribution near the 3.6 V cut off region are not the only effective information sources, but they provide important complementary information for lifetime prediction. Therefore, changes in model performance are governed not only by input length, but also by whether the input region covers key electrochemically sensitive degradation features within the high SOC charging window.

Based on this analysis, attention aware means more than the presence of an attention mechanism in the model architecture. It indicates that the model can adaptively emphasize degradation related information regions in historical cycle sequences and high SOC charging trajectories. Because battery aging is cumulative and path dependent, different historical cycles, charging segments, and voltage capacity regions contribute unequally to lifetime prediction. The attention mechanism can capture these nonuniform contributions and guide the model toward degradation sensitive features, including capacity fade, polarization evolution and IC peak variation. Therefore, the attention‐aware analysis suggests that the model is sensitive to degradation‐relevant voltage‐capacity regions, providing a physically informed interpretation of the prediction results without implying a direct causal relationship between attention weights and electrochemical mechanisms.

### 4.7 Flash Self‐Attention Mechanism

Flash attention‐2 employs a tiling technique during the attention computation process, optimizing memory access strategies and reducing dependence on global memory, significantly lowering the overhead of memory I/O operations [79]. Specifically, the input key (*K*), query (*Q*), and value (*V*) matrices are divided into smaller blocks based on block size. The *K* and *V* matrices are loaded and stored in shared memory in one go, while the *Q* matrix is loaded in four sequential parts. After loading each *Q* block, it is multiplied with the cached *K* and *V* blocks to compute a partial attention score matrix *S*. Subsequently, a SoftMax operation is performed immediately on the result of each *Q* block, followed by multiplying it with the corresponding *V* block to generate a partial output matrix *O*. By integrating computation and storage operations, unnecessary memory read and write operations are minimized, reducing the burden on global memory and the need for intermediate result storage, thus ensuring overall computational efficiency and speed (Figure S20¸ Supporting Information). Unlike vanilla attention mechanisms, flash attention‐2 optimizes memory usage by avoiding the explicit storage of the full attention score matrix and instead employing block‐wise computation. This approach reduces the memory complexity for time‐series data of length *L* from *O*(*L* ^2^) to a value closer to *O*(*L*), thereby significantly decreasing memory requirements, particularly when dealing with long sequences. It leverages the multi‐tier memory architecture of the underlying hardware, including shared memory and registers, which serve as high‐throughput storage areas. By storing key computational data in shared memory and registers, global memory reads are significantly reduced, thereby improving computational speed. In addition, with the model architecture and data split kept consistent, we assessed the sensitivity of predictive performance to training configurations by varying only the batch size, learning rate, and attention dropout rate in the Flash Attention mechanism (Figure S21) in Supplementary Note 4.2. Batch sizes of 128, 256, 512, and 768 were examined with corresponding learning rates of 1 × 10^−^^4^, 1.5 × 10^−^^4^, 2 × 10^−^^4^, and 2.5 × 10^−^^4^, respectively, while the attention dropout rate was varied from 0.2 to 0.6. In summary, flash attention enhances computational efficiency through block‐wise computation and optimizations that reduce intermediate result storage.

### 4.8 Masked Probabilistic Sparse Self‐Attention Mechanism

In the vanilla self‐attention mechanism, each *Q*‐*K* pair undergoes a dot product calculation, resulting in a computational complexity of *O*(*L* ^2^). This fully connected computation becomes costly when processing long sequences, particularly in real‐world applications, where many query‐key pairs contribute minimally, following a “long‐tail” distribution. Sparse self‐attention mitigates this issue by limiting the number of tokens each token attends to, either through predefined patterns or by learning which tokens to prioritize, thus reducing the computational cost to linear or sub‐quadratic complexity. The probabilistic sparse attention mechanism is integrated into the decoder architecture to reduce both computational and memory overhead when processing long sequences. The core idea behind the probsparse self‐attention mechanism is to focus computation only on the *Q*‐*K* pairs that have the most significant impact on the output, while disregarding fewer influential pairs. This approach reduces computational complexity from *O*(*L* ^2^) to *O*(*L*log *L*) [80]. By selectively attending to the most relevant tokens, the mechanism preserves model performance while significantly lowering computational overhead and memory usage. Applying this sparse self‐attention mechanism enables the capture of key attention weights responsible for battery aging across different time scales.

The input sequence *X* ∈ *R* ^*L* × *d*^, with *L* representing its length and *d* its feature dimension, is mapped to the query, key, and value vectors: *Q*, *K*, and *V*.

(4) $$\left\{\begin{array}{c} Q=XW_{Q}\\ K=XW_{K}\\ V=XW_{V} \end{array}\right.$$

where the matrices *W_Q_*, *W_K_*, and *W_V_* are learnable projections that map the input into query, key, and value vectors, respectively.

Next, simplified attention score calculations between a randomly selected subset of *K* and all *Q*, the most active *Q* elements are identified to obtain the key attention scores.

(5) $$Score\left(Q,K\right)=QK^{T}$$

After this sparse selection process, the SoftMax function is applied to the attention score matrix, resulting in sparse attention weights. The attention weights from the four heads are then concatenated and transformed through a linear transformation to produce the final attention output.

(6) α=softmax(Score(Q,K))

(7) $$MultiHead\left(Q,K,V\right)=Concat\left(head_{1},\text{…},head_{4}\right)W^{0}$$

(8) $$where\;head_{i}=\alpha V$$

In the sparse attention mechanism, the sparse attention score matrix not only reduces the computational burden through selective sparsity but also ensures causality in autoregressive tasks by applying an upper triangular mask. The mask matrix prevents access to future information in the sparse attention score matrix, allowing the model to focus only on the information required for the current task. This approach significantly improves the efficiency and accuracy of the attention mechanism. Flash attention achieves substantial computational and memory efficiency through block‐wise attention computation. In FAST‐BatPro, the hybrid attention mechanism refers to the task‐specific integration of flash attention and masked probabilistic sparse attention within an encoder‐decoder framework for battery lifetime prediction. In our implementation, flash attention is primarily used for efficient global attention modeling, whereas causal temporal dependency is handled through the masked probabilistic sparse self‐attention module. Specifically, flash self‐attention in the encoder captures global degradation dependencies with reduced memory overhead, masked probabilistic sparse self‐attention in the decoder supports causal and selective temporal interaction modeling, and flash cross‐attention connects decoder states with encoder‐derived degradation representations. This hybrid attention design balances computational efficiency with autoregressive temporal modeling capability. Finally, this information is integrated through fully connected layers to produce accurate prediction results.

### 4.9 Efficient Training and Inference Strategy

During training and inference, the hyperparameters and their configurations are summarized in Table S17 and described in Supplementary Note 4.3. At the same time, FAST‐BatPro reduces redundant computation at three levels: data organization, training configuration, and the forward computational pathway, thereby improving overall runtime efficiency. At the data organization stage, interval sampling with a stride of 10 is applied to the training and validation sets to reduce the strong overlap between adjacent sliding windows and decrease the redundant training cost caused by repeated charging segments. During pretraining, a large batch size is used to better exploit GPU parallelism, improve training throughput, and reduce the overhead from frequent parameter updates the details related to weight optimization are provided in Supplementary Note S4.4. In terms of the training objective, RUL prediction error is used as the main optimization target, and the capacity auxiliary loss weight is set to zero, allowing pretraining to focus on the mapping between lifetime labels and charging curve features. During source‐domain pretraining, the maximum number of epochs was set to 10,000, with a learning rate of 2 × 10^−^^4^, and convergence was monitored using the training loss. During target‐domain training, the dataset was divided into training and validation sets, and early stopping was applied based on validation performance with a patience of 1200 epochs.

During forward propagation, model efficiency mainly arises from compact feature representation and efficient attention computation. Raw charging segments are first mapped into a unified hidden space through the embedding layer, forming token representations suitable for encoder and decoder processing. This avoids direct computation in the highly redundant raw input space. In the encoder, multi‐head flash self‐attention models global dependencies among cycling stages, charging segments, and degradation features. Compared with conventional self‐attention, flash attention optimizes attention matrix computation and memory access, reducing the storage and read‐write overhead of intermediate matrices and improving training and inference efficiency. Meanwhile, the one‐dimensional convolutional layer extracts short‐range temporal variations through local connectivity and weight sharing, enabling the model to capture local degradation patterns with lower computational cost and reducing its dependence on full global attention computation. The decoder further filters lifetime‐relevant information from the degradation representations produced by the encoder. Masked probabilistic sparse self‐attention prioritizes representative attention interactions according to the information contribution of tokens, thereby reducing redundant computation among low‐contribution tokens. Flash cross‐attention then associates decoder states with encoder‐derived degradation representations, allowing the model to focus on information more relevant to RUL prediction instead of computing indiscriminately over all input features. In this way, FAST‐BatPro forms an efficient computational pathway that integrates compact embedding, global dependency modelling, local feature enhancement, and relevance‐based information selection during training and inference.

Complexity statistics and supplementary experiments further validate the rationale of this efficient structure. The hidden dimension directly determines the scale of matrix operations in Q, K, and V projections, feed‐forward networks, and fully connected layers. Hidden‐dimension compression experiments show that the model maintains stable predictive performance after an appropriate reduction in hidden dimension, indicating redundancy in the original hidden feature channels and suggesting that battery lifetime prediction can be performed in a more compact feature space. Pruning experiments further identify structural redundancy at the module level. FFN pruning removes redundant nonlinear mappings in the feed‐forward network, thereby reducing parameter count and FLOPs. Attention head pruning simplifies the attention computation pathway and improves practical inference efficiency, whereas joint pruning evaluates the complexity gains and accuracy losses when multiple modules are compressed simultaneously. Overall, the efficient operation of FAST‐BatPro is supported not by a single compression strategy, but by the combined effects of training data redundancy reduction, batch‐based parallel training, compact feature representation, efficient attention computation, hidden‐dimension control, and module‐level pruning. This design reduces computational cost while preserving the capacity of the model to represent temporal degradation features, providing a structural basis for efficient battery lifetime prediction.

## Conflicts of Interest

The authors declare no conflicts of interest.

## Code Availability

Code is available at https://github.com/jyzhao2024/Task‐Aware_Transformer_Scaling_for_Resource‐Efficient_Battery_Lifetime_Prediction.git and additional information upon request to the lead contact.

## Supporting information




**Supporting File**: advs76597‐sup‐0001‐SuppMat.pdf.

## Data Availability

The four datasets used in this study for battery degradation modeling and lifetime prediction are openly available and can be accessed as follows: the LFP datasets at https://doi.org/10.17632/nsc7hnsg4s.2 and https://data.matr.io/1/projects/5c48dd2bc625d700019f3204; the NCA dataset at https://doi.org/10.5281/zenodo.6379165; and the NMC dataset at https://www.batteryarchive.org/list.html.

## References

[1] D. Castelvecchi , “Electric Cars and Batteries: How Will the World Produce Enough?,” Nature 596, no. 7872 (2021): 336–339. 34404944 10.1038/d41586-021-02222-1

[2] C. Y. Wang , T. Liu , X. G. Yang , et al., “Fast Charging of Energy‐dense Lithium‐ion Batteries,” Nature 611, no. 7936 (2022): 485–490. 36224388 10.1038/s41586-022-05281-0

[3] M. R. Palacín and A. de Guibert , “Why Do Batteries Fail?,” Science 351, no. 6273 (2016): 1253292. 26912708 10.1126/science.1253292

[4] S. J. Harris and M. M. Noack , “Statistical and Machine Learning‐based Durability‐testing Strategies for Energy Storage,” Joule 7, no. 5 (2023): 920–934.

[5] M. Berecibar , “Machine‐learning Techniques Used to Accurately Predict Battery Life,” Nature 568, no. 7752 (2019): 325–326. 30980036 10.1038/d41586-019-01138-1

[6] M. F. Ng , J. Zhao , Q. Yan , G. J. Conduit , and Z. W. Seh , “Predicting the state of Charge and Health of Batteries Using Data‐driven Machine Learning,” Nature Machine Intelligence 2, no. 3 (2020): 161–170.

[7] D. Roman , S. Saxena , V. Robu , M. Pecht , and D. Flynn , “Machine Learning Pipeline for Battery state‐of‐health Estimation,” Nature Machine Intelligence 3, no. 5 (2021): 447–456.

[8] A. Geslin , B. Van Vlijmen , X. Cui , et al., “Selecting the Appropriate Features in Battery Lifetime Predictions,” Joule 7, no. 9 (2023): 1956–1965.

[9] A. Aitio and D. A. Howey , “Predicting Battery End of Life from Solar off‐grid System Field Data Using Machine Learning,” Joule 5, no. 12 (2021): 3204–3220.

[10] Y. Che , X. Hu , and R. Teodorescu , “Opportunities for Battery Aging Mode Diagnosis of Renewable Energy Storage,” Joule 7, no. 7 (2023): 1405–1407.

[11] X. Hu , L. Xu , X. Lin , and M. Pecht , “Battery Lifetime Prognostics,” Joule 4, no. 2 (2020): 310–346.

[12] A. Wang , S. Kadam , H. Li , S. Shi , and Y. Qi , “Review on Modeling of the Anode Solid Electrolyte Interphase (SEI) for Lithium‐ion Batteries,” NPJ Computational materials 4, no. 1 (2018): 15.

[13] Q. Zhao , S. Stalin , and L. A. Archer , “Stabilizing Metal Battery Anodes through the Design of Solid Electrolyte Interphases,” Joule 5, no. 5 (2021): 1119–1142.

[14] X. Lin , K. Khosravinia , X. Hu , J. Li , and W. Lu , “Lithium Plating Mechanism, Detection, and Mitigation in Lithium‐ion Batteries,” Progress in Energy and Combustion Science 87 (2021): 100953.

[15] W. Huang , Y. Ye , H. Chen , et al., “Onboard Early Detection and Mitigation of Lithium Plating in Fast‐charging Batteries,” Nature communications 13, no. 1 (2022): 7091. 10.1038/s41467-022-33486-4 PMC9675798 36402759

[16] Y. F. Liu , K. Han , D. N. Peng , et al., “Layered Oxide Cathodes for Sodium‐Ion Batteries: from Air Stability, Interface Chemistry to Phase Transition,” InfoMat 5, no. 6 (2023): e12422.

[17] Y. Wang , Z. Feng , P. Cui , et al., “Pillar‐beam Structures Prevent Layered Cathode Materials from Destructive Phase Transitions,” Nature Communications 12, no. 1 (2021): 13. 10.1038/s41467-020-20169-1 PMC7782780 33397895

[18] Z. Chen , D. L. Danilov , R. A. Eichel , and P. H. Notten , “Porous Electrode Modeling and Its Applications to Li‐Ion Batteries,” Advanced Energy Materials 12, no. 32 (2022): 2201506.

[19] C. Fan , K. O'Regan , L. Li , M. D. Higgins , E. Kendrick , and W. D. Widanage , “Data‐driven Identification of Lithium‐ion Batteries: a Nonlinear Equivalent Circuit Model with Diffusion Dynamics,” Applied Energy 321 (2022): 119336.

[20] A. Berrueta , A. Urtasun , A. Ursúa , and P. Sanchis , “A Comprehensive Model for Lithium‐ion Batteries: from the Physical Principles to an Electrical Model,” Energy 144 (2018): 286–300.

[21] C. Fan , K. Liu , Y. Ren , and Q. Peng , “Characterization and Identification towards Dynamic‐based Electrical Modeling of Lithium‐ion Batteries,” Journal of Energy Chemistry 92 (2024): 738–758.

[22] Y. Wu , L. Xie , H. Ming , et al., “An Empirical Model for the Design of Batteries with High Energy Density,” ACS Energy Letters 5, no. 3 (2020): 807–816.

[23] M. Aykol , P. Herring , and A. Anapolsky , “Machine Learning for Continuous Innovation in Battery Technologies,” Nature Reviews Materials 5, no. 10 (2020): 725–727.

[24] Y. Li , K. Liu , A. M. Foley , et al., “Data‐driven Health Estimation and Lifetime Prediction of Lithium‐ion Batteries: a Review,” Renewable and sustainable energy reviews 113 (2019): 109254.

[25] L. Xu , F. Wu , R. Chen , and L. Li , “Data‐driven‐aided Strategies in Battery Lifecycle Management: Prediction, Monitoring, and Optimization,” Energy Storage Materials 59 (2023): 102785.

[26] S. Li , H. He , C. Su , and P. Zhao , “Data Driven Battery Modeling and Management Method with Aging Phenomenon Considered,” Applied Energy 275 (2020): 115340.

[27] S. Khaleghi , M. S. Hosen , D. Karimi , et al., “Developing an Online Data‐driven Approach for Prognostics and Health Management of Lithium‐ion Batteries,” Applied Energy 308 (2022): 118348.

[28] X. Sui , S. He , J. Meng , R. Teodorescu , and D. I. Stroe , “Fuzzy Entropy‐based state of Health Estimation for Li‐ion Batteries,” IEEE Journal of Emerging and Selected Topics in Power Electronics 9, no. 4 (2020): 5125–5137.

[29] X. Lai , Y. Yao , X. Tang , et al., “Voltage Profile Reconstruction and state of Health Estimation for Lithium‐ion Batteries under Dynamic Working Conditions,” Energy 282 (2023): 128971.

[30] X. Li , M. Lyu , K. Li , X. Gao , C. Liu , and Z. Zhang , “Lithium‐ion Battery state of Health Estimation Based on Multi‐source Health Indicators Extraction and Sparse Bayesian Learning,” Energy 282 (2023): 128445.

[31] J. Shen , W. Ma , X. Shu , S. Shen , Z. Chen , and Y. Liu , “Accurate state of Health Estimation for Lithium‐ion Batteries under Random Charging Scenarios,” Energy 279 (2023): 128092.

[32] C. She , Y. Li , C. Zou , T. Wik , Z. Wang , and F. Sun , “Offline and Online Blended Machine Learning for Lithium‐ion Battery Health state Estimation,” IEEE Transactions on Transportation Electrification 8, no. 2 (2021): 1604–1618.

[33] K. A. Severson , P. M. Attia , N. Jin , et al., “Data‐driven Prediction of Battery Cycle Life before Capacity Degradation,” Nature Energy 4, no. 5 (2019): 383–391.

[34] P. M. Attia , A. Grover , N. Jin , et al., “Closed‐loop Optimization of Fast‐charging Protocols for Batteries with Machine Learning,” Nature 578, no. 7795 (2020): 397–402. 32076218 10.1038/s41586-020-1994-5

[35] J. Pan , B. Sun , Z. Wu , et al., “Probabilistic Remaining Useful Life Prediction without Lifetime Labels: a Bayesian Deep Learning and Stochastic Process Fusion Method,” Reliability Engineering & System Safety 250 (2024): 110313.

[36] T. Wang , Z. Liu , M. Liao , N. Mrad , and G. Lu , “Probabilistic Analysis for Remaining Useful Life Prediction and Reliability Assessment,” IEEE Transactions on Reliability 71, no. 3 (2020): 1207–1218.

[37] A. Bracale , P. De Falco , L. P. Di Noia , and R. Rizzo , “Probabilistic state of Health and Remaining Useful Life Prediction for Li‐ion Batteries,” IEEE Transactions on Industry Applications 59, no. 1 (2022): 578–590.

[38] D. Liu , W. Xie , H. Liao , and Y. Peng , “An Integrated Probabilistic Approach to Lithium‐ion Battery Remaining Useful Life Estimation,” IEEE Transactions on Instrumentation and Measurement 64, no. 3 (2014): 660–670.

[39] M. Ahwiadi and W. Wang , “An Enhanced Particle Filter Technology for Battery System state Estimation and RUL Prediction,” Measurement 191 (2022): 110817.

[40] C. H. B. Apribowo , S. P. Hadi , F. D. Wijaya , and M. I. B. Setyonegoro , “Early Prediction of Battery Degradation in Grid‐scale Battery Energy Storage System Using Extreme Gradient Boosting Algorithm,” Results in Engineering 21 (2024): 101709.

[41] A. Rai and J. Liu , “A Novel Feature Adaptive Meta‐model for Efficient Remaining Useful Life Prediction of Lithium‐ion Batteries,” Journal of Energy Storage 114 (2025): 115715.

[42] H. Feng and D. Xue , “Parallel‐branch Enhanced ShuffleNet with Dual‐physics Constraints for Lithium‐ion Battery RUL Prediction,” Journal of Energy Storage 118 (2025): 116210.

[43] H. Madugula , A. Gorityala , S. Singh , V. R. Muppani , and S. Radhika , “A lotus‐optimized Radial Basis Function Framework for Explainable and Energy‐efficient Battery Health Prediction in Electric Vehicles,” Energy 347 (2026): 140419.

[44] Z. Lv and J. Zhao , “Resource‐efficient Artificial Intelligence for Battery Capacity Estimation Using Convolutional FlashAttention Fusion Networks,” ETransportation 23 (2025): 100383.

[45] C. Yin , B. Fei , J. Yao , et al., “Enhancing Degradation Trend Prediction of Lithium‐ion Battery Capacity in Complex Aging Scenarios: a Bayesian‐optimized Hybrid Architecture Combining Local and Global Feature Learning,” Journal of King Saud University Computer and Information Sciences 38, no. 2 (2026): 75.

[46] X. Liao , S. Chen , P. Wen , and S. Zhao , “Remaining Useful Life with Self‐attention Assisted Physics‐informed Neural Network,” Advanced Engineering Informatics 58 (2023): 102195.

[47] L. Ren , H. Wang , and G. Huang , “DLformer: a Dynamic Length Transformer‐based Network for Efficient Feature Representation in Remaining Useful Life Prediction,” IEEE transactions on neural networks and learning systems 35, no. 5 (2023): 5942–5952. 10.1109/TNNLS.2023.3257038 37030842

[48] V. Sulzer , P. Mohtat , A. Aitio , et al., “The Challenge and Opportunity of Battery Lifetime Prediction from Field Data,” Joule 5, no. 8 (2021): 1934–1955.

[49] Y. LeCun , Y. Bengio , and G. Hinton , “Deep Learning,” Nature 521, no. 7553 (2015): 436–444. 26017442 10.1038/nature14539

[50] J. Tian , R. Xiong , W. Shen , J. Lu , and X. G. Yang , “Deep Neural Network Battery Charging Curve Prediction Using 30 Points Collected in 10 Min,” Joule 5, no. 6 (2021): 1521–1534.

[51] H. Liu , C. Li , X. Hu , et al., “Multi‐modal Framework for Battery state of Health Evaluation Using Open‐source Electric Vehicle Data,” Nature Communications 16, no. 1 (2025): 1137. 10.1038/s41467-025-56485-7 PMC11779878 39880811

[52] J. Lu , R. Xiong , J. Tian , C. Wang , and F. Sun , “Deep Learning to Estimate Lithium‐ion Battery state of Health without Additional Degradation Experiments,” Nature Communications 14, no. 1 (2023): 2760. 10.1038/s41467-023-38458-w PMC10183024 37179411

[53] H. Zhang , Y. Li , S. Zheng , et al., “Battery Lifetime Prediction across Diverse Ageing Conditions with Inter‐cell Deep Learning,” Nature Machine Intelligence 7, no. 2 (2025): 270–277.

[54] Z. Fei , Z. Zhang , F. Yang , and K. L. Tsui , “Deep Learning Powered Rapid Lifetime Classification of Lithium‐ion Batteries,” ETransportation 18 (2023): 100286.

[55] Z. Fei , Z. Zhang , F. Yang , and K. L. Tsui , “A Deep Attention‐assisted and Memory‐augmented Temporal Convolutional Network Based Model for Rapid Lithium‐ion Battery Remaining Useful Life Predictions with Limited Data,” Journal of Energy Storage 62 (2023): 106903.

[56] P. Ding , X. Liu , H. Li , et al., “c,” Renewable and Sustainable Energy Reviews 148 (2021): 111287.

[57] Z. Wang , N. Liu , C. Chen , and Y. Guo , “Adaptive Self‐attention LSTM for RUL Prediction of Lithium‐ion Batteries,” Information Sciences 635 (2023): 398–413.

[58] W. Zhao , W. Ding , S. Zhang , and Z. Zhang , “Enhancing Lithium‐ion Battery Lifespan Early Prediction Using a Multi‐branch Vision Transformer Model,” Energy 302 (2024): 131816.

[59] N. H. Paulson , J. Kubal , and S. J. Babinec , “Multivariate Prognosis of Battery Advanced state of Health via Transformers,” Cell Reports Physical Science 5, no. 5 (2024): 101928.

[60] J. Zhao and Z. Wang , “Specialized Convolutional Transformer Networks for Estimating Battery Health via Transfer Learning,” Energy Storage Materials 71 (2024): 103668.

[61] B. Zhao , W. Zhang , Y. Zhang , C. Zhang , C. Zhang , and J. Zhang , “Research on the Remaining Useful Life Prediction Method for Lithium‐ion Batteries by Fusion of Feature Engineering and Deep Learning,” Applied Energy 358 (2024): 122325.

[62] J. Zhao , X. Feng , J. Wang , Y. Lian , M. Ouyang , and A. F. Burke , “Battery Fault Diagnosis and Failure Prognosis for Electric Vehicles Using Spatio‐temporal Transformer Networks,” Applied Energy 352 (2023): 121949.

[63] M. Li , C. Dong , B. Xiong , et al., “STTEWS: a Sequential‐transformer Thermal Early Warning System for Lithium‐ion Battery Safety,” Applied Energy 328 (2022): 119965.

[64] G. Ma , S. Xu , B. Jiang , et al., “Real‐time Personalized Health Status Prediction of Lithium‐ion Batteries Using Deep Transfer Learning,” Energy & Environmental Science 15, no. 10 (2022): 4083–4094.

[65] J. Zhu , Y. Wang , Y. Huang , et al., “Data‐driven Capacity Estimation of Commercial Lithium‐ion Batteries from Voltage Relaxation,” Nature communications 13, no. 1 (2022): 2261. 10.1038/s41467-022-29837-w PMC9046220 35477711

[66] Y. Preger , H. M. Barkholtz , A. Fresquez , et al., “Degradation of Commercial Lithium‐ion Cells as a Function of Chemistry and Cycling Conditions,” Journal of The Electrochemical Society 167, no. 12 (2020): 120532.

[67] R. Reed , “Pruning Algorithms‐a Survey,” IEEE transactions on Neural Networks 4, no. 5 (1993): 740–747. 18276504 10.1109/72.248452

[68] B. Jiang , W. E. Gent , F. Mohr , et al., “Bayesian Learning for Rapid Prediction of Lithium‐ion Battery‐cycling Protocols,” Joule 5, no. 12 (2021): 3187–3203.

[69] A. Vaswani , N. Shazeer , N. Parmar , et al., “Attention Is All You Need,” Advances in Neural Information Processing Systems 30 (2017): 5998–6008.

[70] D. Gupta and G. M. Koenig Jr , “Analysis of Chemical and Electrochemical Lithiation/Delithiation of a Lithium‐ion Cathode Material,” Journal of the Electrochemical Society 167, no. 2 (2020): 020537.

[71] Y. Lin , W. Hu , M. Ding , et al., “Unveiling the Three Stages of Li Plating and Dynamic Evolution Processes in Pouch C/LiFePO_4_ Batteries,” Advanced Energy Materials 14, no. 36 (2024): 2400894.

[72] W. Liang , X. Zhou , B. Zhang , et al., “The Versatile Establishment of Charge Storage in Polymer Solid Electrolyte with Enhanced Charge Transfer for LiF‐Rich SEI Generation in Lithium Metal Batteries,” Angewandte Chemie International Edition 63, no. 18 (2024): e202320149. 38430213 10.1002/anie.202320149

[73] Z. Y. Guo , Z. Y. Ji , H. Y. Chen , et al., “Effect of Impurity Ions in the Electrosorption Lithium Extraction Process: Generation and Restriction of “Selective Concentration Polarization”,” ACS Sustainable Chemistry & Engineering 8, no. 31 (2020): 11834–11844.

[74] R. Malik , A. Abdellahi , and G. Ceder , “A Critical Review of the Li Insertion Mechanisms in LiFePO_4_ Electrodes,” Journal of the electrochemical society 160, no. 5 (2013): A3179–A3197.

[75] R. Tang , J. Dong , C. Wang , et al., “Rate‐Dependent Failure Behavior Regulation of LiFePO_4_ Cathode via Functional Interface Engineering,” Advanced Functional Materials 35, no. 22 (2025): 2421284.

[76] X. Li , J. Jiang , D. Chen , Y. Zhang , and C. Zhang , “A Capacity Model Based on Charging Process for state of Health Estimation of Lithium Ion Batteries,” Applied energy 177 (2016): 537–543.

[77] J. He , Z. Wei , X. Bian , and F. Yan , “State‐of‐Health Estimation of Lithium‐Ion Batteries Using Incremental Capacity Analysis Based on Voltage–Capacity Model,” IEEE Transactions on Transportation Electrification 6, no. 2 (2020): 417–426.

[78] Y. Gao and S. Onori , “Advancing SOC Estimation in LiFePO_4_ Batteries: Enhanced dQ/dV Curve and Short‐pulse Methods,” eTransportation 26 (2025): 100466.

[79] T. Dao , “Flashattention‐2: Faster Attention with Better Parallelism and Work Partitioning,” International Conference on Learning Representations (2024): 35549–35562.

[80] H. Zhou , S. Zhang , J. Peng , et al., “Informer: beyond Efficient Transformer for Long Sequence Time‐series Forecasting,” Proceedings of the AAAI Conference on Artificial Intelligence 35, no. 12 (2021): 11106–11115.

