# Supplementary material for: Performance–Complexity Trade‐Offs in Battery Lifetime Prediction with Task‐Aware Transformers
Source: Adv Sci (Weinh). 2026 Jul 16:e24179. Online ahead of print. doi: 10.1002/advs.202524179 (PMC13373899; doi:10.1002/advs.202524179)
Supplement: Supplementary file 1 — Supporting File: advs76597‐sup‐0001‐SuppMat.pdf. [file ADVS-9999-e24179-s001.pdf]

Supplementary Information

Performance–Complexity Trade-offs in Battery Lifetime Prediction with Task-Aware Transformers

Jingyuan Zhao[1, *], Misheng Cai[2], Zhenghong Wang[2], Yan, Wang[3], Yuqi Li[4], Bo Dong[5], Hewu Wang[6,*], Andrew F. Burke[1], Simona Onori[7], Stephen J. Harris[8]

[1] Institute of Transportation Studies, University of California Davis, Davis, CA 95616, USA.

[2] School of Automotive and Traffic Engineering, Hubei University of Arts and Science, Xiangyang, Hubei 441503, China.

[3] Qingdao University of Technology, Huangdao District, Qingdao City, Shandong 266520, China.

[4] Department of Materials Science and Engineering, Stanford University, Stanford, CA 94305, USA.

[5] Department of Control Science and Engineering, Changchun University of Technology, Changchun, Jilin 130012, China.

[6] School of Vehicle and Mobility, Tsinghua University, Haidian District, Beijing 100084, China.

[7] Department of Energy Science and Engineering, Stanford University, Stanford, CA 94305, USA.

[8] Energy Storage and Distributed Resources Division, Lawrence Berkeley National Laboratory, Berkeley, CA 94720, USA.

Correspondence: jyzhao@ucdavis.edu (J.Z.); wanghw@tsinghua.edu.cn (H.W.)

Contents

Supplementary Information 1

Supplementary Note 1 Evaluation Metrics 3

Supplementary Note 2 Datasets 4

Supplementary Note 3.1 Prediction Performance Across Datasets and Protocols 6

Supplementary Note 3.2 Training-Test Similarity and Cross-Chemistry Prediction Performance 9

Supplementary Note 3.3 Battery Failure Probability Estimation 12

Supplementary Note 3.4 Feature-Space Similarity Under Different Training–Testing Configurations 14

Supplementary Note 3.5 Influence of State of Charge Window 18

Supplementary Note 3.6 Effect of Sampling Interval and Data Sparsity 19

Supplementary Note 3.7 Baseline Model Implementation and Comparative Performance 21

Supplementary Note 3.8 Model Parameter Calculation 24

Supplementary Note 3.9 Trade-off Analysis Between Model Complexity and Predictive Accuracy 25

Supplementary Note 4.0 Pruning-Based Complexity Reduction Analysis 33

Supplementary Note 4.1 Input Feature Construction and Attention-Aware Analysis 37

Supplementary Note 4.2 Hyperparameter Sensitivity Analysis 41

Supplementary Note 4.3 Training Configuration and Hyperparameter Settings 43

Supplementary Note 4.4 Weight Optimization 44

Supplementary Note 1 Evaluation Metrics

Model performance for RUL prediction was evaluated using four standard metrics: root mean squared error (RMSE), coefficient of determination (R^2), weighted mean absolute percentage error (WMAPE), and mean absolute error (MAE).

RMSE for RUL prediction is defined as:

$$\mathrm{RMSE} = \sqrt{\frac{1}{n}\sum_{i=1}^{n}\left(y_i - \hat{y}_i\right)^2}\ , \tag{1}$$

where $\hat{y}_i$ is the predicted RUL at the ith cycle and y_i is the corresponding actual RUL. n is the total number of cycles in a single cell.

R^2 is defined as:

$$R^2 = 1 - \frac{\sum_{i=1}^{n}\left(\hat{y}_i - \bar{y}\right)^2}{\sum_{i=1}^{n}\left(y_i - \bar{y}\right)^2}\ , \tag{2}$$

where $\bar{y} = \sum_{i=1}^{n} y_i \Big/ n$;

WMAPE is defined as:

$$\mathrm{WMAPE} = \frac{1}{\sum_{i=1}^{n} y_i}\sum_{i=1}^{n}\left|y_i - \hat{y}_i\right|, \tag{3}$$

MAE is defined as:

$$\mathrm{MAE} = \frac{1}{n}\sum_{i=1}^{n}\left|y_i - \hat{y}_i\right|, \tag{4}$$

Supplementary Note 2 Datasets

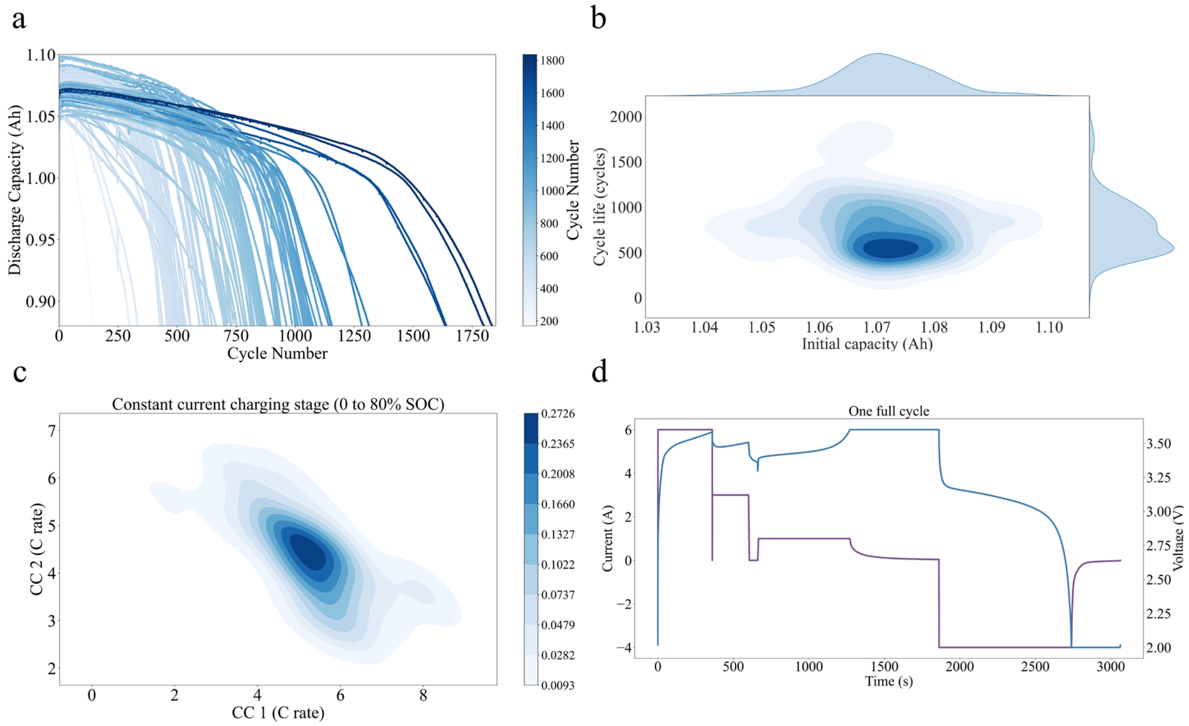

Supplementary Figure 1: Characteristics of battery cycle aging data for 118 LFP cells. (a). Discharge capacity versus cycle number, with curve color transitioning with increasing cycles. (b). Cycle life versus initial capacity, with color indicating data density. (c). Distribution of charging current rates in single-step and two-step fast-charging protocols across the 0–80% SOC range. (d). Voltage and current profiles over time during a full charge–discharge cycle.

Supplementary Table 1: Overview of the datasets used in this study.

Dataset	Battery information	Charge-discharge strategies/number of charge-discharge strategies	Temperature	Degradation range	Cycle life
#1	Battery count: 77; Nominal voltage: 3.3V; Nominal capacity: 1.1 Ah; Cutoff voltage: 3.6V, 2.0V; chemical compositions: lithium iron phosphate (LFP)/graphite.	Charging: 0-80% state of charge (SOC) at 5C (CC); 80% SOC-3.6V at 1C (CC); CV charging to cutoff current C/20 (one identical fast-charging protocol); Discharging: 100%-60% SOC; (rest 30s); 60%-40% SOC; (rest 30s); 40%-20% SOC; (rest 30s); SOC 20%-0% SOC (77 different multi-stage discharge protocols).	30°C	100%-80% SOH	1100 to 2700 cycles
#2	Battery count: 118*; Nominal voltage: 3.3V Nominal capacity: 1.1Ah Cutoff voltage: 3.6V; 2.0V;	Charging: 0%-80% SOC, one or two-step charging to 80% SOC using constant current, charging rates include: 3C, 3.6C, 4C, 4.4C, 4.8C, 5.4C, 6C, 7C, 8C, and so on,	30°C	100%-80% SOH	150 to 2300 cycles

	chemical compositions: LFP/graphite.	with different charging SOC ranges; 80% SOC -3.6V, charged at 1C until reaching 3.6V then CV charging to cutoff current C/50 (72 different fast-charging protocols); Discharging: unified discharge strategy of constant current and constant voltage discharge, discharge rate of 4C to 2.0V, cutoff discharge current C/50 (one identical discharging protocol);			
#3	Battery count: 19; Nominal voltage: 3.6V; Nominal capacity: 3.5 Ah; Cutoff voltage: 4.2V, 2.65V; chemical compositions: lithium nickel cobalt aluminum oxide (NCA) /graphite.	Charging: 0% SOC -4.2V at 0.5C (CC); CV charging to cutoff current C/20 (one identical charging protocol); Rest: 30min; Discharging: 100% SOC-2.65V at 1C (CC) (one identical discharging protocol; Rest: 30min.	25°C	100% - (92%~77%) SOH	107 to 207 cycles
#4	Battery count: 22; Nominal voltage: 3.6V Nominal capacity: 3Ah Cutoff voltage: 4.2V; 2V; chemical compositions: lithium-manganese-cobalt-oxide (NMC)/graphite.	Charging: 0% SOC -4.2V at 0.5C (CC); CV charging to cutoff current C/20 (one identical charging protocol); Discharging: 100% SOC-2V at 0.5C, 1C, 2C, and 3C (CC) (four identical discharging protocol;	15°C, 25°C, 35°C	100% - (81%~60%) SOH	380 to 1321 cycles

* Cells with anomalous data (e.g., cycling tests that were accidentally terminated) are not considered for model training and testing.

Supplementary Note 3.1 Prediction Performance Across Datasets and Protocols

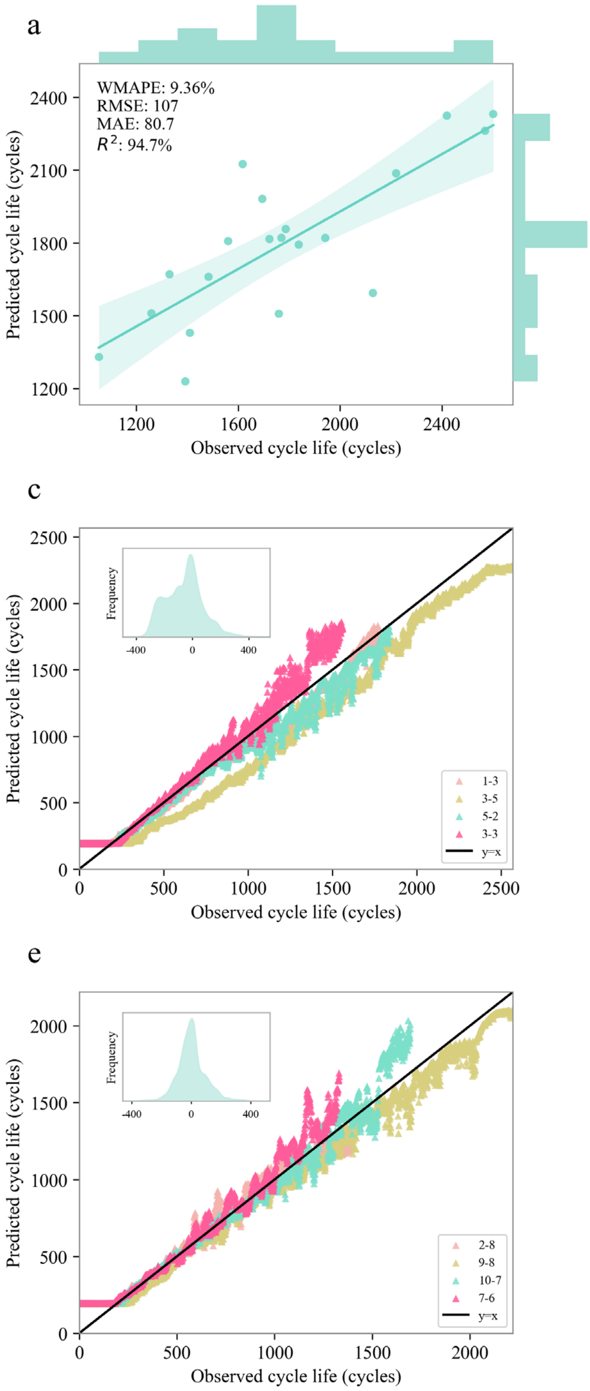

Supplementary Figure 2: Prediction results and error distribution for 20 test cells operating under distinct discharging protocols in dataset 1. (a) displays the overall distribution of predicted RUL versus actual RUL for the 20 batteries. (b)-(f) show the Cell level prediction results for different battery samples. The black line denotes the ideal prediction line, where the predicted cycle life equals the observed cycle life. Colored markers represent different battery samples, and the battery labels follow the original file names in Dataset 1, such as “2-4”. The inset in each panel shows the distribution of prediction residuals, reflecting the bias and dispersion of model errors.

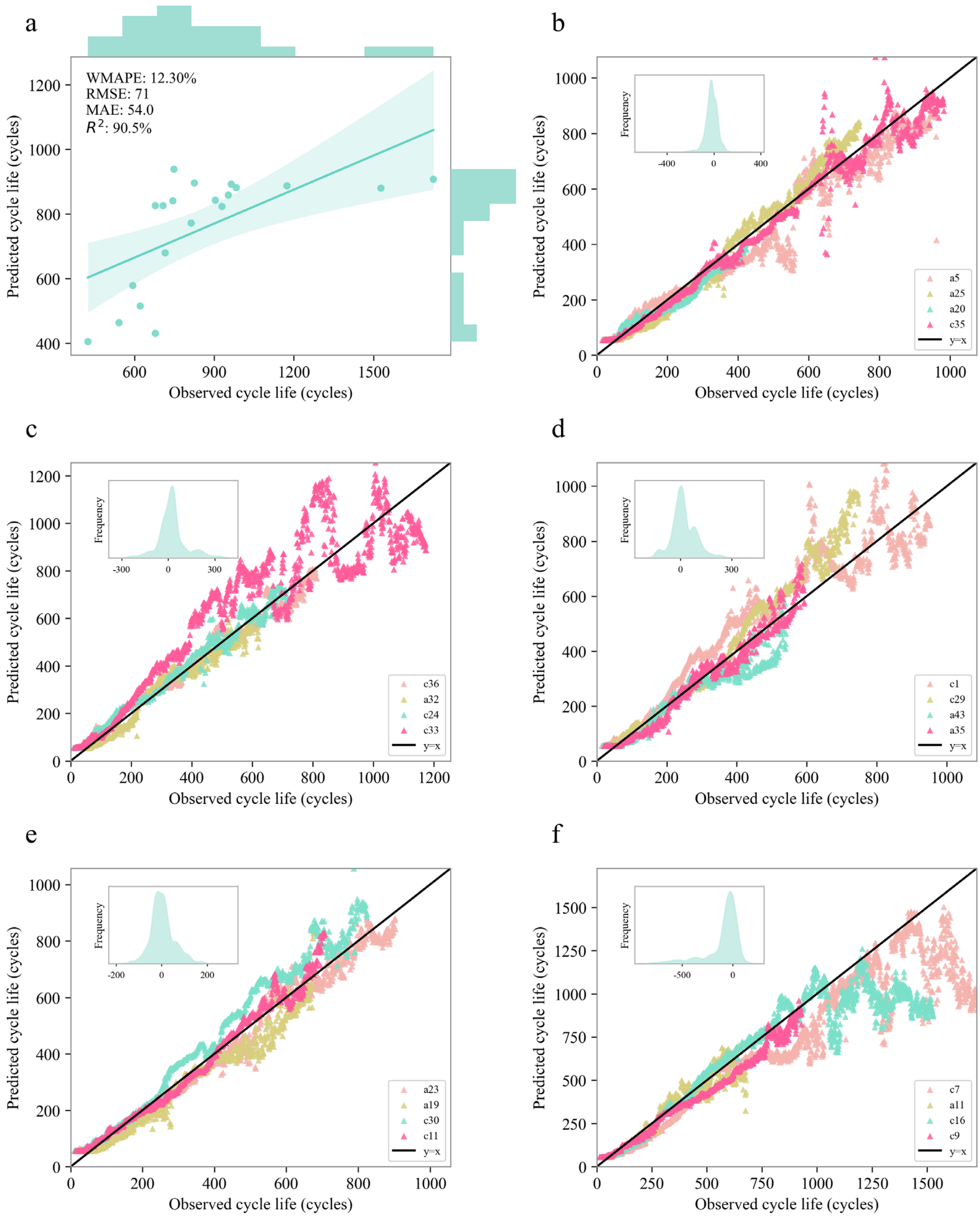

Supplementary Figure 3: Prediction performance and error distribution for a representative subset of 20 test cells from the 36-cell test set in dataset 2. The reported evaluation metrics were calculated using the full test set. The black line denotes the ideal prediction line, where predicted cycle life equals observed cycle life. Colored markers represent different battery samples, with labels following the original file names in dataset 2, such as “a5”. The inset in each panel shows the residual distribution, reflecting model bias and error dispersion.

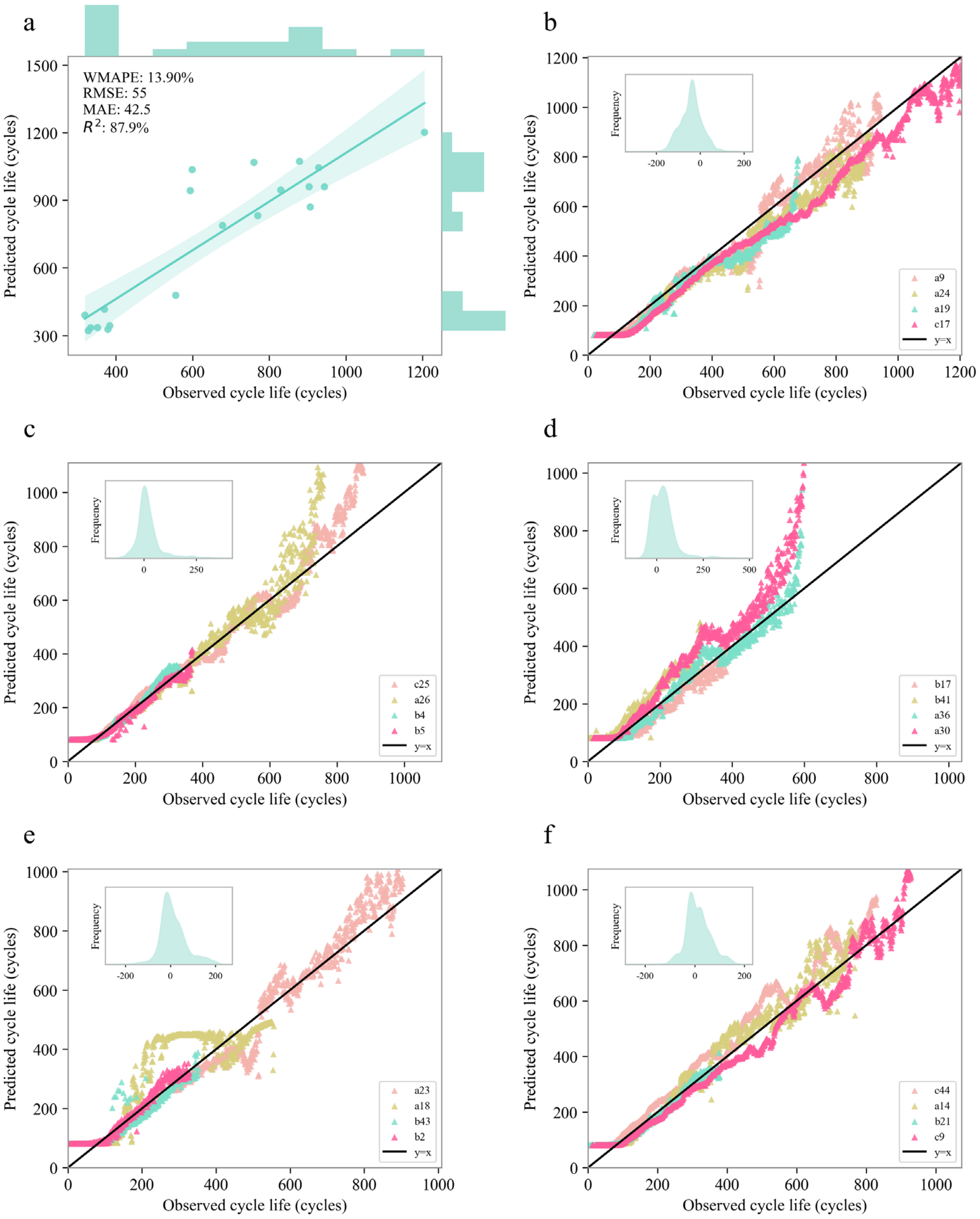

Supplementary Figure 4: Prediction performance and error distribution for 20 representative cells selected from the 36-cell test set of dataset 2 under different fast-charging protocols. Panel (a) shows the overall relationship between observed and predicted cycle life across the 20 testing protocols, together with marginal distributions of the observed and predicted cycle life. The inset reports the overall evaluation metrics, including WMAPE, RMSE, MAE, and R^2. Panels (b-f) show predicted versus observed cycle life for different groups of testing protocols that were excluded from the training set. The black diagonal line denotes the ideal prediction line, y=x, and triangular markers represent individual cycle-level predictions under different testing protocols. The inset density plots in panels (b-f) show the corresponding prediction error distributions.

Supplementary Note 3.2 Training-Test Similarity and Cross-Chemistry Prediction Performance

To quantify the differences in charging behavior characteristics among battery samples, a similarity measurement framework based on a three-dimensional feature space was constructed. Specifically, three key indicators were extracted from the charging data of each cell: average charging capacity, average charging voltage, and average cycle count. These indicators were normalized to generate a three-dimensional feature vector for each sample, representing the distributional characteristics of training and testing batteries in the charging behavior space. The Euclidean distance was subsequently employed to compute a distance matrix between the training and test sets, thereby providing a quantitative assessment of sample proximity in the constructed feature space.

$$d(x, y) = \sqrt{\sum_{i=1}^{n} (x_i - y_i)^2}, \tag{5}$$

where x and y denote the feature vectors of two samples in an n-dimensional space.

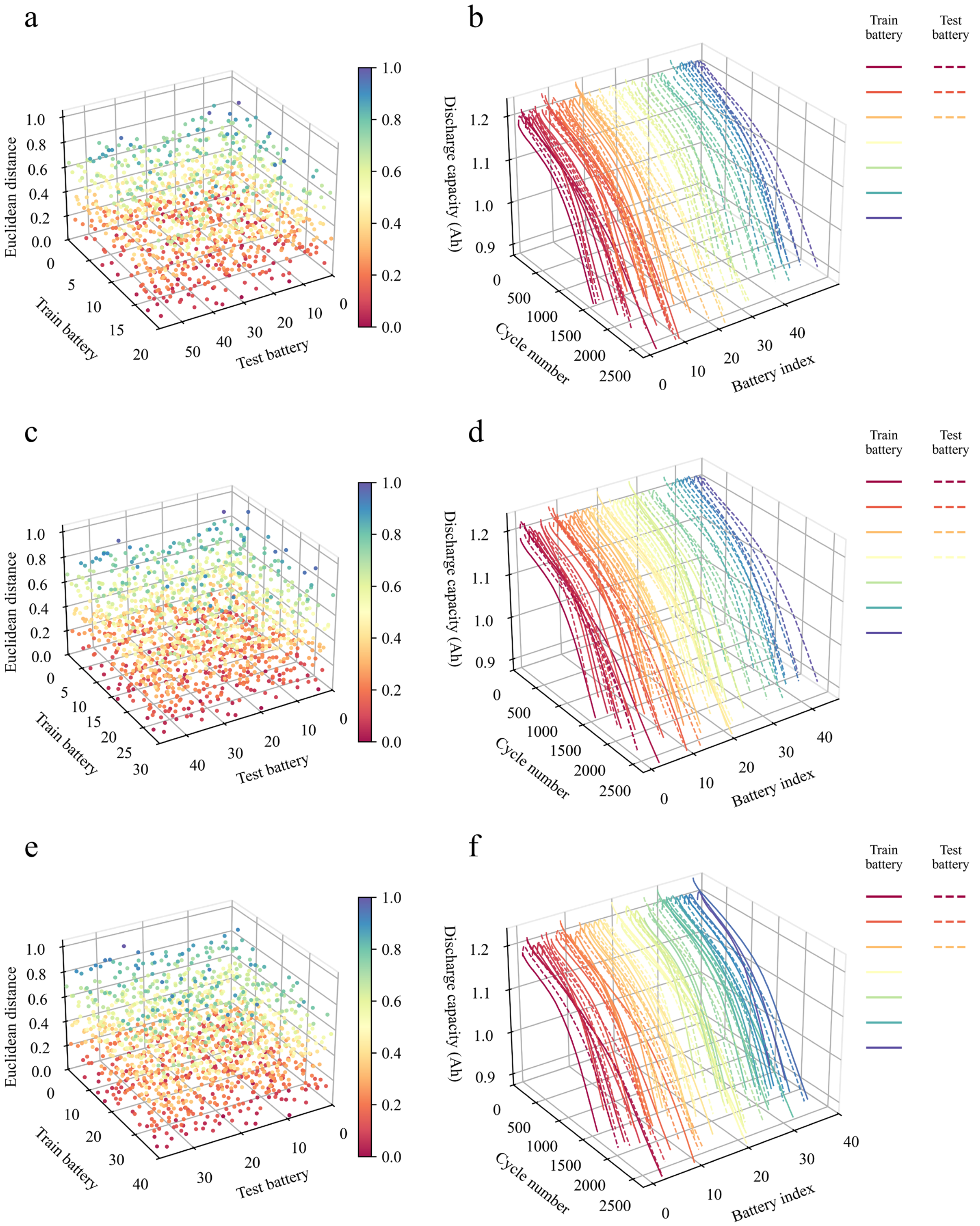

Supplementary Figure 5: Visualization of training–testing similarity and discharge capacity degradation curves. Panels (a), (c), and (e) show the Euclidean distance between each test battery and the training batteries, indicating distributional similarity in the feature space. Panels (b), (d), and (f) illustrate the discharge capacity curves of training and test batteries over cycle life, demonstrating differences in degradation patterns across datasets. Solid lines represent training batteries, while dashed lines denote test batteries.

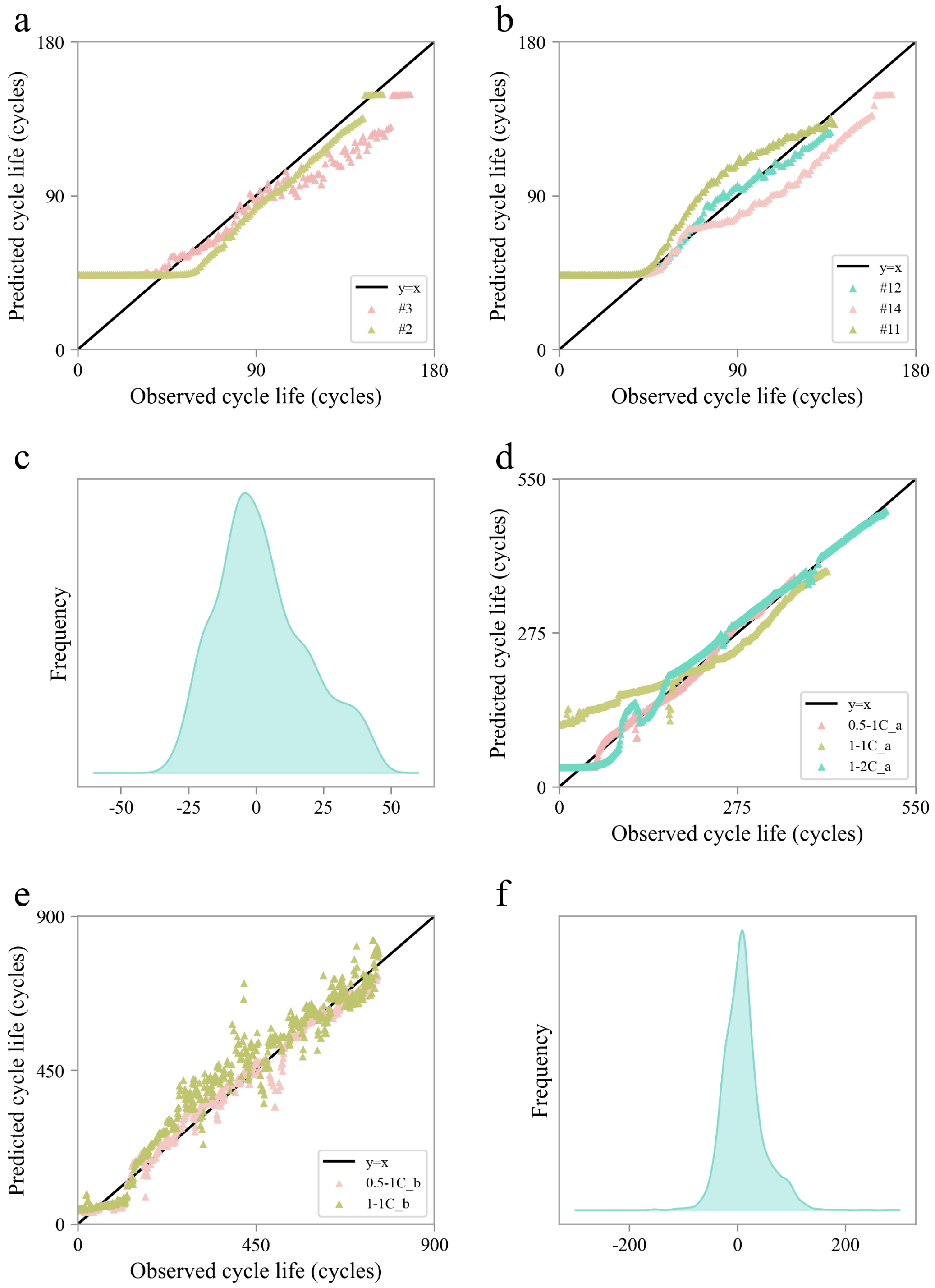

Supplementary Figure 6: Prediction performance and error distribution across different chemical materials in dataset 3 and 4. (a), (b) Predicted versus observed cycle life for dataset 3, where battery labels are presented using cell-index notation. (c) Error distribution for all NCA samples. (d), (e) Predicted versus observed cycle life for dataset 4, where battery labels are presented using protocol-based C-rate identifiers, including 0.5-1C_a, 1-1C_a, and 1-2C_a. (f) Error distribution for all SNL NMC samples. The black diagonal line denotes the ideal prediction line, y = x, and the triangular markers denote predictions for individual batteries or cycling conditions.

Supplementary Note 3.3 Battery Failure Probability Estimation

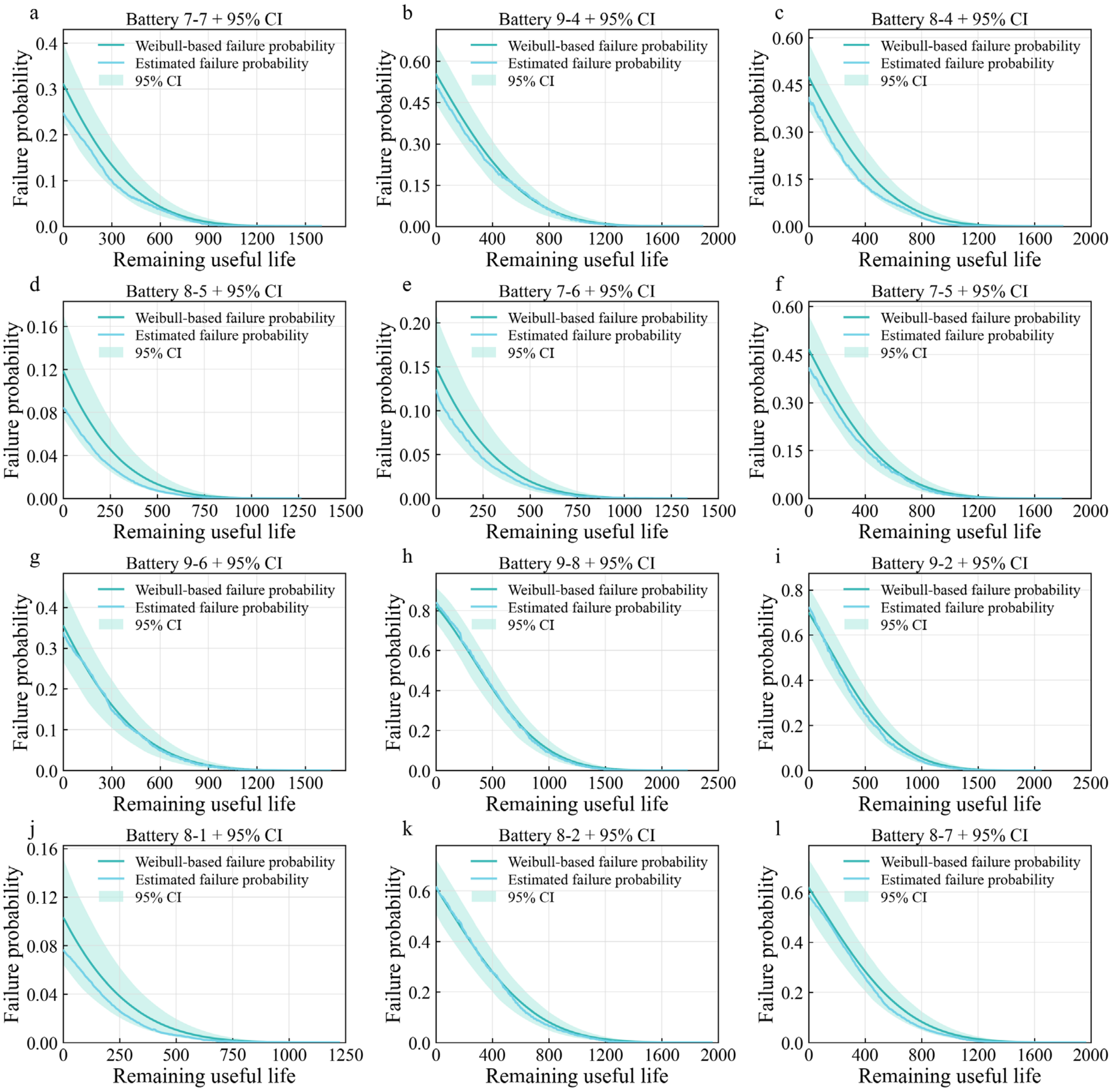

Supplementary Figure 7: Estimated battery failure probability curves compared with Weibull-based reference curves across 12 test samples (a–l). The cyan line represents the failure probability predicted by the deep learning model at each cycle. The dark turquoise line indicates the reference failure probability fitted using the Weibull distribution, while the shaded region shows the 95% confidence interval. Each subplot shows the agreement between model-estimated failure probability and Weibull-based reference curves across different test samples, indicating that the model captures Weibull-consistent population-level failure trends. Battery labels, such as "7-7", follow the original file names in dataset 1, with each file corresponding to one individual battery sample.

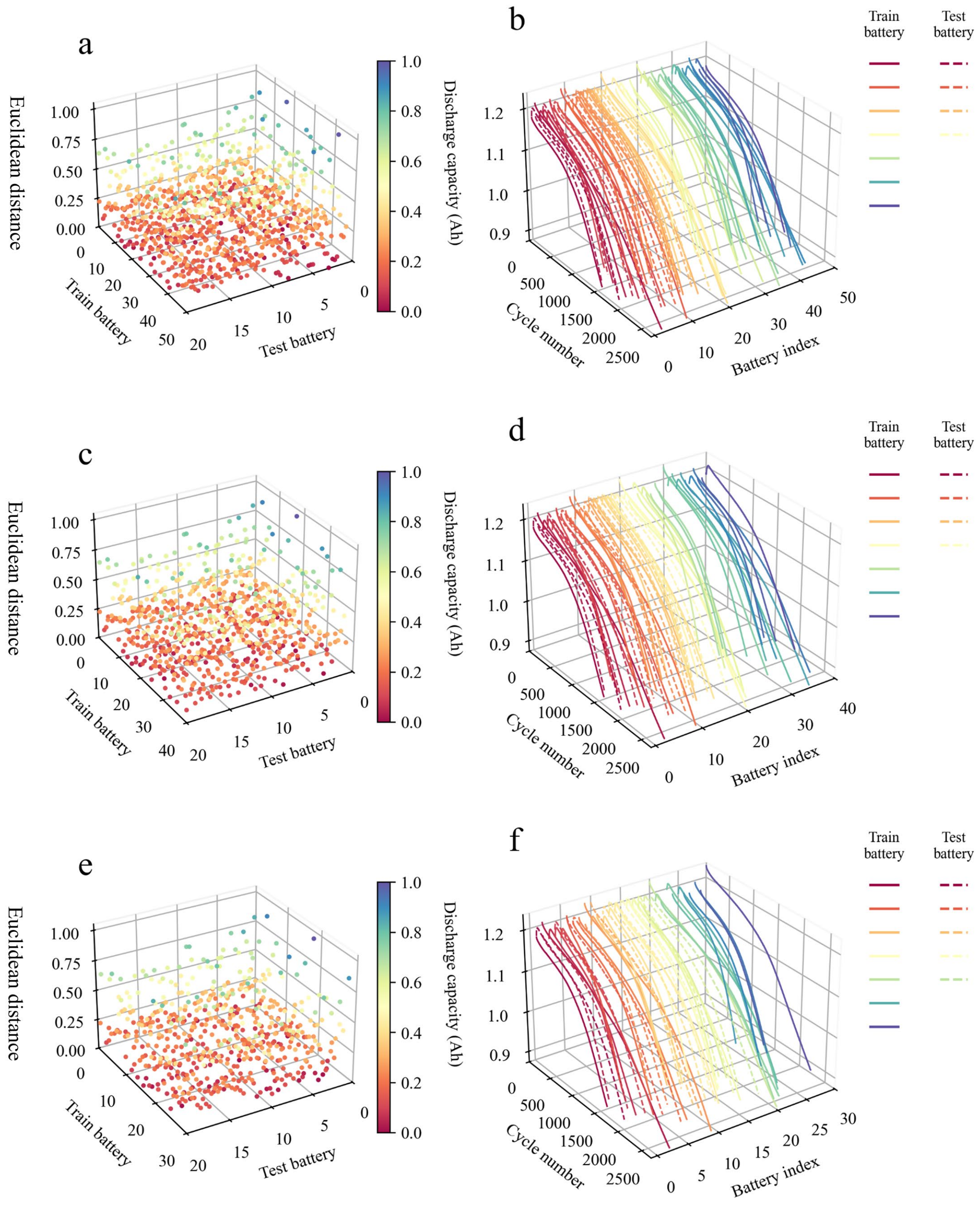

Supplementary Figure 8: Visualization of distribution characteristics and discharge capacity degradation patterns under varying training set sizes (with a fixed test set of 20 batteries) in dataset 1. Panels (a), (c), and (e) show the Euclidean distance distributions between training and test batteries under training dataset sizes of 50, 40, and 30 batteries, respectively, with the test set fixed at 20 batteries. The color scale represents normalized discharge capacity. Panels (b), (d), and (f) show the corresponding discharge capacity degradation trajectories, where solid lines denote training batteries and dashed lines denote test batteries. Specifically, panels (a) and (b), (c) and (d), (e) and (f) correspond to training dataset sizes of 50, 40, and 30 batteries, respectively.

Supplementary Note 3.4 Feature-Space Similarity Under Different Training–Testing Configurations

Under different training–testing dataset configurations, the normalized Euclidean distance was computed using mean charging voltage, mean charging capacity, and average cycle count to quantify feature-space similarity between the training and test sets. As summarized in Table S2, under the fixed 20-battery test setting, the mean Euclidean distance varied from 0.4682 to 0.6773 as the training size changed from 50 to 30 batteries, indicating that training-set representativeness, rather than sample size alone, affects training–testing similarity. The 50/20 configuration showed the smallest mean and median distances, 0.4682 and 0.4686, respectively, which is consistent with its relatively low prediction errors in Table S3. By contrast, the 30/20 configuration showed the largest feature-space discrepancy among the fixed-test configurations, with a mean distance of 0.6773 and a median distance of 0.6801, corresponding to weaker prediction performance.

When both the training and test sets contained 20 batteries, the mean Euclidean distance was 0.4874 and the median was 0.5373. When the training set remained fixed at 20 batteries and the test set was expanded to 57 batteries, the mean distance increased from 0.4874 to 0.7179, and the median distance increased from 0.5373 to 0.7232. This increase indicates that enlarging the test set under limited training coverage introduces a broader feature distribution and a higher degree of discrepancy between the training and test data. Therefore, the expanded test configuration presents a stronger distribution shift, which may increase the difficulty of model generalization under limited training samples. Overall, these results suggest that model performance is governed not only by the number of training batteries, but also by the feature-space alignment between training and test samples. Thus, Euclidean distance provides a useful diagnostic indicator for interpreting generalization difficulty, although it should be considered together with training sample size, degradation-pathway diversity, and prediction metrics.

Supplementary Table 2: Mean and median Euclidean distances under different training and testing battery set sizes.

Train size (batteries)	Test size (batteries)	Mean Euclidean distance	Median Euclidean distance
57	20	0.6247	0.6303
50	20	0.4682	0.4686
40	20	0.5934	0.6031

30	20	0.6773	0.6801
20	20	0.4874	0.5373
20	57	0.7179	0.7232
30	47	0.5611	0.5600
40	37	0.6063	0.6172

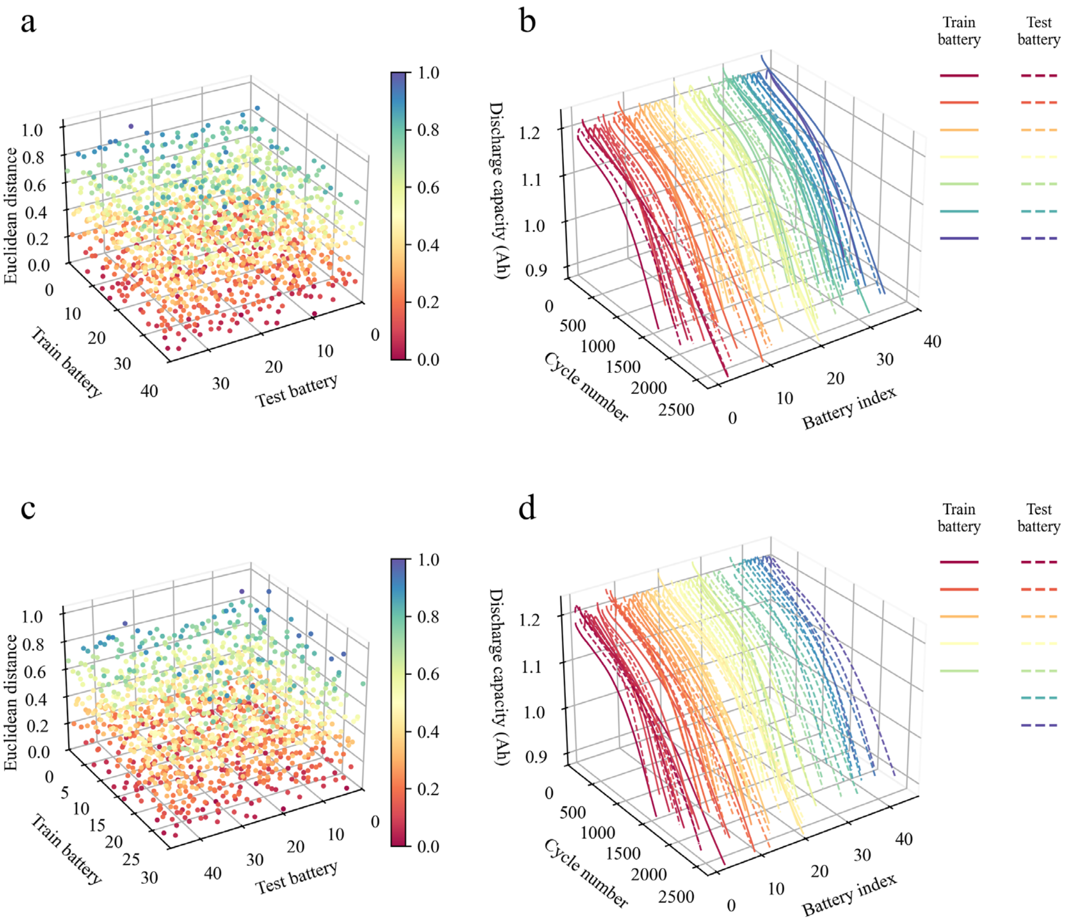

Supplementary Figure 9: Visualization of distribution characteristics and discharge capacity degradation patterns in dataset 1 under different training and testing set configurations. Panels (a) and (c) show the Euclidean distance distributions between training and test batteries under two different data split configurations, with the color scale representing normalized discharge capacity. Panels (b) and (d) show the corresponding discharge capacity degradation trajectories, where solid lines denote training batteries and dashed lines denote test batteries. Specifically, panels (a) and (b) correspond to the configuration with 40 batteries in the training set and 37 batteries in the test set, whereas panels (c) and (d) correspond to the configuration with 30 batteries in the training set and 47 batteries in the test set.

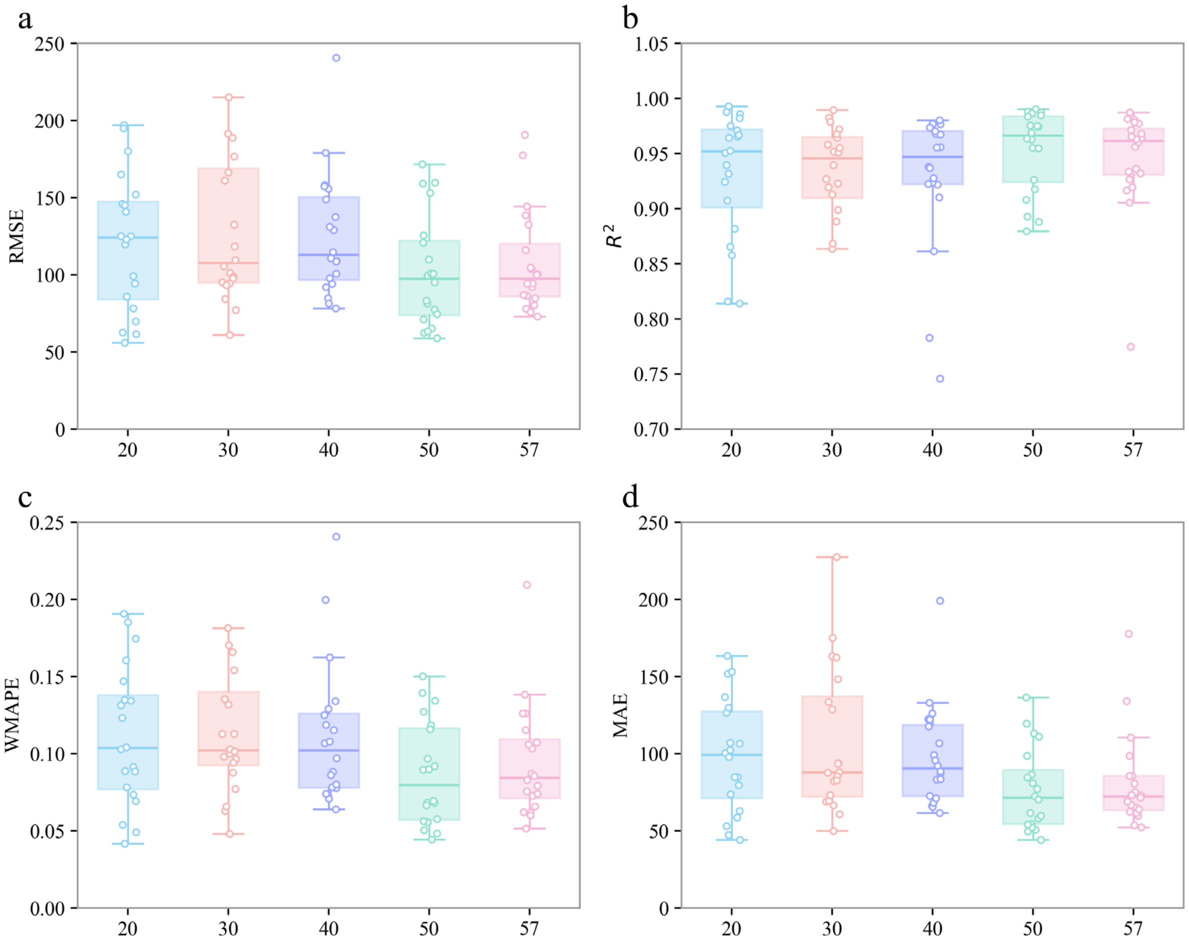

Supplementary Figure 10: Performance of varying training dataset sizes (20, 30, 40, 50, and 57). Panels (a)-(d) show the distributions of RMSE, R^2, WMAPE, and MAE, respectively, under different training dataset sizes of 20, 30, 40, 50, and 57 batteries. Box plots summarize the distribution of each metric across repeated experiments or test batteries, with scattered points indicating individual results.

Supplementary Table 3: Results under different training dataset sizes.

Various categories	Training dataset size / Test dataset size (batteries)	RMSE (cycles)	R^2	WMAPE (%)	MAE (cycles)
Fixed test dataset size	57/20	113	0.960	9.00	81.6
	50/20	101	0.954	8.67	76.8
	40/20	124	0.931	10.80	95.5
	30/20	132	0.921	11.80	106
	20/20	123	0.930	11.30	99.9
Variable test dataset size	40/37	123	0.932	10.30	93.8
	30/47	132	0.918	11.20	98.0
	20/57	139	0.905	11.80	106

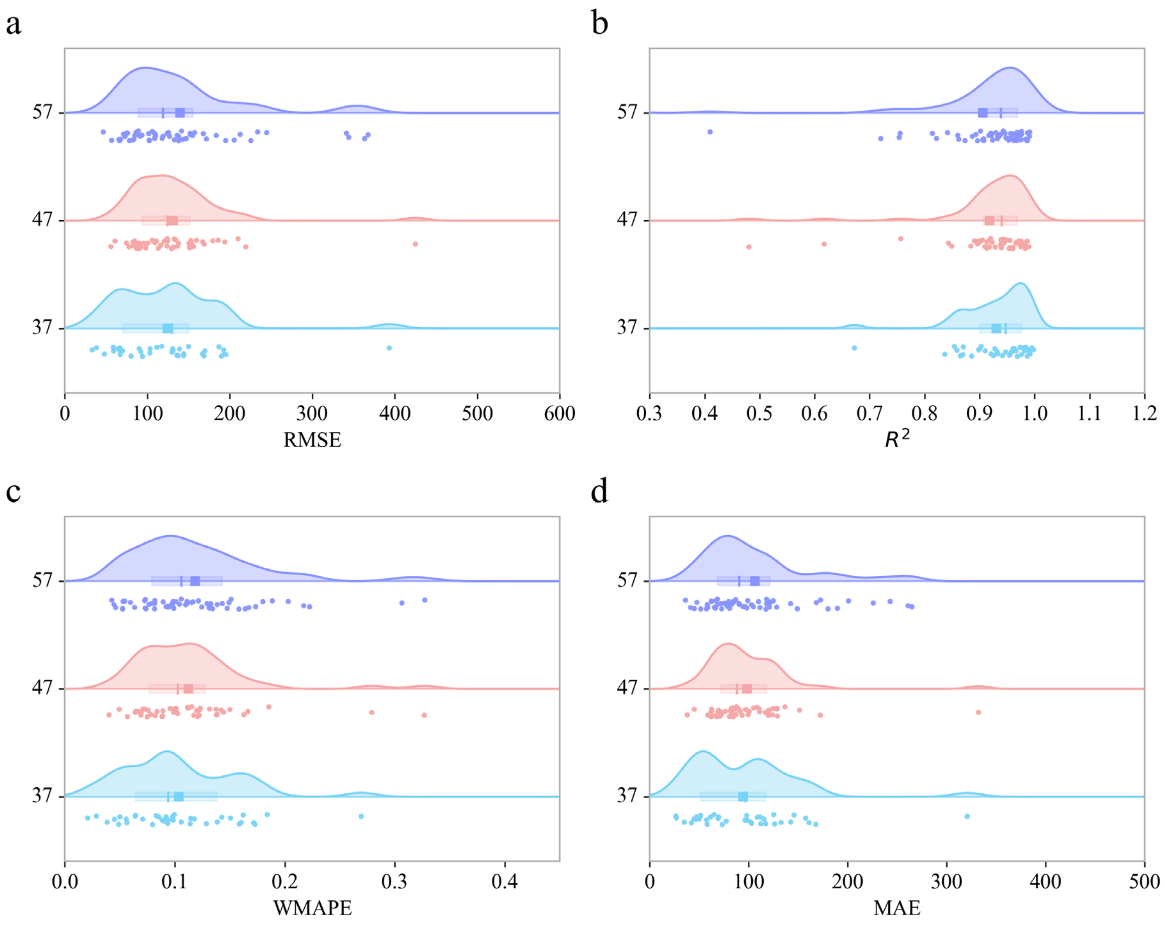

Supplementary Figure 11: Performance of varying test dataset sizes (37, 47, and 57). Panels (a), (b), (c), and (d) show the distributions of RMSE, R^2, WMAPE, and MAE, respectively, under different test dataset sizes of 37, 47, and 57 batteries. Ridge density curves show the distribution of each metric, box plots indicate the median and interquartile range, and scattered points represent individual test results.

Supplementary Note 3.5 Influence of State of Charge Window

Supplementary Table 4: Results across different SOC ranges.

SOC range	RMSE (cycles)	R^2	WMAPE	MAE (cycles)
80%-96%	96	0.958	0.0827	73.7
80%-94%	113	0.936	0.0998	87.0
80%-93%	109	0.942	0.0930	83.6
80%-92%	113	0.935	0.0943	83.8
80%-90%	112	0.937	0.0968	86.7

Supplementary Note 3.6 Effect of Sampling Interval and Data Sparsity

Data density directly affects the accuracy of model predictions. To explore model performance under different levels of data sparsity, the data were processed using sampling intervals of 10, 20, 30, 40, 50, 60, and 70 s, and model performance was evaluated using four error metrics: RMSE, R^2, WMAPE, and MAE. In the initial cycle, within the selected SOC and voltage range, the data were resampled using different sampling intervals from 10 s to 70 s to evaluate the influence of data sparsity on cycle life prediction. As the sampling interval increased, the number of available data points gradually decreased, resulting in progressively sparser charge trajectories. Under the 70 s sampling interval, the model achieved an RMSE of 122 cycles, R^2 of 0.934, WMAPE of 0.107, and MAE of 96.4 cycles, indicating that the model can still maintain stable prediction performance under sparse data conditions. By analyzing the error distribution across 20 test protocols (**Fig. S12** and **Table S5**), it can be observed that the prediction accuracy fluctuates with increasing sampling interval rather than decreasing monotonically. The best performance is obtained at the 30 s sampling interval, with an RMSE of 108 cycles, R^2 of 0.941, WMAPE of 0.0941, and MAE of 83.9 cycles. In contrast, the largest error appears at the 50 s sampling interval, where the RMSE, R^2, WMAPE, and MAE are 134 cycles, 0.921, 0.118, and 106 cycles, respectively. These results demonstrate that the model remains robust under sparse sampling conditions, although excessive sparsity can increase the prediction error to some extent. This outcome is primarily due to two key factors. First, the preprocessing method of linear interpolation, based on the interpolation of capacity and voltage over time, provides the model with a uniform time scale within each cycle dimension, ensuring that the reduction in data points does not drastically weaken the predictive ability of the model. Second, the combination of convolutional and attention mechanisms allows the model to effectively extract key information from sparse data across multiple time scales, further enhancing prediction robustness.

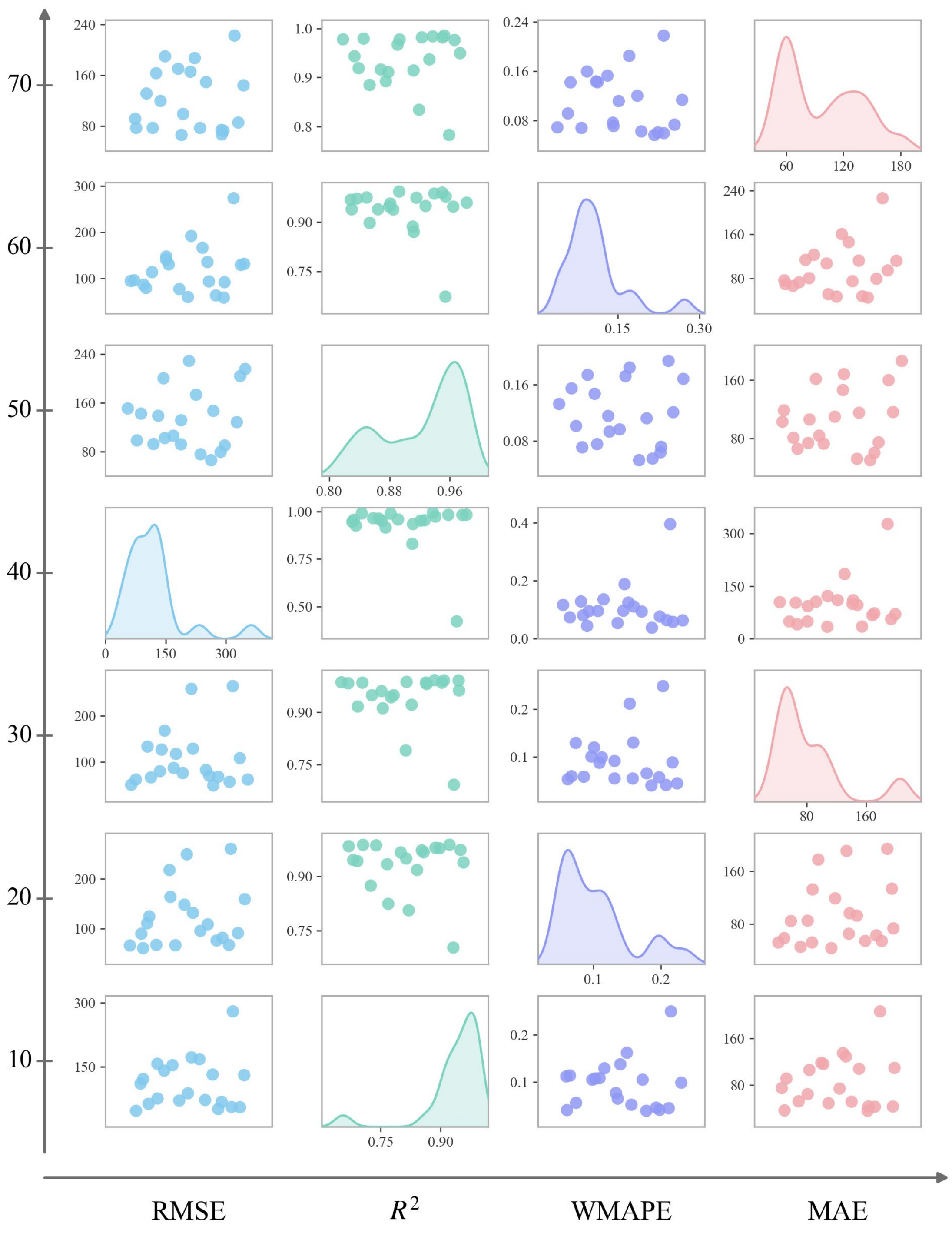

Supplementary Figure 12: Distribution of RMSE under different sampling intervals. The figure shows the distributions of RMSE, R^2, WMAPE, and MAE under different sampling intervals of charging data within the SOC range from 80% to the upper voltage limit. The rows correspond to sampling intervals of 10, 20, 30, 40, 50, 60, and 70 s, respectively, from bottom to top. The columns correspond to different evaluation metrics, namely RMSE, R^2, WMAPE, and MAE from left to right. Scatter plots show the metric values obtained for individual batteries, whereas density plots summarize the distribution of each metric under the corresponding sampling interval.

Supplementary Table 5: Results under varying data sparsity.

Sampling intervals	RMSE (cycles)	R^2	WMAPE	MAE (cycles)
10	111	0.938	0.094	83.8
20	121	0.932	0.101	92.2
30	108	0.941	0.094	83.9
40	120	0.927	0.107	96.3
50	134	0.921	0.118	106
60	117	0.936	0.106	94.5
70	122	0.934	0.107	96.4

Supplementary Note 3.7 Baseline Model Implementation and Comparative Performance

For comparison, several baseline models with distinct architectures were selected:

(a) CNN model: Consists of three layers of 2D convolution and pooling, followed by two fully connected layers.

(b) LSTM model: Comprises two LSTM layers, followed by two fully connected layers.

(c) CNN-LSTM model: Follows the same CNN and LSTM configuration, with two fully connected layers.

(d) CNN-Attention model: Incorporates three layers of 2D convolution and pooling, followed by two layers of multi-head self-attention and two fully connected layers.

(e) LSTM-Attention model: Includes two LSTM layers combined with multi-head self-attention.

(f) Vanilla Transformer model: Configured identically to the FAST model.

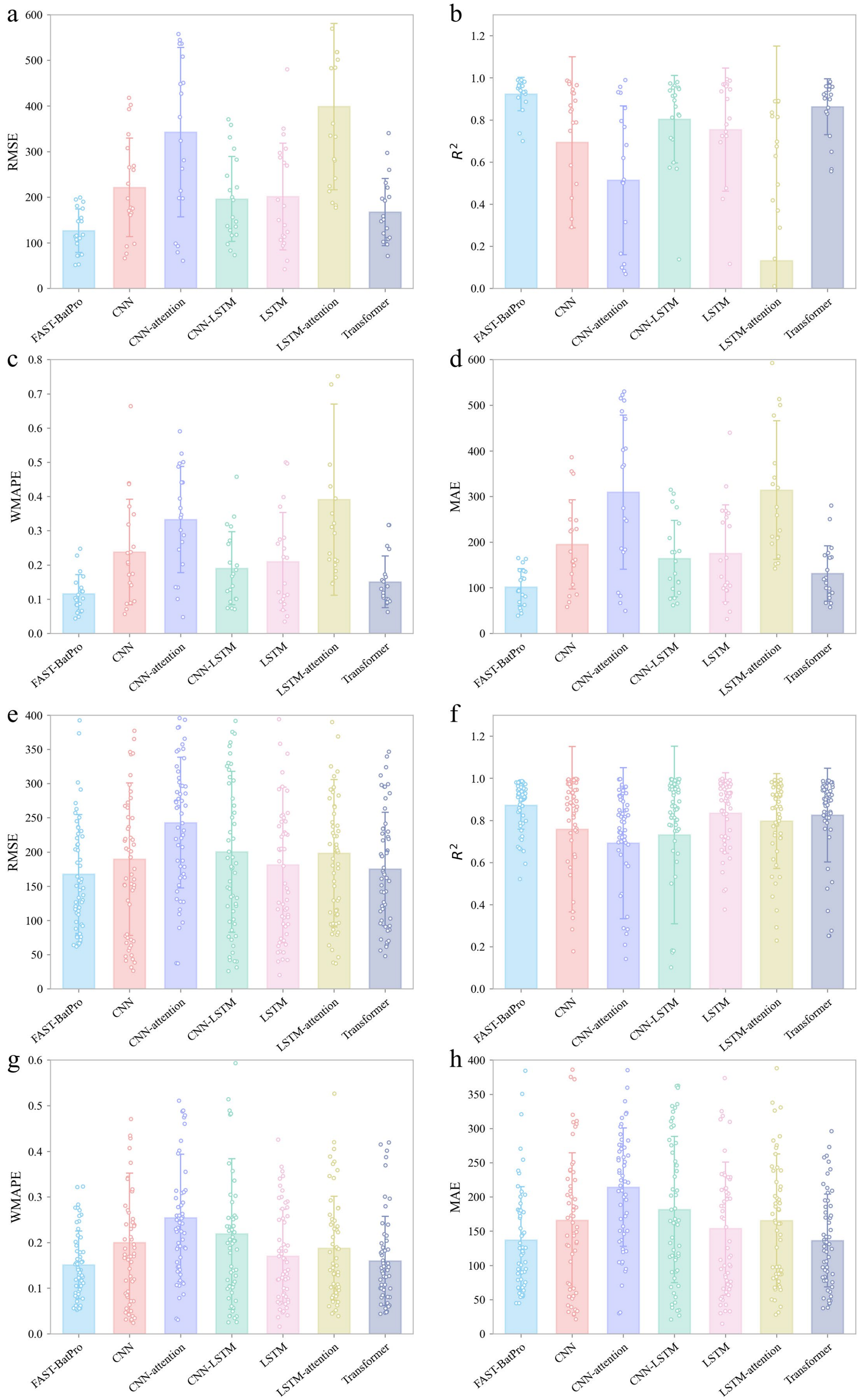

a
b
c
d
e
f
g
h
RMSE
R^2
WMAPE
MAE
FAST-BatPro
CNN
CNN-attention
CNN-LSTM
LSTM
LSTM-attention
Transformer

Supplementary Figure 13: Comparison of FAST-BatPro and other deep learning methods across different error metrics. Panels (a)-(d) show the distributions of RMSE, R^2, WMAPE, and MAE, respectively, under the configuration with 20 LFP batteries in the training set and 20 LFP batteries in the test set. Panels (e)-(h) show the corresponding distributions of RMSE, R^2, WMAPE, and MAE under the configuration with 20 LFP batteries in the training set and 57 LFP batteries in the test set. Bars represent the mean performance of each method, error bars indicate the variability across test batteries, and scattered points represent individual battery-level results.

Supplementary Table 6: Average metrics of battery cycle life prediction for various methods.

Various categories	Different algorithms	RMSE (cycles)	R^2	WMAPE	MAE (cycles)
Training dataset size / Test dataset size: 20/20	FAST-BatPro	126	0.927	0.114	101
	CNN	221	0.693	0.137	195
	CNN-attention	342	0.513	0.333	309
	CNN-LSTM	196	0.803	0.190	164
	LSTM	201	0.754	0.210	175
	LSTM-attention	398	0.132	0.391	314
	Transformer	167	0.863	0.150	131
Training dataset size / Test dataset size: 20/57	FAST-BatPro	166	0.878	0.147	135
	CNN	189	0.758	0.200	166
	CNN-attention	243	0.692	0.254	214
	CNN-LSTM	200	0.730	0.219	181
	LSTM	181	0.835	0.170	154
	LSTM-attention	198	0.797	0.188	166
	Transformer	175	0.825	0.160	136

Supplementary Note 3.8 Model Parameter Calculation

The model parameters are variables learned through a data-driven approach during the training process, primarily consisting of weights and biases in the neural network. The parameters of the constructed flash-attention-sparse Transformer model were calculated, with the model consisting of 17 network layers. The FAST-BatPro model comprises a total of 1.869 million trainable parameters when configured with a hidden layer dimension of 256. Model parameter counts corresponding to other hidden layer dimensions are summarized in **Table S7** to **Table S13**. This matrix operation is a crucial step in forward propagation and directly influences the model predictions (**Fig. S14**). As more data batches are processed, the model iteratively adjusts and optimizes the weight parameters during training, enhancing prediction accuracy. This efficient data processing not only accelerates the training process but also promotes faster model convergence. In contrast, hyperparameters act as higher-level control variables that regulate the learning behavior of the model during training. The appropriate setting and tuning of both model parameters and hyperparameters are essential for the overall performance of the model.

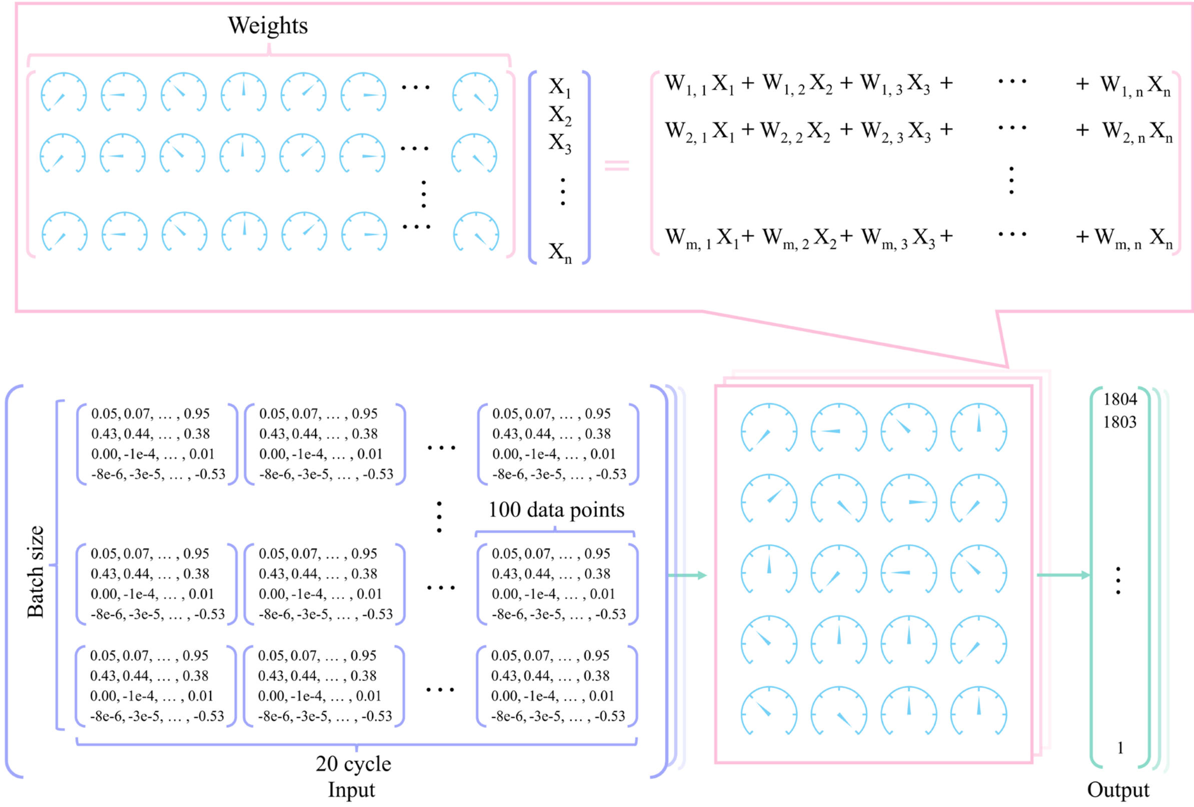

Supplementary Figure 14: A flowchart of the forward propagation process of the model. It illustrates how the model extracts feature and generates predictions through parameter–input mappings during training. This process constitutes a core component of supervised inference, enabling accurate and efficient output generation.

Supplementary Note 3.9 Trade-off Analysis Between Model Complexity and Predictive Accuracy

To quantify and evaluate the computational cost of the model, three primary metrics were used: inference time, floating-point operations (FLOPs), and energy consumption. Inference time refers to the duration required by the model to generate a prediction from input data, directly reflecting its operational efficiency in real-world applications. FLOPs provide a measure of computational complexity by quantifying the number of arithmetic operations performed during inference. In addition, energy consumption, estimated as the product of GPU power draw and inference time, offers a practical indicator of hardware-level resource usage and is particularly important when considering deployment on power-constrained platforms. To this end, an NVIDIA GeForce RTX 4090 Desktop GPU, equipped with 32 GB of dedicated GDDR6 VRAM and up to 15.6 GB of shared system memory, was used to process a test dataset comprising 20 distinct charge-discharge protocols and 35,481 cycles in total. The data were processed in batches, with each batch containing 2,000 cycles. The proposed large time-series model used partial charging data from 20 cycles (each cycle containing 400 data points) to predict the RUL of the battery for each cycle. During the testing phase, the model achieved an average inference time of 0.103 seconds per battery and approximately 1.65 billion FLOPs per inference. The corresponding energy consumption was calculated using the formula $E = P * t$, where P denotes the GPU power draw and t the inference time. Given that the thermal design power (TDP) of the NVIDIA GeForce RTX 4090 Desktop GPU is 450 W, a conservative power value of 450 W was used to estimate the upper-bound inference energy consumption. The resulting energy in joules was converted into watt-hours using the standard conversion factor of 1 Wh = 3600 J.

Supplementary Table 7: Model parameter (hidden layer dimension: 256).

Index		Layer	Weight parameters	Total trainable parameters
1	Encoder module	Token embedding	400 * 256 * 3	307,200
2		Flash self-attention	256 * 768 + 256 * 256	262,144
3		Residual connection and layer normalization	256	256
4		Feedforward neural networks (FNN)	256 * 256 * 2	131,072
5		Residual connection and layer normalization	256	256
6		1-D CNN	3 *256 * 256 + 256	196,864
7		Layer normalization	256	256
8	Decoder module	Token embedding	400 * 256 * 3	307,200
9		Masked probsparse self-attention	4 * 256 * 256	262,144
10		Residual connection and layer normalization	256	256
11		Flash cross-attention	2 * 256 * 256 + 256 * 512	262,144
12		Residual connection and layer normalization	256	256
13		FNN	256 * 256 * 2	131,072
14		Residual connection and layer normalization	256	256
15		Layer normalization	256	256
16	Output layer	Full connected	256 * 1	256
17		Full connected	20 * 1	20

Supplementary Table 8: Model parameter (hidden layer dimension: 128).

Index		Layer	Weight parameters	Total trainable parameters
1	Encoder module	Token embedding	400 * 128 * 3	153,600
2		Flash self-attention	128 * 384 + 128 * 128	65,536
3		Residual connection and layer normalization	128	128
4		FNN	128 * 128 * 2	32,768
5		Residual connection and layer normalization	128	128
6		1-D CNN	3 *128 * 128 + 128	49,280
7		Layer normalization	128	128
8	Decoder module	Token embedding	400 * 128 * 3	153,600
9		Masked probsparse self-attention	4 * 128 * 128	65,536
10		Residual connection and layer normalization	128	128
11		Flash cross-attention	2 * 128 * 128 + 128 * 256	65,536
12		Residual connection and layer normalization	128	128
13		FNN	128 * 128 * 2	32,768
14		Residual connection and layer normalization	128	128
15		Layer normalization	128	128
16	Output layer	Full connected	128 * 1	128
17		Full connected	20 * 1	20

Supplementary Table 9: Model parameter (hidden layer dimension: 64).

Index		Layer	Weight parameters	Total trainable parameters
1	Encoder module	Token embedding	400 * 64 * 3	76,800
2		Flash self-attention	64 * 192 + 64 * 64	16,384
3		Residual connection and layer normalization	64	64
4		FNN	64 * 64 * 2	8,192
5		Residual connection and layer normalization	64	64
6		1-D CNN	3 * 64 * 64 + 64	12,352
7		Layer normalization	64	64
8	Decoder module	Token embedding	400 * 64 * 3	76,800
9		Masked probsparse self-attention	4 * 64 * 64	16,384
10		Residual connection and layer normalization	64	64
11		Flash cross-attention	2 * 64 * 64 + 64 * 128	16,384
12		Residual connection and layer normalization	64	64
13		FNN	64 * 64 * 2	8,192
14		Residual connection and layer normalization	64	64
15		Layer normalization	64	64
16	Output layer	Full connected	64 * 1	64
17		Full connected	20 * 1	20

Supplementary Table 10: Model parameter (hidden layer dimension: 32).

Index		Layer	Weight parameters	Total trainable parameters
1	Encoder module	Token embedding	400 * 32 * 3	38,400
2		Flash self-attention	32 * 96 + 32 * 32	4,096
3		Residual connection and layer normalization	32	32
4		FNN	32 * 32 * 2	2,048
5		Residual connection and layer normalization	32	32
6		1-D CNN	3 * 32 * 32 + 32	3,104
7		Layer normalization	32	32
8	Decoder module	Token embedding	400 * 32 * 3	38,400
9		Masked probsparse self-attention	4 * 32 * 32	4,096
10		Residual connection and layer normalization	32	32
11		Flash cross-attention	2 * 32 * 32 + 32 * 64	4,096
12		Residual connection and layer normalization	32	32
13		FNN	32 * 32 * 2	2,048
14		Residual connection and layer normalization	32	32
15		Layer normalization	32	32
16	Output layer	Full connected	32 * 1	32
17		Full connected	20 * 1	20

Supplementary Table 11: Model parameter (hidden layer dimension: 16).

Index		Layer	Weight parameters	Total trainable parameters
1	Encoder module	Token embedding	400 * 16 * 3	19,200
2		Flash self-attention	16 * 48 + 16 * 16	1,024
3		Residual connection and layer normalization	16	16
4		FNN	16 * 16 * 2	512
5		Residual connection and layer normalization	16	16
6		1-D CNN	3 * 16 * 16 + 16	784
7		Layer normalization	16	16
8	Decoder module	Token embedding	400 * 16 * 3	19,200
9		Masked probsparse self-attention	4 * 16 * 16	1,024
10		Residual connection and layer normalization	16	16
11		Flash cross-attention	2 * 16 * 16 + 16 * 32	1,024
12		Residual connection and layer normalization	16	16
13		FNN	16 * 16 * 2	512
14		Residual connection and layer normalization	16	16
15		Layer normalization	16	16
16	Output layer	Full connected	16 * 1	16
17		Full connected	20 * 1	20

Supplementary Table 12: Model parameter (hidden layer dimension: 8).

Index		Layer	Weight parameters	Total trainable parameters
1	Encoder module	Token embedding	400 * 8 * 3	9,600
2		Flash self-attention	8 * 24 + 8 * 8	256
3		Residual connection and layer normalization	8	8
4		FNN	8 * 8 * 2	128
5		Residual connection and layer normalization	8	8
6		1-D CNN	3 * 8 * 8 + 8	200
7		Layer normalization	8	8
8	Decoder module	Token embedding	400 * 8 * 3	9,600
9		Masked probsparse self-attention	4 * 8 * 8	256
10		Residual connection and layer normalization	8	8
11		Flash cross-attention	2 * 8 * 8 + 8 * 16	256
12		Residual connection and layer normalization	8	8
13		FNN	8 * 8 * 2	128
14		Residual connection and layer normalization	8	8
15		Layer normalization	8	8
16	Output layer	Full connected	8 * 1	8
17		Full connected	20 * 1	20

Supplementary Table 13: Model parameter (hidden layer dimension: 4).

Index		Layer	Weight parameters	Total trainable parameters
1	Encoder module	Token embedding	400 * 4 * 3	4,800
2		Flash self-attention	4 * 12 + 4 * 4	64
3		Residual connection and layer normalization	4	4
4		FNN	4 * 4 * 2	32
5		Residual connection and layer normalization	4	4
6		1-D CNN	3 * 4 * 4 + 4	52
7		Layer normalization	4	4
8	Decoder module	Token embedding	400 * 4 * 3	4,800
9		Masked probsparse self-attention	4 * 4 * 4	64
10		Residual connection and layer normalization	4	4
11		Flash cross-attention	2 * 4 * 4 + 4 * 8	64
12		Residual connection and layer normalization	4	4
13		FNN	4 * 4 * 2	32
14		Residual connection and layer normalization	4	4
15		Layer normalization	4	4
16	Output layer	Full connected	4 * 1	4
17		Full connected	20 * 1	20

Supplementary Table 14: Prediction accuracy and computational cost under different model parameters.

Model parameters (M)	RMSE (cycles)	R2	WMAPE	MAE (cycles)	Inference time (s)	FLOPs	Energy (J)	Energy (Wh)
1.869	113	0.960	0.09	81.6	0.103	1649224715	46.35	0.0129
0.623	106	0.965	0.08	72.9	0.090	526178162	40.50	0.0113
0.234	112	0.960	0.09	81.2	0.091	188480805	40.95	0.0114
0.098	115	0.958	0.08	78.1	0.096	75588607	43.20	0.0120
0.044	130	0.946	0.10	96.0	0.084	33131628	37.80	0.0105
0.021	137	0.940	0.12	108.0	0.088	15400418	39.60	0.0110
0.010	132	0.945	0.11	100.3	0.091	7409133	40.95	0.0114

Supplementary Note 4.0 Pruning-Based Complexity Reduction Analysis

After the hidden dimension compression experiments, pruning experiments were further conducted from the perspective of module level structural redundancy to verify that reducing computational complexity does not rely solely on hidden dimension scaling. FFN-P100 denotes complete pruning of the feed forward network module in the Transformer, and was used to evaluate the effects of FFN redundancy reduction on parameter count, FLOPs, inference time and prediction accuracy. ESH, DSH and DCH represent the best pruning configurations for encoder self-attention heads, decoder self-attention heads and decoder cross attention heads, respectively. FFN+ESH, FFN+DSH and FFN+DCH denote joint pruning schemes in which attention head pruning is further applied after FFN pruning. All pruned models were evaluated on the same test set using RMSE, R^2, WMAPE, MAE, parameter count, FLOPs, inference time and energy consumption.

Fig. S15 shows the prediction accuracy distributions of different pruning schemes across 20 test batteries. The results reveal clear differences in the effects of pruning different modules. FFN-P100 maintained favorable predictive accuracy while reducing model complexity, with RMSE, R^2, WMAPE and MAE values of 106 cycles, 0.965, 0.08 and 72.3 cycles, respectively, outperforming the proposed model with 113 cycles, 0.960, 0.09 and 81.6 cycles. This indicates compressible redundancy in the FFN module. Moderate FFN pruning can reduce redundant parameters and may also alleviate overfitting, thereby improving generalization on the test set. By contrast, attention head pruning had a stronger effect on prediction accuracy. For ESH, DSH and DCH, RMSE increased to approximately 157 to 164 cycles, whereas R^2 decreased to approximately 0.915 to 0.922. This suggests that encoder self-attention heads, decoder self-attention heads and decoder cross attention heads all contribute to degradation feature interaction, temporal dependency modelling and lifetime information transfer. Joint pruning further increased the accuracy loss, with RMSE

increasing to approximately 184 to 195 cycles and R^2 decreasing to approximately 0.880 to 0.894. These results indicate that simultaneously weakening the nonlinear mapping capacity of the FFN and the feature interaction capacity of the attention structure substantially constrains the ability of the model to represent complex battery degradation trajectories. Thus, pruning is more than a simple structural removal operation, as different modules contribute differently to prediction performance and structural redundancy.

Fig. S16 and **Table S15** further evaluate the lightweighting effects of different pruning schemes from the perspective of computational cost. Compared with the proposed model, FFN-P100 reduced the parameter count from 1.869 M to 1.606 M, decreased FLOPs from approximately 1.65 billion to approximately 0.75 billion, shortened inference time from 0.103 s to 0.091 s and reduced energy consumption accordingly. This indicates that the FFN is an important source of both parameters and computation, and pruning this module can reduce parameter scale, floating point operations and runtime overhead simultaneously. Attention head pruning had limited effects on parameter count and FLOPs. ESH, DSH and DCH largely retained the same parameter scale and FLOPs as the original model, but markedly reduced inference time, with the lowest value reaching approximately 0.080 s. This suggests that the main benefit of attention head pruning lies in simplifying the inference pathway and improving practical runtime efficiency, rather than explicitly reducing parameter count or FLOPs. This result also indicates that FLOPs and inference latency are not fully equivalent, since the execution pattern, parallel efficiency and computational scheduling of attention structures also affect actual runtime.

The joint pruning results further clarify the sources of complexity reduction across modules. FFN+ESH, FFN+DSH and FFN+DCH showed parameter counts and FLOPs similar to FFN-P100, indicating that reductions in these two metrics mainly originated from FFN pruning. After additional attention head pruning, inference time decreased further, but the loss of prediction accuracy became more pronounced. Overall, the pruning experiments demonstrate that module level structural redundancy reduction can effectively reduce model complexity and complement the performance complexity trade off analysis based on hidden dimension compression. FFN pruning can reduce parameter count, FLOPs, inference time and energy consumption while maintaining or even improving prediction accuracy. Attention head pruning mainly improves practical inference efficiency, whereas joint pruning can further reduce runtime overhead but is more likely to degrade prediction performance. Therefore, pruning design for battery lifetime prediction should balance complexity reduction with the preservation of degradation feature representation capacity.

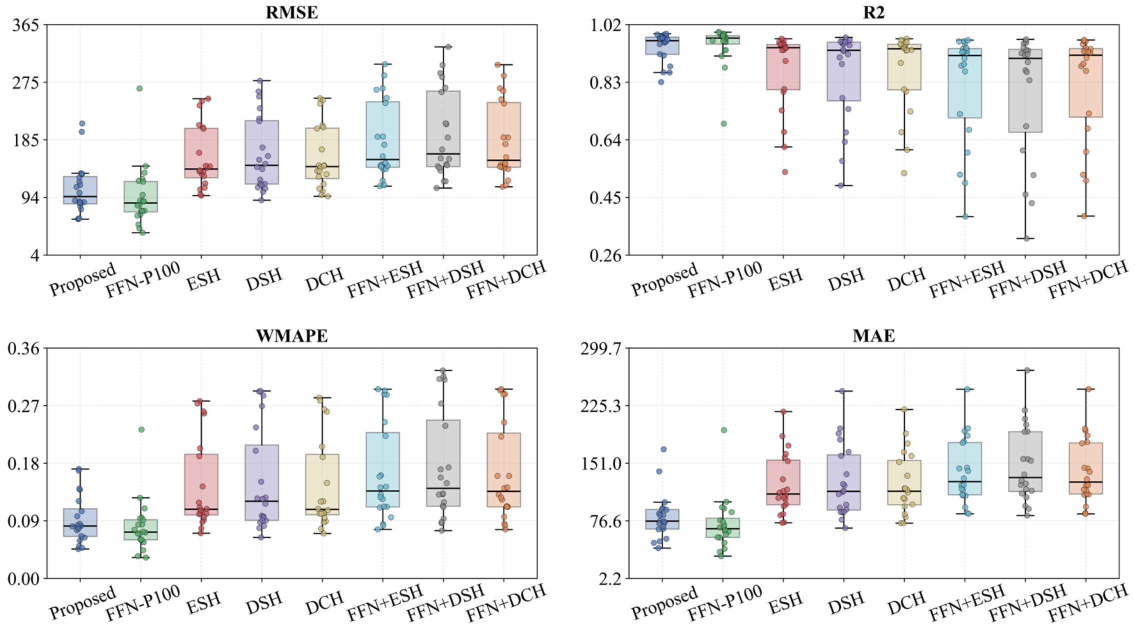

Supplementary Figure 15: Prediction accuracy distributions of different pruning schemes. Box-and-scatter plots of RMSE, R^2, WMAPE, and MAE across 20 test batteries for the proposed model and seven pruning variants, including FFN-P100, ESH, DSH, DCH, FFN+ESH, FFN+DSH, and FFN+DCH. Each scatter point represents the prediction result of one test battery. The box shows the interquartile range, the horizontal line denotes the median, and the whiskers indicate the data spread.

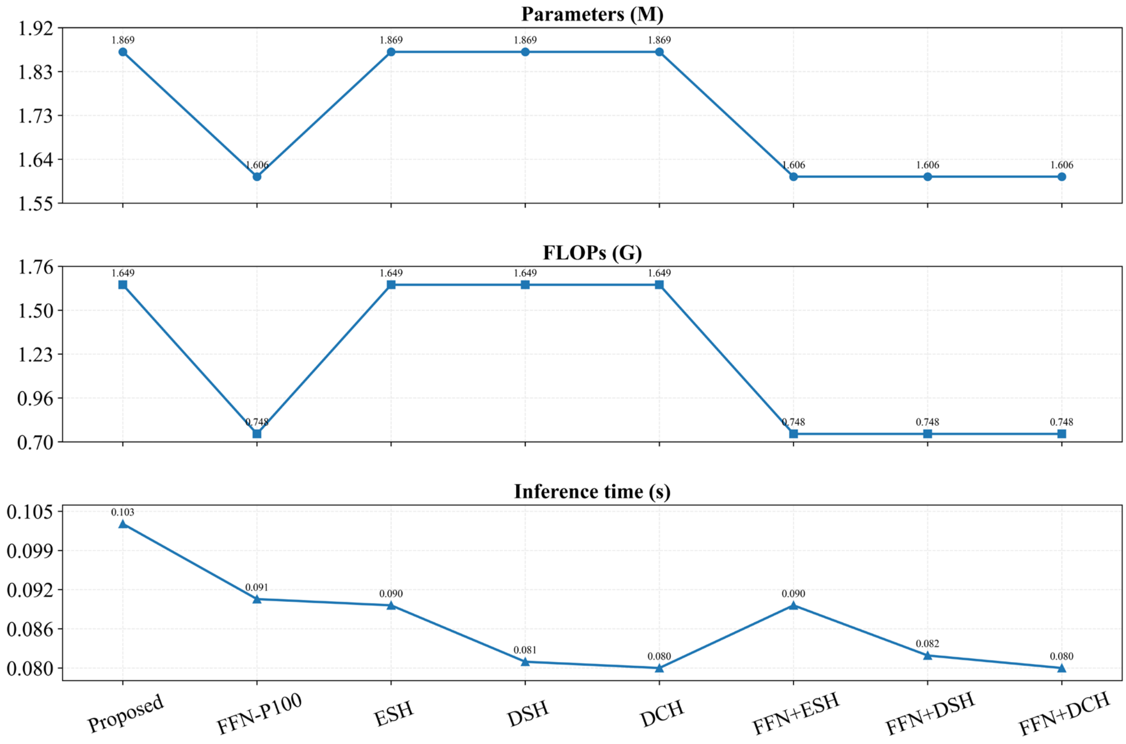

Supplementary Figure 16: Computational complexity comparison of different pruning schemes. Line plots of model parameters, FLOPs, and inference time for the proposed model and seven pruning variants, including FFN-P100, ESH, DSH, DCH, FFN+ESH, FFN+DSH, and FFN+DCH.

Supplementary Table 15: Pruning scheme evaluation based on prediction accuracy and computational complexity.

Pruning schemes	Model parameters (M)	RMSE (cycles)	R2	WMAPE	MAE (cycles)	Inference time (s)	FLOPs	Energy (J)	Energy (Wh)
Proposed	1.869	113	0.960	0.09	81.6	0.103	1649224715	46.35	0.0129
FFN-P100	1.606	106	0.965	0.08	72.3	0.091	748134813	40.95	0.0114
ESH	1.869	157	0.922	0.13	119.1	0.090	1649224715	40.50	0.0113
DSH	1.869	164	0.915	0.13	122.2	0.081	1649224715	36.45	0.0101
DCH	1.869	158	0.921	0.13	120.1	0.080	1649224715	36.00	0.0100
FFN+ESH	1.606	184	0.893	0.14	135.3	0.090	748134813	40.50	0.0113
FFN+DSH	1.606	195	0.880	0.15	142.9	0.082	748134813	36.90	0.0103
FFN+DCH	1.606	184	0.894	0.14	135.3	0.080	748134813	36.00	0.0100

Supplementary Note 4.1 Input Feature Construction and Attention-Aware Analysis

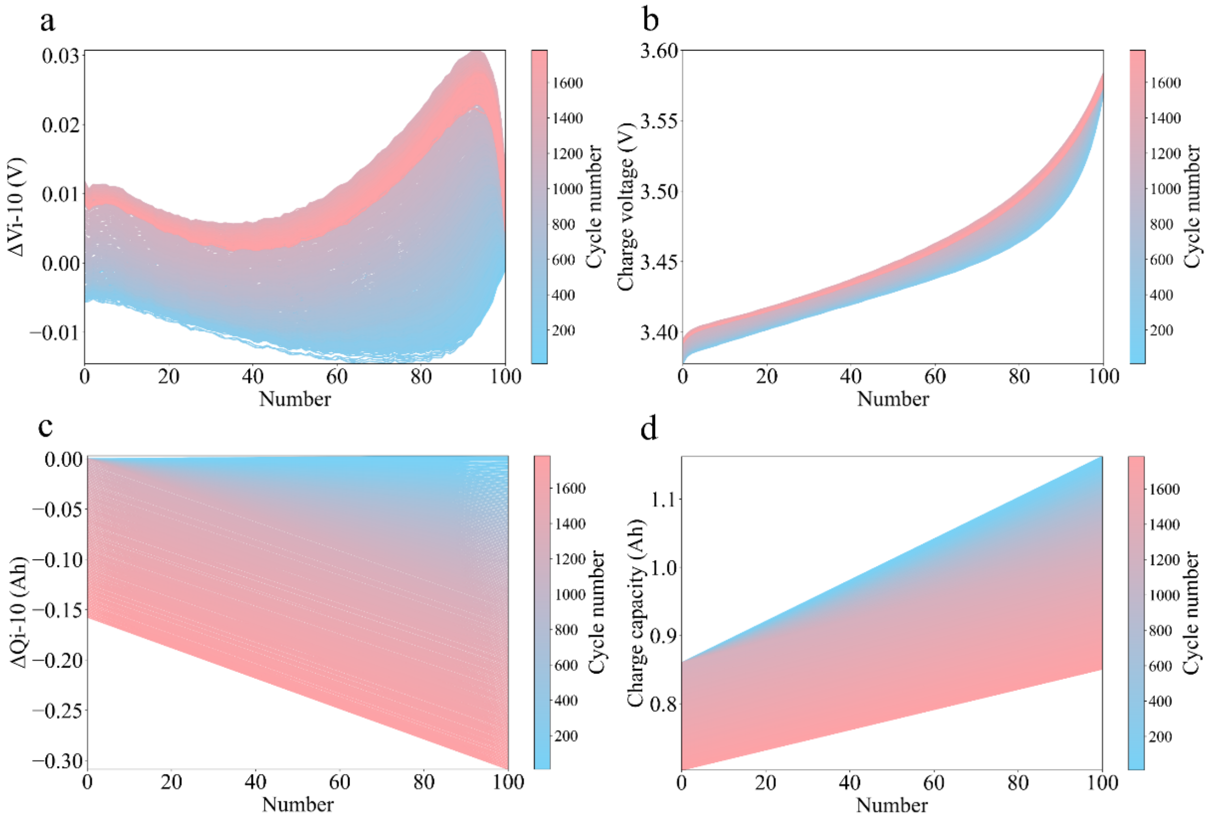

Supplementary Figure 17: Four charging characteristic curves. (a) Charging voltage curve differences for each cycle relative to the tenth cycle. (b) Charging voltage curves. (c) Charging capacity curve differences for each cycle relative to the tenth cycle. (d) Charging capacity curves.

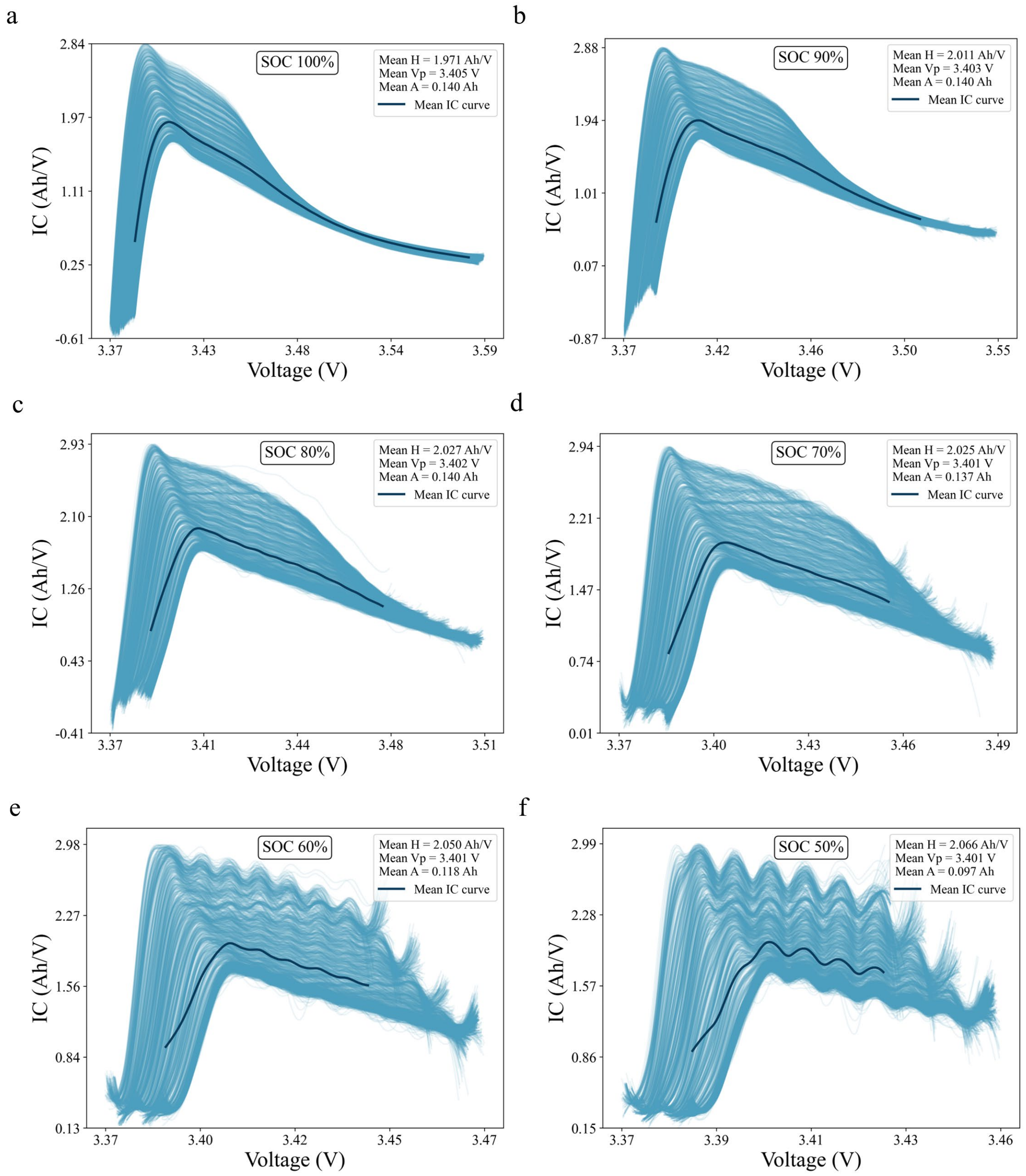

Supplementary Figure 18: Incremental capacity curves under different retained proportions of the high SOC charging window. (a)-(f) shows the IC curves of the same representative cell when retaining 100%, 90%, 80%, 70%, 60%, and 50% of the charging information within the high SOC window from approximately 80% SOC to the 3.6 V cutoff voltage. Light blue curves represent IC curves from individual cycles, while the black curve denotes the mean IC profile. The inset statistics report the mean peak height, peak voltage, and peak area for each retained proportion.

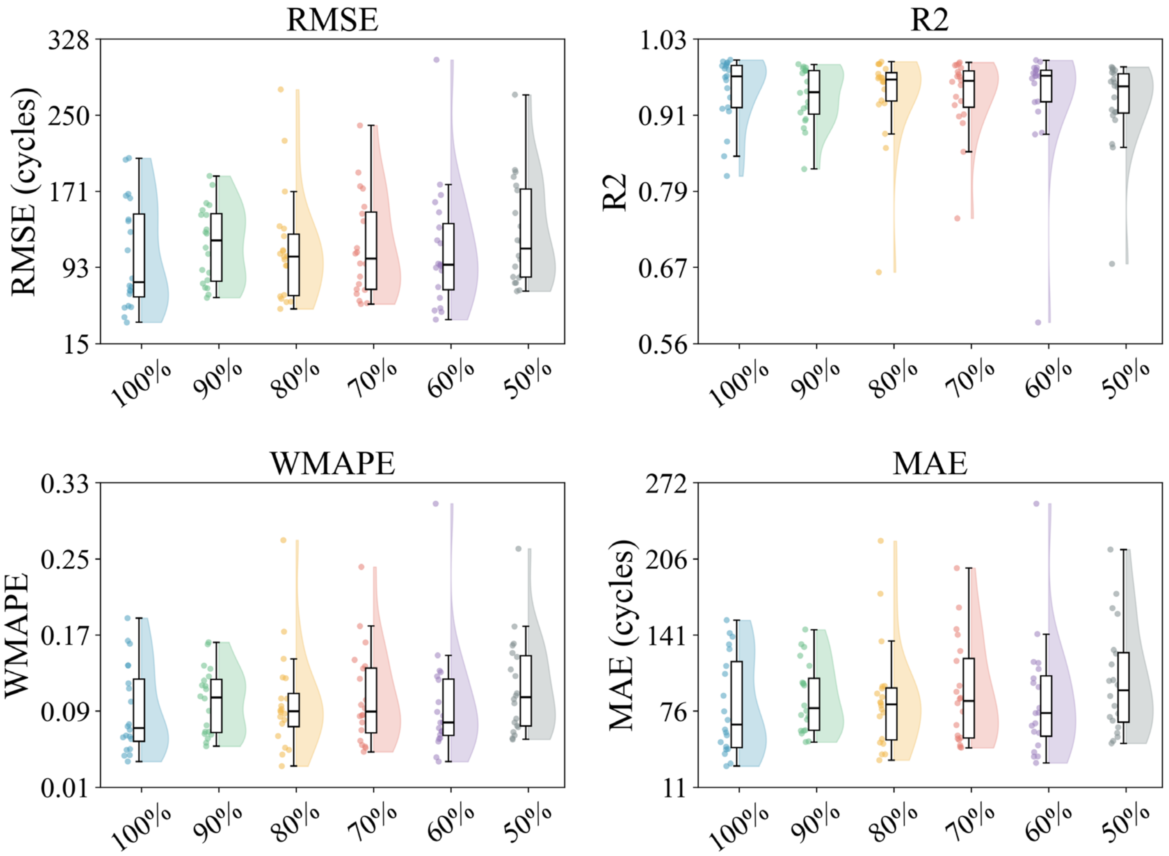

Supplementary Figure 19: Raincloud plots of model prediction performance under different retained proportions of the high SOC charging window. The distributions of RMSE, R^2, WMAPE, and MAE are shown when retaining 100%, 90%, 80%, 70%, 60%, and 50% of the charging information within the high SOC window from approximately 80% SOC to the 3.6 V cutoff voltage. Scatter points denote individual test cells, half-violin plots indicate metric distributions, and boxplots show the median and interquartile range.

Supplementary Table 16: Model performance and electrochemical feature statistics under different retained proportions of the high SOC charging window.

Retained proportion	RMSE (cycles)	R2	WMAPE	MAE (cycles)	Mean H (Ah/V)	Peak voltage (V)	Peak area (Ah)
100%	105	0.948	0.0885	80.2	1.971	3.405	0.140
90%	117	0.939	0.0979	87.3	2.011	3.403	0.140
80%	171	0.928	0.1230	136	2.027	3.402	0.140
70%	114	0.941	0.1020	91.4	2.025	3.401	0.137
60%	111	0.941	0.0943	85.2	2.050	3.401	0.118
50%	128	0.928	0.1130	102	2.066	3.401	0.097

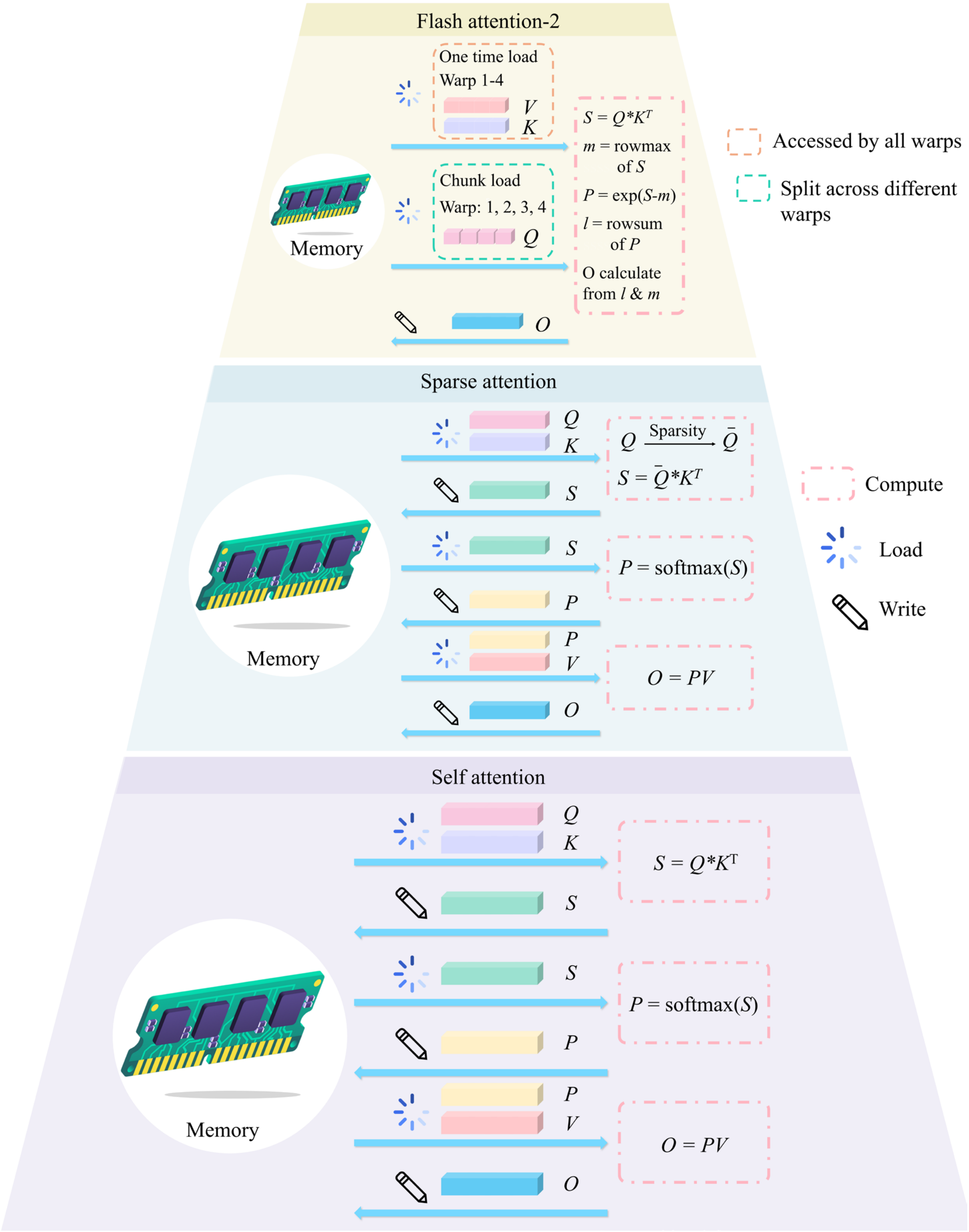

Supplementary Figure 20: Comparing the computation and memory loading methods of three attention mechanisms: Flash attention-2, sparse attention, and vanilla self-attention. Flash attention-2 reduces memory access and computational overhead by loading Q, K, and V in blocks and optimizing the SoftMax operation. Sparse attention reduces the computational burden by simplifying the matrix multiplication of Q and K. In contrast, vanilla self-attention requires multiple memory read and write operations, resulting in higher computational complexity.

Supplementary Note 4.2 Hyperparameter Sensitivity Analysis

A hyperparameter sensitivity analysis was conducted to evaluate the stability and robustness of the model under different training configurations. The model architecture, training set size and test set size were kept unchanged, while the batch size, learning rate and attention dropout rate in the flash attention mechanism were varied. Specifically, batch sizes of 128, 256, 512 and 768 were evaluated with corresponding learning rates of 1e-4, 1.5e-4, 2e-4 and 2.5e-4, and the attention dropout rate was varied from 0.2 to 0.6. All configurations were evaluated on the same 20 test samples using four metrics, namely RMSE, R^2, WMAPE and MAE, to quantify the sensitivity of predictive performance to changes in training setting (**Fig. S21**). The results show that the model maintained generally stable predictive performance across the tested hyperparameter configurations. For most test samples, RMSE, WMAPE and MAE remained within comparable ranges, while R^2 was mainly concentrated above 0.9. This indicates that the model was relatively insensitive to moderate variations in batch size, learning rate and attention dropout rate. Under each fixed batch size, adjacent dropout settings produced similar fluctuation patterns across the 20 test samples, suggesting that attention dropout had a limited effect on the overall prediction trend. Across different batch sizes, no clear monotonic degradation in model performance was observed. With batch sizes of 128 and 256, the error distributions were relatively compact, and R^2 remained high for most samples, indicating stable training behaviour under small and medium batch settings. When the batch size increased to 512 and 768, the model still retained acceptable predictive accuracy, although several test samples showed locally increased RMSE, WMAPE and MAE, accompanied by slight decreases in R^2. These results suggest that larger batch sizes combined with higher learning rates may amplify sample dependent error fluctuations, but do not lead to systematic performance failure. Overall, the sensitivity analysis demonstrates that the model preserved robust predictive performance across a broad range of training configurations. The close agreement between curves obtained with different dropout settings further indicates that the model does not strongly depend on a specific attention dropout value. This robustness is beneficial for practical deployment, as it reduces the need for extensive hyperparameter tuning and supports stable prediction under different training configurations.

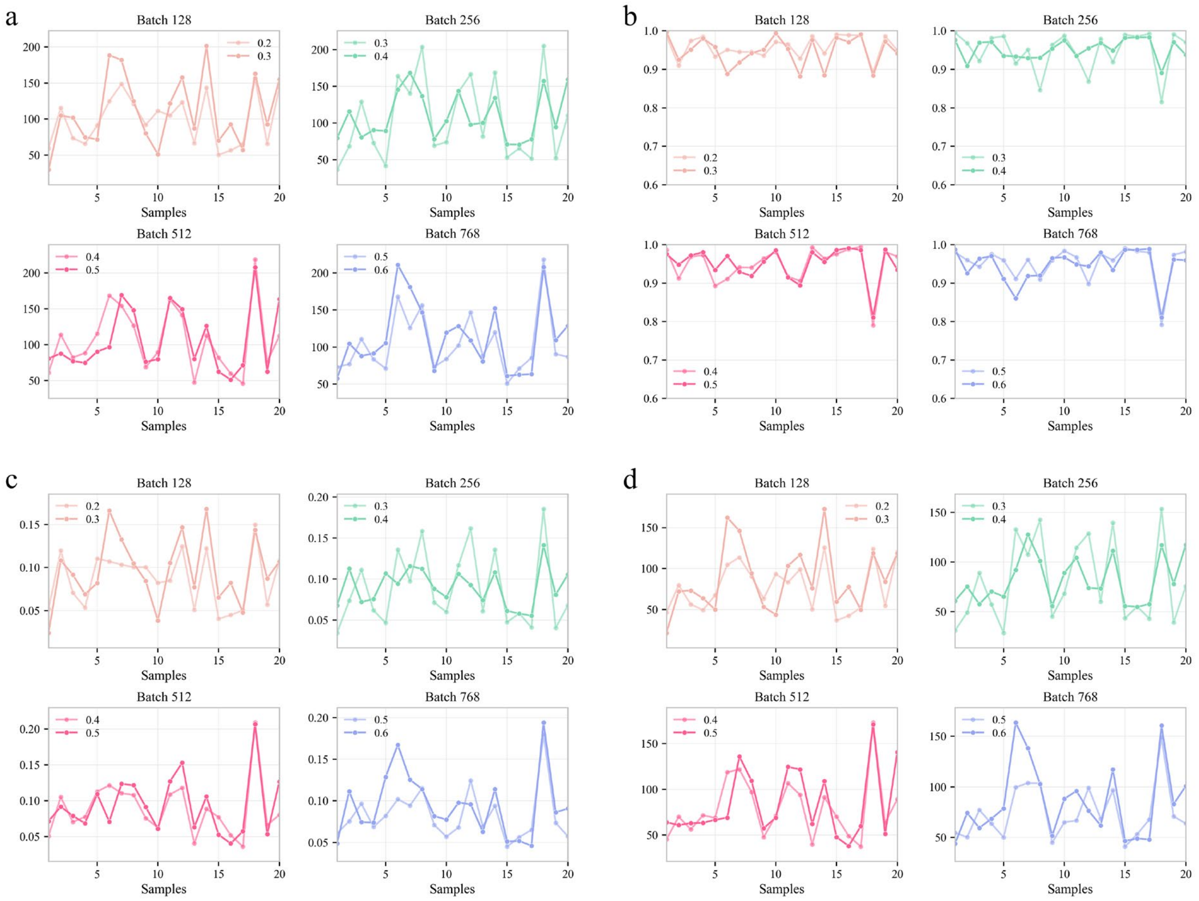

Supplementary Figure 21: Hyperparameter sensitivity analysis of model performance. Subplots (a–d) illustrate the variations in prediction performance across 20 test samples under different combinations of batch size (128, 256, 512, 768), learning rate (1e-4, 1.5e-4, 2e-4, and 2.5e-4), and attention dropout rate (0.2–0.6). The four metrics reported are: (a) RMSE, (b) R^2, (c) WMAPE, and (d) MAE. Each panel visualizes the performance distribution under two dropout settings for a fixed batch size, highlighting the model's sensitivity to regularization intensity and data batching in training dynamics.

Supplementary Note 4.3 Training Configuration and Hyperparameter Settings

During the model training phase, the optimization of activation functions and certain adjustable hyperparameters can significantly accelerate model convergence, even when there is a high signal-to-noise ratio in the training dataset. To further enhance model performance, the grid search method was used to systematically optimize and set key hyperparameters, including the learning rate. This approach ensures that parameters are adjusted in a fine-grained manner during training, leading to more stable and accurate predictions. The hyperparameters used in this study, along with their configurations, are shown in **Table S17**. These hyperparameters played a key role in ensuring stability and improving predictive performance.

Supplementary Table 17: Hyperparameters setting.

Parameters		Value
The dimensionality of the model		256
Dimensionality of the feedforward network		256
The number of attention heads		4
Number of decoder and encoder layers		1
Optimizer		Adam
Number of epochs		10000
Early stopping patience		1200
Activation function	Encoder layer	GELU
	Decoder layer	GELU
	Full connected output layer	Sigmoid
Dropout rate	Flash attention layer	0.2
	Sparse attention layer	0.3
Learning rate	Learning rate for FAST-BatPro main experiments	0.0002
	Learning rate for baseline-model comparison	0.0008

Supplementary Note 4.4 Weight Optimization

Weight optimization is a crucial step in selecting model parameters. The main goal of continuously training on the dataset is to find the optimal parameter values that minimize the cost function, thereby improving model performance. The cost function was defined using the mean squared error, as follows:

$$\mathrm{MSE}(y,\hat{y}) = \frac{\lambda}{n}\sum_{i=1}^{n}\left(y_i - \hat{y}_i\right)^2, \tag{6}$$

where y_i represents the true values, $\hat{y}_i$ refers to the predicted values, n is the number of samples, and λ denotes the loss weights.

The Adam optimization algorithm was used to update the parameters of each layer in the model. Adam optimizer adjusts the learning rate adaptively by calculating first- and second-order moment estimates of the gradients, allowing for more efficient parameter optimization. The key steps include: calculating the gradient, updating the exponentially weighted averages of the momentum term (m_t) and squared gradients (vt), applying bias correction (for first- and second-order moment estimates), and finally updating the model parameters according to the following formulas.

$$\begin{cases} g_t = \nabla_\theta J(\theta_{t-1}) \\ m_t = \beta_1 m_{t-1} + (1-\beta_1)g_t \\ vt = \beta_2 v_{t-1} + (1-\beta_2)g_t^2 \\ \hat{m}_t = \dfrac{m_t}{1-\beta_1^t} \\ \hat{v}_t = \dfrac{v_t}{1-\beta_2^t} \\ \theta_t = \theta_{t-1} - lr\dfrac{\hat{m}_t}{\sqrt{\hat{v}_t}+\varepsilon} - \omega\theta_{t-1} \end{cases}, \tag{7}$$

where g_t is the gradient, θ_t represents the parameters, lr is the learning rate, set to 0.0002, β_1 and β_2 are the decay rates for the momentum term and squared gradients, set to 0.9 and 0.999, respectively, ε is a constant to prevent division by zero, ω is the weight decay coefficient, set to 0.
